# Degradable *N*-Vinyl Copolymers through Radical Ring-Opening Polymerization of Cyclic Thionocarbamates

**DOI:** 10.1021/acsmacrolett.4c00550

**Published:** 2024-10-07

**Authors:** Alvaro Calderón-Díaz, Andrew C. Boggiano, Wei Xiong, Nadine Kaiser, Will R. Gutekunst

**Affiliations:** † School of Chemistry and Biochemistry, Georgia Institute of Technology, Atlanta, Georgia 30332, United States; ‡ BASF SE, Group Research, Carl Bosch Str 38, 67056 Ludwigshafen, Germany

## Abstract

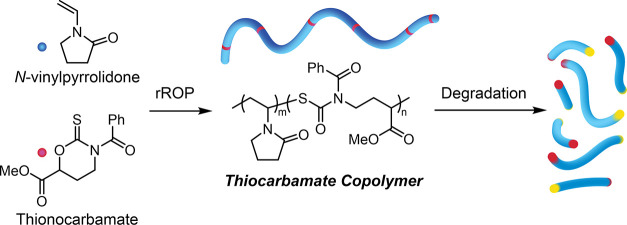

A thiocarbonyl radical ring-opening polymerization approach was implemented with cyclic thionocarbamates to generate degradable copolymers with *N*-vinyl monomers. The rigid structures of cyclic *N*-substituted thionocarbamates have been revealed by X-ray crystallography and NMR spectroscopy. The corresponding copolymers show incorporation of the thiocarbamates within the carbon backbone of polyvinylpyrrolidone influenced by acyl substituents through radical ring-opening copolymerization. The phenyl-substituted cyclic thionocarbamate copolymerized with *N*-vinyl carbazole and *N*-vinyl caprolactam, while little to no incorporation occurred with ^*t*^Bu acrylate and styrene, respectively. Further, these copolymers can undergo hydrolytic degradation under mild conditions. A new family of cyclic thionocarbamates capable of radical ring-opening copolymerization with *N*-vinyl monomers has been established.

Cyclic thiocarbonyl compounds have recently become attractive monomers for the radical polymerization of degradable polymers.^1,2^ These moieties provide an advantage when compared to the analogous cyclic ketene acetals (CKAs)^3^ because of their stability under ambient conditions, synthetic versatility, reduced side reactions during polymerization, and the thermodynamically favored formation of the carbonyl group upon ring opening. For example, considerable attention has been placed on the copolymerization of dibenzo[*c,e*]oxepane-5(7*H*)-thione (DOT) with more activated monomers by radical ring-opening methods to install degradable thioester linkages in the backbone (Scheme 1A).^1^ The incorporation of this thionolactone within the C–C backbone of common polymers has also been demonstrated to produce versatile degradable architectures such as linear polymers,^4−11^ nanoparticles,^12−15^ micelles,^16^ bottlebrushes,^17^ and polymer networks.^18−21^ While more activated monomers have been successfully copolymerized with thionolactones, copolymerization with less activated monomers remains relatively unexplored.^22,23^

**Scheme 1:**
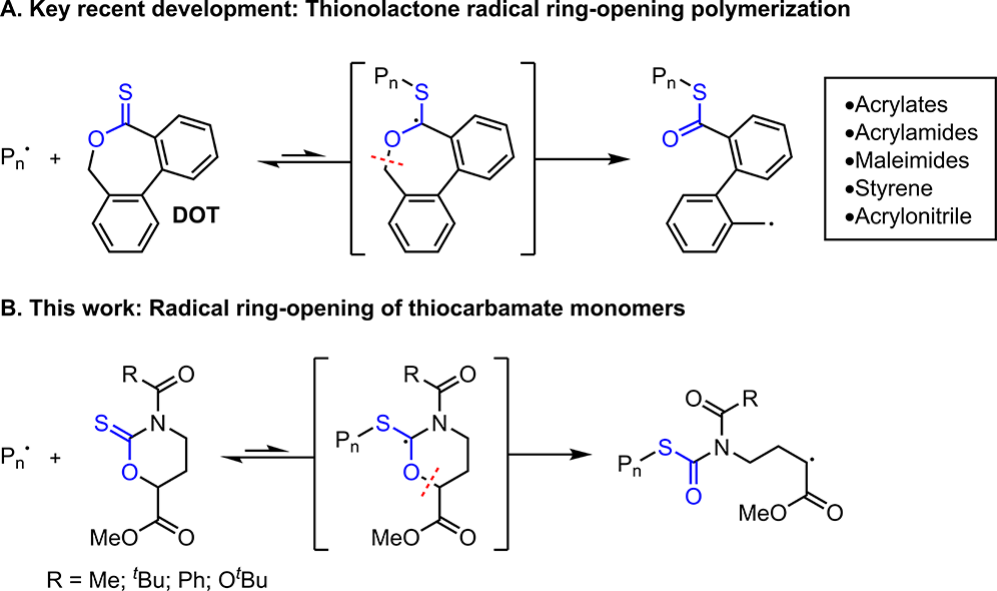
Radical Ring-Opening Polymerization of DOT and Common Comonomers (A) and Radical Ring-Opening Polymerization of Cyclic Thionocarbamates (B)

One of the more commercially relevant less activated monomers is *N*-vinylpyrrolidone (**NVP**), and when polymerized into polyvinylpyrrolidone (**PVP**), this polymer possesses various industrial applications with a projected market value of $4.9 billion USD by 2030.^24^ A challenge to create degradable **PVP** copolymers results from the highly reactive **NVP** radical intermediate produced when compared to the stabilized radicals of more activated monomers (e.g., acrylates, acrylamide, styrene). Therefore, it is important to identify comonomer structures with more stabilized thiocarbonyl units to obtain random copolymerization. Recently, the Coughlin group was able to demonstrate that the uniform incorporation of ester fragments within a **PVP** backbone can be achieved through semibatch polymerization of CKAs to form degradable **PVP** copolymers.^25^ Batch copolymerization of **NVP** with the random incorporation and high conversion of thiocarbonyl monomers within a given system is still of interest to produce degradable **PVP** copolymers.

Insight into controlling the copolymerization of **NVP** can be obtained from reversible addition–fragmentation chain transfer (RAFT). RAFT agents of trithio- and dithioesters are not suitable for controlled polymerization of **NVP** because of the energetic difference between the reactive radical of **NVP** and the stable trithio and dithio radicals. It is, therefore, unsurprising that DOT copolymerizes poorly with **NVP**, such as the observed lack of control with analogous dithioester RAFT agents. Recent advances have been made through RAFT polymerization of **NVP** using xanthates and dithiocarbamates.^26^ Inspired by the versatility of RAFT agents to control the polymerization of various monomers, the design of cyclic thiocarbonyl comonomers resembling dithiocarbamates can be implemented for copolymerization with **NVP** using commercially available precursors for synthetic ease (Scheme 1B).

Herein, the synthesis and characterization of a new family of cyclic thionocarbamates (CTCs) **MeCTC**, ^***t***^**BuCTC**, **PhCTC**, ***p***-**CF**_**3**_–**PhCTC**, ***p***-**MeO**–**PhCTC**, and ^***t***^**BuOCTC** is reported. These monomers can undergo radical ring-opening copolymerization with **NVP** and less activated monomers. Moreover, incorporation of the thiocarbamate moiety within the **PVP** backbone induced degradation in the presence of NaOMe.

The synthesis of CTCs followed a short three-step protocol (Figure 1A). The esterification of *S*-(−)-4-amino-hydroxybutyric acid was carried out by the addition of acetyl chloride into a solution of MeOH to give **I** in an excellent yield (96%). Cyclization of **I** using *N*,*N*′-thiocarbonyldiimidazole in the presence of Et_3_N afforded cyclic thionocarbamate **II** (43%). Two acylation methods of **II** were identified to provide the corresponding target CTCs as a liquid (**MeCTC**) and crystalline solids (^***t***^**BuCTC**, **PhCTC**, ***p***-**CF**_**3**_–**PhCTC**, ***p***-**MeO**–**PhCTC**, and ^***t***^**BuOCTC**) in yields between 47–92%.

**Figure 1:**
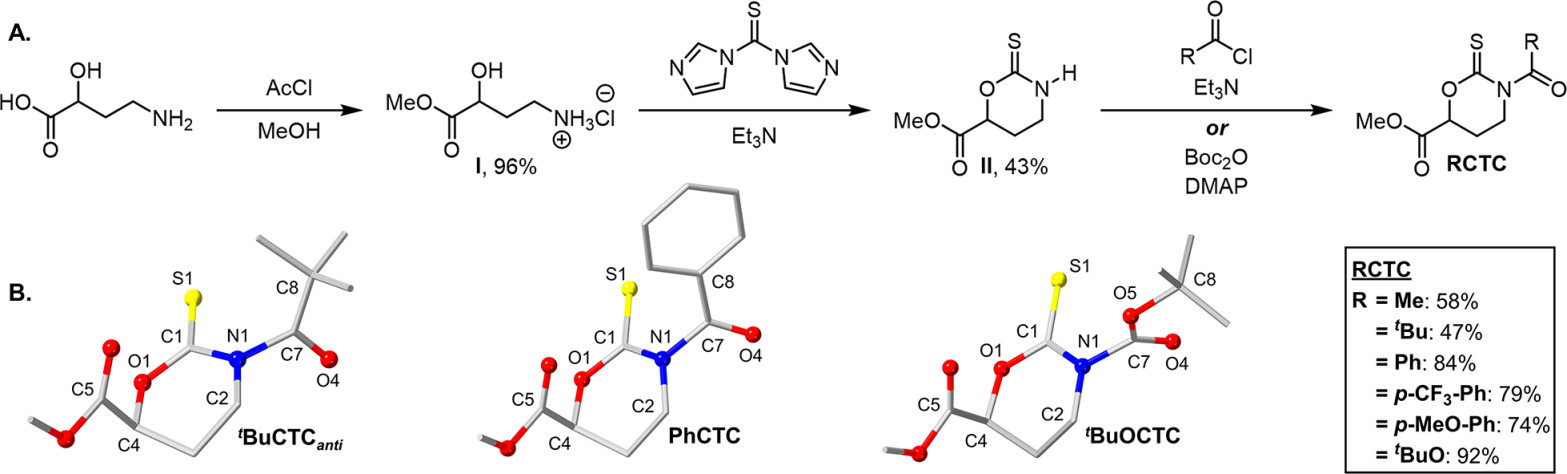
Three-step synthesis of cyclic thiocarbamates (A). Crystal structures of CTCs (B).

To determine the structure and solution conformation of the CTCs, all NMR resonances were completely assigned by 1D and 2D NMR experiments in solutions of CDCl_3_ (Figures S11–S24 and S29–S34). The absence of NOE cross peaks between all CTC acyl and NCH_2_ protons suggested the amide moiety adopts a rigid conformation with the acyl substituent *syn* to the thiocarbonyl functionality, similar to the conformation observed in the crystal structure of a related 1,3-oxazinane-2-thione.^27^ Adoption of this conformation has been known to minimize the net dipole moment in *N*-substituted 1,3-thiazolidine-2-thiones.^28^ NOE cross peaks between the CH and NCH_2_ protons revealed that half-chair conformations in solution are favored with the ester group in the axial position for all CTC monomers prepared, which is consistent with a structurally similar tetrahydrooxazine.^29^

Suitable single crystals of ^***t***^**BuCTC**, **PhCTC**, and ^***t***^**BuOCTC** were grown and subjected to X-ray crystallographic structure determinations (Figure 1B). Single-crystal X-ray diffraction unambiguously highlighted the rigid isostructural configurations of the thionocarbamate monomers with ester groups in the axial positions of the half-chair conformations, consistent with 2D NMR observations. The carbonyl substituents for the **PhCTC** and ^***t***^**BuOCTC** on the amide groups were exclusively *anti* to the ester functionality. Conformational disorder for ^***t***^**BuCTC** showed the carbonyl substituents in the *anti*- and *syn*-configurations with respect to the ester groups in 75% and 25% occupancy, respectively.

The attempts to homopolymerize **MeCTC** were unsuccessful, which can be attributed to a slow ring-opening process or termination of intermediate radicals consistent with other studies.^5^ Further, the reversibility of radical addition may favor the chain end of the ring-opened propagating radical that resembles an acrylate group. Initial examination of CTC copolymerization with *N*-vinyl monomers was carried out using **MeCTC** and **NVP** at a feed ratio of 5:100 in dioxane at 60 °C with azobisisobutyronitrile (AIBN) as the radical initiator for 18 h. The copolymerization afforded a monomodal GPC trace of **MeCTC**-***co***-**PVP** with a *M*_n_ of 13.6 kg/mol (Figure 2A,B). The ^1^H NMR spectrum showed that **NVP** was copolymerized by the appearance of the characteristic peaks for **PVP** between 1.38–2.55 and 3.15–3.85 ppm (Figure S35). The disappearance of the **MeCTC** −CH peak at 4.95 ppm and appearance of small broad peaks between 4.82–6.96 ppm were clear indications that **MeCTC** was incorporated into the polymer. High conversion values were calculated by crude ^1^H NMR for **MeCTC** (≥98) and **NVP** (89%) (Figure 2B, entry 1). This initial data suggest that cyclic thionocarbamates with *N*-acyl substituents are good candidates to match the reactivity of propagating **NVP** radicals. Conversely, conversions determined by ^1^H NMR showed that no copolymerization occurred between **II** (0%) and **NVP** (99%, *M*_n_ = 38.7 kg/mol, *Đ* = 3.54), which can be attributed to the nitrogen lone pair conjugated strongly with the thiocarbonyl group. To investigate the degradation of the thiocarbamate groups in the **PVP** backbone, 5 mg of the copolymer was dissolved in CH_2_Cl_2_ (1 mL) and treated with an excess of a 25% w/v NaOMe solution in MeOH with vigorous stirring for 18 h. Subsequent neutralization using 12.1 M HCl and isolation of the copolymer showed that significant degradation had occurred with a final *M*_n_ of 1.1 kg/mol (Figure 2B). The 92% decrease in *M*_n_ is suggestive of random incorporation of the thiocarbamate fragments within the **PVP** backbone.

**Figure 2:**
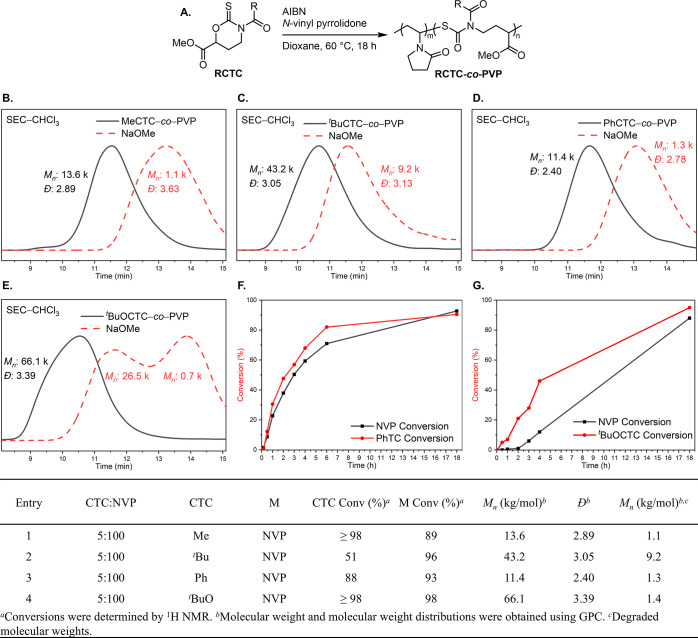
GPC traces of **CTC**-***co***-**PVP** copolymers and degradation profiles (A–E). Degradation traces are shown in red for plots B–E. Conversion of monomers over time were determined by ^1^H NMR (F,G).

When comparing the *M*_n_ of **MeCTC**-***co***-**PVP** to a **PVP** homopolymer (93.5 kg/mol, Figure S53), it was observed that the presence of the thiocarbamates had a decreasing effect on rate of copolymerization, which can be rationalized by the CTC behaving as a reversible deactivating group as is common in their RAFT congeners. The decreased molecular weight may also be a result of hydrolysis or chain transfer events promoted by the thiocarbamate group during polymerization when compared to the robust **PVP** homopolymer.

To determine the influence of the amide substituents on the copolymerization between the CTCs and **NVP**, the Me– group on the CTC was changed to a bulky and electron-rich ^*t*^Bu– group (Figure 2C, entry 2). A significant decrease in conversion of the ^***t***^**BuCTC** (51%) and an increase in the ^***t***^**BuCTC**-***co***-**PVP***M*_n_ (43.2 kg/mol) was observed. The lower conversion for ^***t***^**BuCTC** when compared to **MeCTC** can be attributed to the sterically encumbered approach of the propagating radical toward addition to the thiocarbonyl group. This is justified by the observation of the occupancy disorder observed for ^***t***^**BuCTC** in the solid state. Lower incorporation of the ^***t***^**BuCTC** within the **PVP** backbone in turn can produce longer **PVP** repeat units that result in the increased *M*_n_ of ^***t***^**BuCTC**-***co***-**PVP**. Degradation of ^***t***^**BuCTC**-***co***-**PVP** yielded a degraded polymer with a *M*_n_ of 9.2 kg/mol, which corresponds to a 79% decrease in molecular weight.

Introducing the more electron-deficient **PhCTC** within the backbone of **PVP** while preserving the bulkiness on the amide substituent resulted in the **PhCTC**-***co***-**PVP** with a *M*_n_ of 11.4 kg/mol, comparable to the **MeCTC**-***co***-**PVP** (Figure 2D, entry 3). High conversion values were calculated for **PhCTC** (88%) and **NVP** (93%). The appearance of new thiocarbamate resonances in the ^13^C NMR (δ = 170 ppm) and the absence of thioketal resonances (δ = ∼113 ppm) supports a ring-opening pathway without ring-retaining side reactions (Figure S44). The degradation profile for **PhCTC**-***co***-**PVP** showed a degraded polymer *M*_n_ of 1.3 kg/mol similar to **MeCTC**-***co***-**PVP**. Because **PhCTC** was found to have better shelf stability than **MeCTC**, it was selected as the optimal monomer for further studies (*vide infra*). A kinetics experiment showed that the conversion over time of **PhCTC** and **NVP** were near equal, which further demonstrated the random incorporation with the thiocarbamate moieties within the **PVP** backbone (Figure 2F).

Surprisingly, the largest *M*_n_ was found for ^***t***^**BuOCTC**-***co***-**PVP** at 66.1 kg/mol with high conversion values for both ^***t***^**BuOCTC** (≥98%) and **NVP** (98%) (Figure 2E, entry 4). The ^***t***^**BuOCTC**-***co***-**PVP** showed a high molecular weight shoulder similar to that of the **PVP** homopolymer, which suggested possible compositional drift. Moreover, the ^***t***^**BuOCTC**-***co***-**PVP** degradation trace was bimodal with peak *M*_n_ at 26.5 and 0.7 kg/mol. Measuring conversion over time for the ^***t***^**BuOCTC**-***co***-**PVP** revealed that 21% of the ^***t***^**BuOCTC** was consumed within 2 h, while only 1% of the **NVP** was polymerized during the same period. After 4 h, the ^***t***^**BuOCTC** and **NVP** conversions were 46% and 12%, respectively, which indicated that compositional drift does occur during polymerization of ^***t***^**BuOCTC**-***co***-**PVP** (Figure 2G).

To understand the scope of copolymerization, **PhCTC** was subjected to the standard polymerization conditions with *N*-vinyl carbazole (**NVC**), *N*-vinyl caprolactam (**NVCl**), ^*t*^Bu acrylate (^***t***^**BuA**), and styrene (**Sty**) as comonomers (Figure 3A). Increasing the ring size from 5 (**NVP**) to 7 (**NVCl**) showed a decrease in the vinyl monomer conversion (73%) and an increase in the **PhCTC** conversion (99%) when compared to **PhCTC**-***co***-**PVP** (Figure 3B, entry 2). When using **NVC**, there was a significant decrease in vinyl monomer conversion to 49% (Figure 3B, entry 1). The thionocarbamate conversion could not be determined by ^1^H NMR due to a large overlap of the polymeric carbazole peaks between 4.00–6.50 ppm. As hypothesized, **PhCTC** had a preference to copolymerize with *N*-vinyl monomers when compared to the more activated ^***t***^**BuA** (18% **PhCTC** conversion, Figure 3, entry 3, and Figures S43 and S56) and **Sty** (0% **PhCTC** conversion, Figure 3, entry 4). This may be due to the slow addition of the stabilized acrylate-like radical to the thionocarbamate group, which leads to acrylate homopolymers that lack degradability. Degradation profiles for **PhCTC**-***co***-**PVCl** and **PhCTC**-***co***-**PVC** showed bimodal and monomodal GPC traces, respectively.

**Figure 3:**
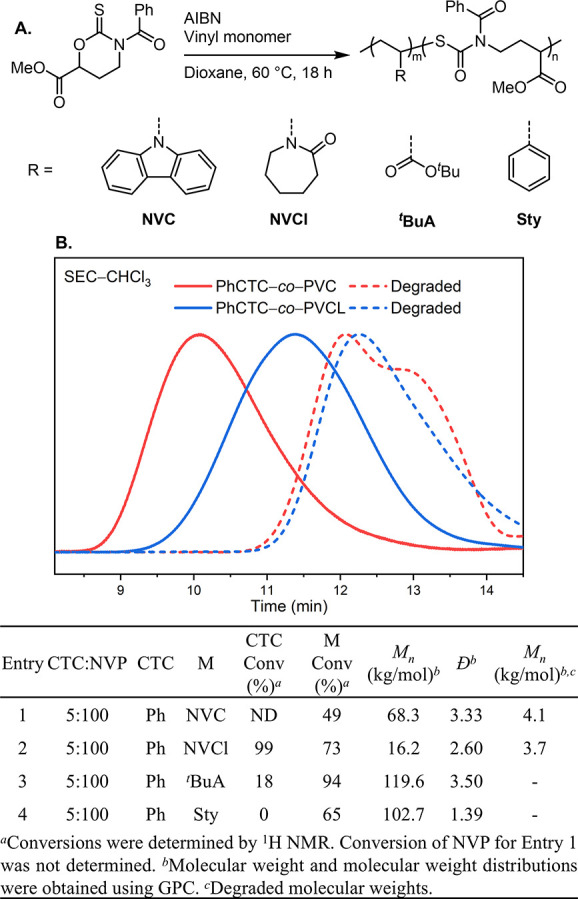
Polymerization of **PhCTC** and vinyl monomers (A). GPC traces of **PhCTC**-***co***-**PVC** and **PhCTC**-***co***-**PVCl** (B). Degradation traces are shown in dashed lines for plot B. Table summary of **PhCTC** copolymerization with vinyl monomers.

To observe how the electronic modification of the nitrogen atom affects the reactivity of the cyclic thionocarbamate system, two derivatives of **PhCTC** were synthesized with either methoxy or trifluoromethyl substituents at the *para* position (Figure 4A). While modest, changes in relative rates of conversion compared to those of **NVP** could be readily observed. Whereas **PhCTC** displays near random copolymerization with **NVP**, ***p***-**CF**_**3**_–**PhCTC** is fully consumed with only 81% conversion of **NVP** (Figure 4B, entry 2). In contrast, ***p***-**MeO**–**PhCTC** led to slower rates of polymerization with only 79% of the cyclic thionocarbamate conversion observed upon complete consumption of **NVP** (Figure 4B, entry 3). This is consistent with the methoxy substituent donating electron density into the aryl amide, which leads to an increased level of resonance stabilization of the thiocarbonyl by the nitrogen atom. This effect results in slower rates of attack by the propagating radical. Conversely, the trifluoromethyl substituent destabilizes the aryl amide, which directs the nitrogen atom’s resonance contribution away from the thiocarbonyl and increases the rate of radical attack. Interestingly, increasing electron density on the *para*-position of the phenyl ring also increased the *M*_n_ of the copolymers from 1.9 to 21.4 kg/mol (Figure 4B, entries 1–3). This is possibly because of a correlation with the rates of chain transfer or adventitious hydrolysis during polymerization.

**Figure 4:**
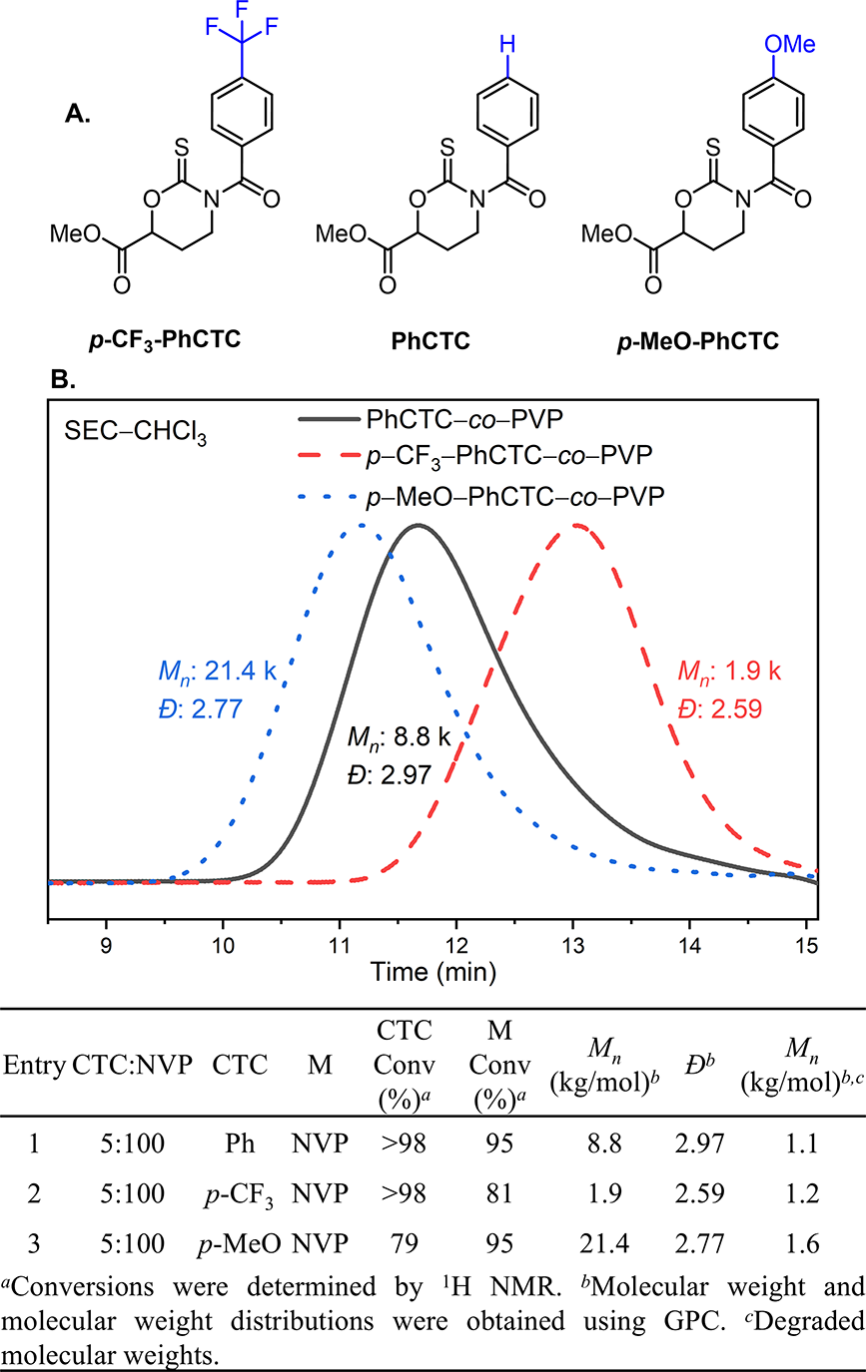
Polymerization of *para*-substituted CTCs (A). GPC traces of *para*-substituted **CTC**-*co*-**PVP** (B). Table summary of *para*-substituted CTC copolymerization with **NVP**.

Initial investigations using the trithiocarbonate RAFT agent DoPAT in combination with **NVP** and **MeCTC** resulted in incomplete conversions for both monomers. The controlled polymerization of the CTCs was then attempted using dithiocarbamate-based RAFT agents (Figure 5, Figures S58 and S60, and Table S14). First, universal RAFT agents **R**_**2**_–**R**_**3**_ were used because they have been proven to produce controlled **PVP** homopolymers.^30,31^ Feed ratios among the CTCs, **NVP**, and the RAFT agents were 5:100:1. Polymerization at 60 °C with AIBN as the radical initiator in dioxane over 16 h resulted in a copolymer *M*_n_ significantly lower than the theoretical value of 11.4 kg/mol. Optimization experiments were unable to produce an increase in the *M*_n_. Monitoring copolymerization over time, it was seen that the copolymer reached near theoretical *M*_n_ between 2–3 h although conversion for **PhCTC** and **NVP** were <15% and <5%, respectively. It was initially believed that the pyridyl groups of **R**_**2**_–**R**_**3**_ were behaving as nucleophiles and attacking the thiocarbamate moiety; therefore, the new **R**_**4**_–**R**_**5**_ RAFT agents were synthesized to avoid the pyridyl groups but resulted in no increase in *M*_n_. The gradual decrease in *M*_n_ observed over time and nonideal RAFT polymerization behavior was indicative of chain transfer.

**Figure 5:**
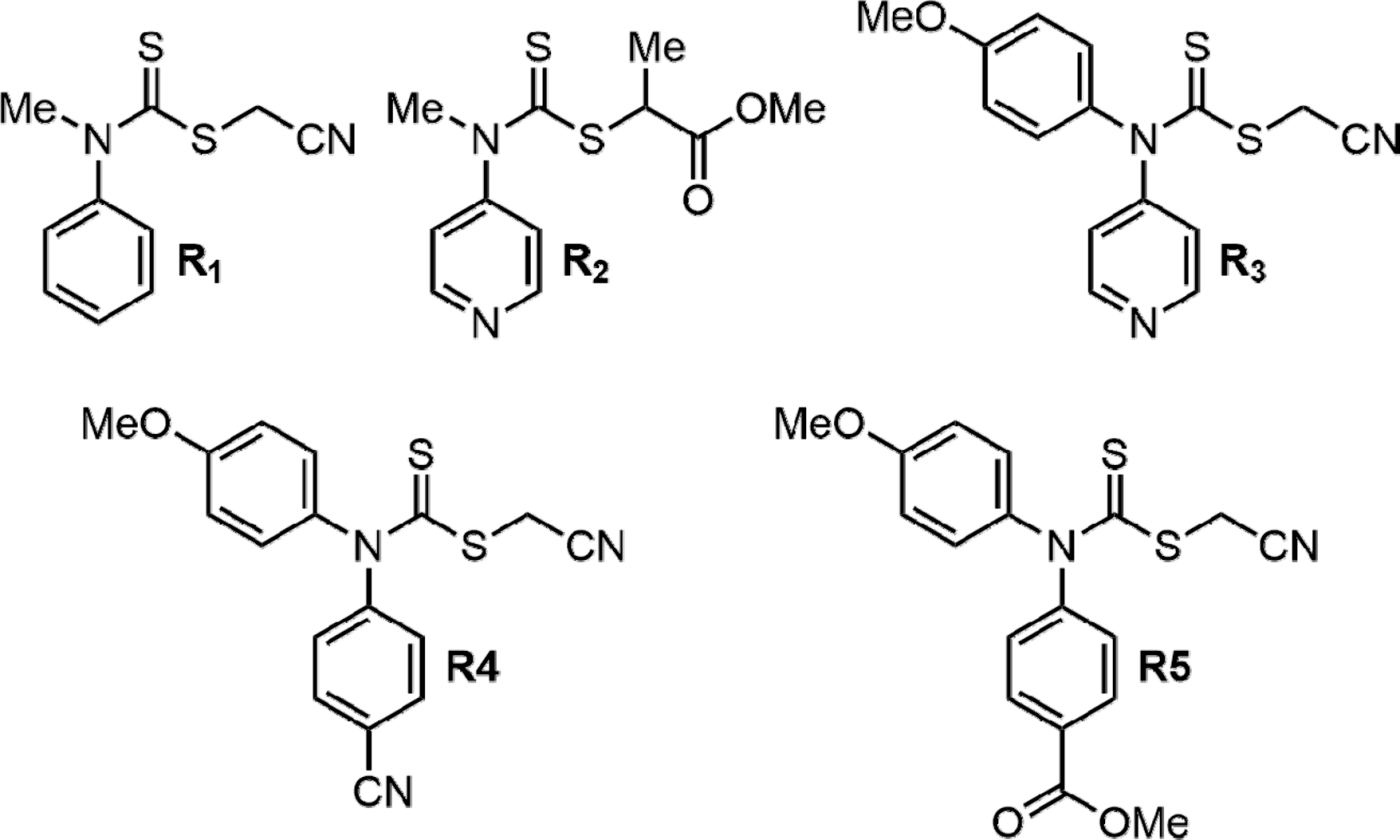
Selected RAFT agent comparison.

In conclusion, a new family of cyclic thionocarbamate monomers has been reported for radical ring-opening copolymerization with **NVP**. X-ray crystallography revealed that the thionocarbamate group and acyl substituents are distorted with the introduction of bulky groups. NMR spectroscopy showed that the sterically encumbered conformation is retained in solution. The optimal monomer, **PhCTC**, was also successfully copolymerized with other *N*-vinyl monomers, while little to no copolymerization occurred with ^*t*^Bu acrylate and styrene, respectively. Moreover, all *N*-vinyl copolymers degraded at room temperature in solutions of NaOMe. This demonstrates that the incorporation of thiocarbamate moieties into the backbone of *N*-vinyl polymers can be degraded in the presence of chemical stimuli. Future projects will be devoted to exploring the CTC tunability to minimize undesired hydrolysis and chain transfer events.

## Supporting Information Available

X-ray data for ^***t***^**BuCTC** (CIF)
X-ray data for **PhCTC** (CIF)
X-ray data for ^***t***^**BuOCTC** (CIF)
Full experimental details, crystallographic data, ^1^H and ^13^C NMR spectra of **II** and CTCs, UV–vis spectra and SEC traces of CTCs (PDF)

## Supplementary Material

mz4c00550_si_001.cif

mz4c00550_si_002.cif

mz4c00550_si_003.cif
