# Supplementary material for: Degradable N-Vinyl Copolymers through Radical Ring-Opening Polymerization of Cyclic Thionocarbamates
Source: ACS Macro Lett. 2024 Oct 7;13(11):1390–5. doi: 10.1021/acsmacrolett.4c00550 (PMC11580385; doi:10.1021/acsmacrolett.4c00550)
Supplement: Supplementary file 4 — mz4c00550_si_004.pdf [file mz4c00550_si_004.pdf]

Supporting Information

Degradable *N*-Vinyl Copolymers through the Radical Ring-Opening Polymerization of Cyclic Thiocarbamates

Alvaro Calderón-Díaz,† Andrew C. Boggiano,† Wei Xiong,†
Nadine Kaiser‡ and Will R. Gutekunst*,†

†School of Chemistry and Biochemistry, Georgia Institute of Technology, 901 Atlantic Drive NW, Atlanta, Georgia 30332, United States. ‡BASF SE, Group Research, Carl Bosch Str 38, 67056 Ludwigshafen, Germany.

*E-mail: willgute@gatech.edu

Table of Contents

General Methods S3
Synthesis of 3-Hydroxy-4-methoxy-4-oxobutan-1-aminium chloride (**I**) S4
Synthesis of Methyl-2-thioxo-1,3-oxazinane-6-carboxylate (**II**) S4
General Procedure for the Preparation of **CTCs** S4
Synthesis of ***t*BuOCTC** S7
Scheme S1: Synthesis of Methyl-4-bromobenzoate S7
Synthesis of Methyl-4-bromobenzoate S7
Scheme S2: Synthesis of 4-((4-Methoxyphenyl)amino)benzonitrile S8
General Procedure for the Preparation of Substituted Anisidines S8
Scheme S3: Synthesis of RAFT agent **R4** S8
General Procedure for the Preparation of RAFT Agents S8
Scheme S4: Polymerization of **PhCTC** with *N*-vinyl pyrrolidone S10
General Procedure for the Free Radical Polymerization of Vinyl Monomers with CTC S10
Scheme S5: Controlled radical polymerization of NVP and **PhCTC** S10
General Procedure for the Controlled Radical Polymerization of Vinyl Monomers with CTC S10
Scheme S6: Degradation of **PhCTC**–***co***–**PVP** by NaOMe S11
General Procedure for the Degradation of CTC Copolymers S11

X-ray Crystallography S11
Computational Details S12
Figure S1–S10: Crystallographic structure representations S13
Winkler–Dunitz Distortion Parameters S22
Table S1: Crystal data and refinement details for ***t*BuCTC**, **PhCTC**, and ***t*BuOCTC** S23
Table S2: Key crystallographic distances and angles of ***t*BuCTC**, **PhCTC**, and ***t*BuOCTC** S24
Tables S3–S4: Experimental and Calculated Winkler–Dunitz Distortion Parameters for CTCs S25
Tables S5: Absorbance maximum in 1,4-dioxane for CTCs S25
Tables S6–S7: Selected experimental ^{1}H and ^{13}C NMR shifts of CTCs S26
Figures S8–S13: Cartesian coordinates for the geometry-optimized structures of CTCs S27
Figures S11–S44: NMR spectra of CTCs and copolymers S34
Figures S45–S52: UV-Vis spectra of CTCs S51
Table S14: RAFT copolymerization of CTC and NVP with $\mathbf{R_1}$–$\mathbf{R_5}$ S55
Figures S53–S60: SEC traces of conventional and controlled CTC copolymers S56
Figures S61–S64: CTC copolymer conversion plots S60
References S62

General methods.

Solvents: CH_2Cl_2, diethyl ether, hexanes (mixture of isomers), and EtOAc were used as received without further purification.

Deuterated solvents: $CDCl_3$ (Cambridge Isotope Laboratories, Inc., D, 99.8 % + 0.03 % v/v tetramethylsilane, TMS) was used as received.

Reactants: *S*-(-)-4-amino-2-hydroxybutyric acid (Chem-Impex Int'l Inc, 99.37%), Triethylamine (VWR, ≥99.5%), 9-vinylcarbazole (Sigma-Aldrich, 98%), trimethylacetyl chloride (Alfa Aesar, ≥98%), carbon disulfide (Supelco, ≥99.9%), *N*-vinyl-2-pyrrolidone (Thermo Scientific, 99%), *N*,*N*′-thiocarbonyldiimidazole (Chem-Impex Int'l Inc, 98.37%), benzoyl chloride (TCI, >98%), acetyl chloride (Alfa Aesar, 98%), 4-methoxybenzoyl chloride (TCI, >99%), 4-(trifluoromethyl)benzoyl chloride (Oakwood Chemical, 98%), styrene (Acros Organics, 99%), ^{t}Bu acrylate (TCI, >98%), di-tert-butyl dicarbonate (Alfa Aesar, 97+%), *N*-vinyl caprolactam (TCI, >98%), 4-methylaminopyridine (Oakwood Chemical, 98%), *p*-anisidine (TCI, ≥98%), BrettPhos Pd G4 (Strem, 98%), potassium carbonate (Sigma-Aldrich, ≥99%), 4-bromobenzonitrile (TCI, >97%), 4-bromobenzoic acid (TCI, >98%), HCl (VWR, 36.5–38.0%), bromoacetonitrile (Avocado Research Chemicals, Ltd, 95%).

E. Merck silica gel (60, particle size 0.043–0.063 mm) was used for column chromatography. Melting points were determined with a MEL-TEMP II instrument using open capillaries; values are uncorrected (the heating rate was 1 °C/min). NMR measurements were recorded on a Bruker Avance III 400 and 500 spectrometer at ambient probe temperatures unless otherwise noted. $^{13}C\{^{1}H\}$ NMR resonances were obtained with proton broadband decoupling and referenced to the solvent signals of $CDCl_3$ at 77.2 (^{1}H NMR: 7.26 ($CHCl_3$)). $^{13}C\{^{1}H\}$ NMR assignments are based on DEPT 135, and the following 2D experiments: COSY, NOESY, ROESY, HSQC, and HMBC. Mass-spectrometric analyses were performed on an Agilent Technologies 1260 Infinity II/6120 Quadrupole time of flight MS system (low resolution ESI). IR spectra were measured on a Shimadzu IRAffinity-1 FT-IR Spectrometer equipped with a Universal ATR Sampling Accessory. UV/Vis spectra of solutions of **MeCTC**, **^{t}BuCTC**, **PhCTC**, **^{t}BuOCTC**, ***p*–CF_3–PhCTC**, and ***p*–MeO–PhCTC** in 1,4-dioxane or CH_2Cl_2 were measured with a Cary 5000 spectrometer. Polymer analyses were performed using a Tosoh EcoSEC HLC 8320 GPC with a TSKgel SuperHZ-L column at a 0.45 mL/min flow rate of (eluted using $CHCl_3$ containing 0.25% NEt_3). All number-average molecular weights and dispersities were calculated from refractive index chromatograms using PStQuick Mp-M polystyrene standards.

Synthesis of 3-Hydroxy-4-methoxy-4-oxobutan-1-aminium chloride (I)

Acetyl chloride (20.00 mL, 22.08 g, 281.31 mmol) was added to chilled (0 °C) MeOH (390 mL) and allowed to stir for 1 h. *S*-(-)-4-Amino-2-hydroxybutyric acid (13.40 g, 112.49 mmol) was added to the colorless solution at 0 °C and allowed to warm to room temperature. The solution was then heated to 50 °C and stirred for 4 h. After 4 h there is no observable change to the solution. The MeOH was reduced to dryness and to give a light-yellow viscous liquid. EtOAc was added to the liquid and stirred vigorously to give a white precipitate. The EtOAc was decanted and fresh EtOAc (70 mL) was added to the precipitate, vigorously stirred, and decanted. The white solid was placed under vacuum for 18 h to give a white powder. Yield: 18.51 g (109.13 mmol, 97%)

Synthesis of Methyl-2-thioxo-1,3-oxazinane-6-carboxylate (II)

N,*N′*-thiocarbonyldiimidazole (23.0507 g, 129.35 mmol, 1.0 eq) was added to a solution of **I** (21.9386 g, 129.35 mmol, 1.0 eq) in CH_2Cl_2 (410 mL). Et_3N (20.00 mL, 14.5200 g, 143.49 mmol, 1.1 eq) was immediately added dropwise to the yellow solution over a period of 3 min and stirred for 3 days at room temperature. After 3 days, the yellow solution turned a golden yellow color. The solution was reduced to dryness by rotary evaporation and then dried under belt drive pump for 1 h. The oily residue was suspended in EtOAc and purified by column chromatography over silica gel and eluted with EtOAc (100%). The product fractions were combined and the solvent was concentrated to yield a colorless crystalline solid. Yield: 7.3305 (41.84 mol, 32%); Mp: 93–94 °C. Anal. Calcd for $C_6H_9NO_3S$: C, 41.15; H, 5.18; N, 8.00; S, 18.27. Found: C, 41.14; H, 5.25; N, 7.99; S, 18.42. ^{1}H NMR (400.1 MHz, $CDCl_3$): δ 2.21–2.37 (m, 2 H; CH_2, CHC$\boldsymbol{H}_2$), 3.31–3.45 (m, 2 H; CH_2, NHC$\boldsymbol{H}_2$), 3.81 (s, 9 H; CH_3, ^{t}Bu), 4.98 (t, $^3J_{H,H}$ = 5.2 Hz, 1 H; CH), 8.75 (broad s, 1 H; NH). ^{13}C NMR (100.6 MHz, $CDCl_3$): δ 22.0 (CH_2, CH$\boldsymbol{C}H_2$), 38.6 (CH_2, NH$\boldsymbol{C}H_2$), 53.2 (CH_3, ^{t}Bu), 75.1 (CH), 168.5 (C=O), 185.5 (C=S). MS (ESI(+)): *m/z* (relative intensity) 116 (10) $[C_4H_6NOS]^+$, 176 (100) $[M + H]^+$. IR (neat, cm^{-1}): $\tilde{\nu}$ = 3211 (m, ν(N–H)), 3085 (w), 3013, 2949, 2870 (w), 2699 (w), 1740 (m, OC=O), 1556, 1543 (m) 1453, 1440 (m), 1426 (m), 1366 (m), 1355, 1320, 1316 (m), 1294 (m), 1251 (w), 1220 (m), 1204, 1181 (w), 1144 (s), 1083 (s), 1057 (m), 983 (m), 965 (m), 922 (m), 888 (w), 810 (m), 791 (m), 724 (m), 653 (m), 604 (m). UV–Vis (CH_2Cl_2): λ [nm] (ε [L · mol^{-1} · cm^{-1}]) 258, 1.58×10^4.

General Procedure for the Preparation of CTCs

II (266.21 g, 1.52 mmol) was dissolved in CH_2Cl_2 (15.0 mL) and cooled to 0 °C. Et_3N (0.32 mL, 232.32 mg, 2.28 mmol) was added to the mixture and allowed to stir for 10 minutes followed by the dropwise addition of benzyl chloride (0.21 mL, 254.10 g, 1.81 mmol). The light-yellow solution was allowed to warm to room temperature and stirred for 20 h. The solution was then washed with water (3 × 10 mL),

brine (1 × 10 mL), dried over anhydrous Na_2SO_4, filtered, and reduced to dryness. The yellow residue was subsequently purified by column chromatography over silica gel and eluted with hexanes/EtOAc (6:4 v/v).

MeCTC. This thiocarbamate was obtained as a clear yellow liquid. Yield = 58%. ^{1}H NMR (500.1 MHz, $CDCl_3$): δ 2.34–2.55 (m, 2 H; CH_2, CHC$\boldsymbol{H}_2$), 2.71 (s, 3 H; CH_3), 3.68–3.80 (m, 2 H; CH_2, NC$\boldsymbol{H}_2$), 3.81 (s, 3 H; OCH_3), 4.95 (t, $^3J_{H,H}$ = 5.7, 1 H; CH). ^{13}C NMR (125.8 MHz, $CDCl_3$): δ 25.2 (CH_2, CH$\boldsymbol{C}H_2$), 26.8 (CH_3), 41.5 (CH_2, N$\boldsymbol{C}H_2$), 53.3 (OCH_3), 75.6 (CH), 168.1 (CO_2), 174.3 (CON), 188.3 (CS). MS (ESI(+)): *m/z* (relative intensity) 176 (100) $[C_6H_8NOS + 2H]^{2+}$, 218 (30) $[M + H]^+$. IR (neat, cm^{-1}): $\tilde{\nu}$ = 2996, 2954 (w, ν(C–H)), 2920. 2851 (w) 1744 (m, (OC=O)), 1701 (m, (NC=O)), 1564 (w), 1477 (m), 1437 (m), 1418 (m), 1399 (m), 1364(m), 1312 (s), 1260 (m), 1193 (s), 1168 (s), 1082 (m), 1058 (m), 1011 (m), 992 (m), 937 (w), 916 (w), 868 (w), 778 (w). UV–Vis (Dioxane): λ [nm] (ε [L · mol^{-1} · cm^{-1}]) 282, 1.07 × 10^4.

^{t}BuCTC. This thiocarbamate was obtained by recrystallization from diethyl ether at 3 °C within 2 days as a colorless colored crystalline solid. Yield = 47%; Mp: 92–93 °C. ^{1}H NMR (500.1 MHz, $CDCl_3$): δ 1.41 (s, 9 H, ^{t}Bu), 2.38–2.47 (m, 2 H; CH_2, CHC$\boldsymbol{H}_2$), 3.44–3.49 (m, 1 H; CH_2, NC$\boldsymbol{H}_2$), 3.61–3.66 (m, 1 H; CH_2, NC$\boldsymbol{H}_2$), 3.85 (s, 3 H; OCH_3), 5.02 (t, $^3J_{H,H}$ = 4.8, 1 H; CH). ^{13}C NMR (125.8 MHz, $CDCl_3$): δ 22.8 (CH_2, CH$\boldsymbol{C}H_2$), 28.8 (CH_3, ^{t}Bu), 43.5 (CH_2, N$\boldsymbol{C}H_2$), 45.2 (C, ^{t}Bu), 53.3 (OCH_3) 75.2 (CH), 168.3 (CO_2), 182.6 (CS), 186.3 (CON). MS (ESI(+)): *m/z* (relative intensity) 260 (100) $[M + H]^+$, 176 (19) $[M + 2H]^{2+}$. IR(neat, cm^{-1}): $\tilde{\nu}$ = 2967, 2935 (w, ν(C–H)), 2905, 2872 (w), 1746 (m, ν(C=O)), 1736 (m, n (NC=O)), 1488 (m), 1477 (m), 1459 (w), 1412, 1395 (w), 1374 (m), 1365 (m), 1337 (m), 1325 (m), 1314 (m), 1298, 1278 (m), 1255 (w), 1222 (m), 1193, 1186 (s), 1128 (m), 1074 (m), 1056, 1042 (m), 1026, 1020 (m), 992 (m), 984, 956 (m), 949, 944 (m), 931 (m), 902 (w), 894 (w), 860 (w), 851 (w), 807, 800, 785 (w), 779 (w), 769, 735 (m), 717, 668, 650 (w), 624 (m), 614, 610 (w), 608. UV–Vis (Dioxane): λ [nm] (ε [L · mol^{-1} · cm^{-1}]) 242, 1.23 × 10^4; ≈277, 3.61 × 10^3 (br shoulder).

PhCTC. This thiocarbamate was obtained by recrystallization from a mixture of CH_2Cl_2 and hexanes (1:1 v/v) at room temperature within 1 day as a colorless crystalline solid. Yield: 84%; Mp: 122–123 °C. ^{1}H NMR (500.1 MHz, $CDCl_3$): δ 2.51–2.61 (m, 2 H; CH_2, CHC$\boldsymbol{H}_2$), 3.58–3.64 (m, 1 H; CH_2, NC$\boldsymbol{H}_2$), 3.90 (s, 3 H; OCH_3), 3.98–4.05 (m, 1 H; CH_2, NC$\boldsymbol{H}_2$), 5.15 (t, $^3J_{H,H}$ = 4.9, 1 H; CH), 7.43–7.46 (m, 2 H; CH, *m*-Ph), 7.53–7.57 (m, 1 H; CH, *p*-Ph), 7.91–7.93 (m, 2 H; CH, *o*-Ph). ^{13}C NMR (125.8 MHz, $CDCl_3$): δ 23.8 (CH_2, CH$\boldsymbol{C}H_2$), 42.7 (CH_2, N$\boldsymbol{C}H_2$), 53.4 (OCH_3) 75.6 (CH), 128.9 (CH, *m*-Ph), 129.7 (CH, *o*-Ph), 133.3 (C, *ipso*-Ph), 133.3 (C, *p*-Ph), 168.4 (CO_2), 172.7 (CON), 186.2 (CS). MS (ESI(+)): *m/z* (relative intensity) 105 (100) $[C_6H_5CO]^+$, 218 (91) $[M + H]^+$. IR (neat, cm^{-1}): $\tilde{\nu}$ = 3067, 3059, 2998, 2947 (w, ν(C–H)), 2873 (w), 2851 (w), 1750 (m, ν(OC=O)), 1710 (m, ν(NC=O)), 1599 (w), 1583 (w), 1492 (m), 1486 (m), 1453, 1445, 1441 (m), 1376 (m), 1365, 1328 (m), 1311 (m), 1279 (m), 1261 (w), 1240 (m), 1218

(w), 1200 (s), 1181 (s), 1159 (m), 1119 (m), 1085 (m), 1074 (m), 1036 (m), 1023 (m), 1000 (w), 987 (m), 982 (m), 960 (m), 944 (m), 926 (m), 896 (m), 871 (m), 856 (w), 844 (m), 813 (w), 789 (m), 780 (m), 726 (m), 703 (m), 691 (m), 678 (m), 645 (m), 613, 610 (w). UV–Vis (Dioxane): λ [nm] (ε [L · mol^{-1} · cm^{-1}]) 242, 1.60 × 10^4; 281, 5.42 × 10^3, ≈313, 3.62 × 10^3.

p*–CF₃–PhCTC.** This thiocarbamate was eluted with hexanes/EtOAc (4:6 v/v) followed by recrystallization from a mixture of CH_2Cl_2 and hexanes (1:1 v/v) at –3 °C within 5 days as a colorless crystalline solid. Yield: 79%; Mp: 110–111 °C. ^{1}H NMR (500.1 MHz, $CDCl_3$): δ 2.54–2.63 (m, 2 H; CH_2, CHCH***₂), 3.58–3.64 (m, 1 H; CH_2, NC***H***₂), 3.04 (s, 3 H; OCH_3), 4.06–4.12 (m, 1 H; CH_2, NC***H***₂), 5.18 (t, $^3J_{H,H}$ = 4.7, 1 H; CH), 7.70 (d, $^3J_{H,H}$ = 8.1, 2 H; CH, *m*-Ph), 8.01 (d, $^3J_{H,H}$ = 8.1, 2 H; CH, *o*-Ph). ^{13}C NMR (125.8 MHz, $CDCl_3$): δ 24.0 (CH_2, CH***C***H₂), 42.7 (CH_2, N***C***H₂), 53.5 (OCH_3) 75.7 (CH), 122.7 (CF_3), 124.8 (CF_3), 125.8–125.9 (m, CH, *m*-Ph), 129.6 (CH, *o*-Ph), 134.3 (q, C, *p*-Ph), 137.2 (C, *ipso*-Ph), 168.4 (CO_2), 171.9 (CON), 186.8 (CS). MS (ESI(+)): *m/z* (relative intensity) 173 (15) [*p*–CF_3–C_6H_4CO]$^+$, 348 (100) [M + H]$^+$. IR (neat, cm^{-1}): $\tilde{\nu}$ = 3065, 3012, 2962 (w, ν(C–H)), 2907, 2855 (w), 1942 (w), 1815 (w), 1763 (m, ν(OC=O)), 1754 (m, ν(NC=O)), 1716 (m), 1585 (w), 1510 (w), 1493 (m), 1446 (m), 1431, 1412 (m), 1381 (m), 1357 (m), 1322 (s), 1311, 1261 (m), 1221 (s), 1207 (s), 1182 (m), 1170, 1164 (m), 1125 (s), 1116 (s), 1067 (m), 1048 (m), 1013 (m), 1003 (m), 986 (m), 967 (m), 927 (m), 888 (m), 864 (m), 854 (s), 845, 779 (m), 770 (m), 760 (m), 700, 694 (m), 673 (m), 654 (m), 647 (w), 633 (w), 622 (w), 617 (w), 602 (w). UV–Vis (Dioxane): λ [nm] (ε [L · mol^{-1} · cm^{-1}]) 247, 9.68 × 10^3; 284, 5.76 × 10^3, ≈318, 3.05 × 10^3.

p*–MeO–PhCTC.** This thiocarbamate was eluted with diethyl ether/toluene (9:1 v/v) followed by recrystallization from a mixture of CH_2Cl_2 and hexanes (1:1 v/v) at –3 °C within 3 days as a colorless crystalline solid. Yield: 74%; Mp: 131–132 °C. ^{1}H NMR (500.1 MHz, $CDCl_3$): δ 2.49–2.59 (m, 2 H; CH_2, CHCH***₂), 3.52–3.58 (m, 1 H; CH_2, NC***H***₂), 3.87 (s, 3 H; *p*-CH_3O-Ph), 3.90 (s, 3 H; $COOCH_3$), 3.93–3.98 (m, 1 H; CH_2, NC***H***₂), 5.14 (t, $^3J_{H,H}$ = 4.7, 1 H; CH), 6.94 (d, $^3J_{H,H}$ = 8.8, 2 H; CH, *m*-Ph), 7.95 (d, $^3J_{H,H}$ = 8.9, 2 H; CH, *o*-Ph). ^{13}C NMR (125.8 MHz, $CDCl_3$): δ 23.6 (CH_2, CH***C***H₂), 42.7 (CH_2, N***C***H₂), 53.4 (COO***C***H₃), 55.7 (*p*-***C***H₃O-Ph), 75.4 (CH), 114.4 (CH, *m*-Ph), 124.9 (C, *ipso*-Ph), 132.7 (C, *o*-Ph), 164.3 (C, *p*-Ph), 168.6 (CO_2), 171.7 (CON), 185.4 (CS). MS (ESI(+)): *m/z* (relative intensity) 135 (100) [*p*–CH_3O–C_6H_4CO]$^+$, 310 (30) [M + H]$^+$. IR (neat, cm^{-1}): $\tilde{\nu}$ = 3002, 2957 (w, ν(C–H)), 2938 (w), 2848 (w), 1744 (m, ν(OC=O)), 1717, 1701 (m, ν(NC=O)), 1668, 1593 (m), 1564 (w), 1527 (w), 1497 (m), 1446 (m), 1438 (m), 1419, 1380 (m), 1368, 1337 (m), 1315 (m), 1301 (m), 1286 (m), 1264 (m), 1247 (m), 1215 (m), 1200 (s), 1184 (m), 1160 (s), 1111 (m), 1080, 1070 (m), 1033 (m), 1019 (m), 1002, 984 (m), 971, 959 (w), 949 (w), 927 (m), 899 (m), 874 (w), 849 (s), 819 (m), 792 (m), 778 (m), 751 (m), 718 (m), 705, 695, 690 (m), 671, 666, 655, 656, 653 (w), 633 (m), 627 (s), 616, 608 (m). UV–Vis (Dioxane): λ [nm] (ε [L · mol^{-1} · cm^{-1}]) 249, 1.47 × 10^4; 282, 1.13 × 10^4, ≈319, 5.56 × 10^3.

Synthesis of ^{t}BuOCTC.

II (254.53 mg, 1.45 mmol) was dissolved in acetonitrile (13 mL). Boc anhydride (0.40 mL, 380.00 mg, 1.74 mmol) and DMAP (17.75 mg, 0.15 mmol) were then added, and the solution was allowed to stir for 18 h at room temperature. The solvent was evaporated to dryness and the orange-colored liquid was purified by column chromatography over silica gel and eluted with hexanes/EtOAc (2:8 v/v) to give a tan colored crystalline solid after recrystallization from a mixture of CH_2Cl_2 and hexanes (1:1 v/v). Yield: 92%. Mp: 117–118 °C. ^{1}H NMR (500.1 MHz, $CDCl_3$): δ 1.54 (s, 9 H; ^{t}Bu), 2.33–2.50 (m, 2 H; CH_2, CHC***H***$_2$), 3.71 (t, $^3J_{H,H}$ = 5.0 Hz, 2 H; CH_2, NC***H***$_2$), 3.82 (s, 3 H; OCH_3), 4.89 (dd, $^3J_{H,H}$ = 6.9, 4.7 Hz, 1 H; CH). ^{13}C NMR (125.8 MHz, $CDCl_3$): δ 24.6 (CH_2, CH***C***H$_2$), 27.8 (CH_3, ^{t}Bu), 43.3 (CH_2, N***C***H$_2$), 53.2 (OCH_3), 75.4 (CH), 85.5 (C, ^{t}Bu), 152.8 (CON), 168.2 (CO_2), 185.3 (CS). MS (ESI(+)): *m/z* (relative intensity) 220 (100) [M + 2H]$^{2+}$, 276 (1) [M + H]+. IR (neat, cm-1): $\tilde{\nu}$ = 2991, 2985, 2958 (w, *ν*(C–H)), 1762 (m, (OC=O)), 1750, 1745 (m, (NC=O)), 1477 (m), 1437 (m), 1398 (m), 1374, 1370, 1363 (m), 1331 (m), 1290 (m), 1267 (w), 1247 (m), 1216 (m), 1194 (m), 1181 (m), 1150 (m), 1108 (m), 1072 (s), 1044 (m), 979 (m), 958 (m), 930 (w), 913 (w), 883 (m), 839 (m), 811 (m), 785 (m), 770 (w), 760 (m), 746 (w), 743 (w), 738 (w), 733 (w), 723 (w), 718 (w), 717 (w), 713 (w), 705, 698, 696, 687, 684, 680 (br m) 677, 674, 669, 665, 659, 655, 651 (w), 646, 638 (m), 630, 627, 625 (w), 618 (w), 611 (m), 608, 606, 603. UV–Vis (Dioxane): λ [nm] (ε [L · mol^{-1} · cm^{-1}]) 276, 1.04 × 10^4.

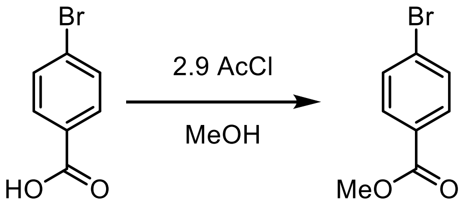

Scheme S1. Synthesis of Methyl-4-bromobenzoate.

Synthesis of Methyl-4-bromobenzoate.

Acetyl chloride (0.40 mL, 441.60 mg, 5.62 mmol) was added to a chilled (0 °C) MeOH (15 mL) and allowed to stir for 1 h. 4-bromobenzoate (382.65 mg, 1.90 mmol) was added to the colorless solution at 0 °C, allowed to warm to room temperature, and stirred for 1 h. The solution was then heated to 60 °C and stirred for 3 h. The solution was subsequently cooled to room temperature and reduced to dryness to give a white precipitate that was redissolved in CH_2Cl_2 (20 mL) and washed with 1.0 M NaOH (10 mL), water (2 × 10 mL), brine (10 mL), dried over anhydrous sodium sulfate, and filtered. The solution was then reduced to dryness to give a white precipitate. Yield: 333.70 mg (1.55 mmol, 82%). ^{1}H NMR (400.1 MHz, $CDCl_3$): δ 3.90 (s, 3 H), 7.55–7.59 (m, 2 H; *m*-CH), 7.87–7.90 (m, 2 H; CH, *o*-CH).

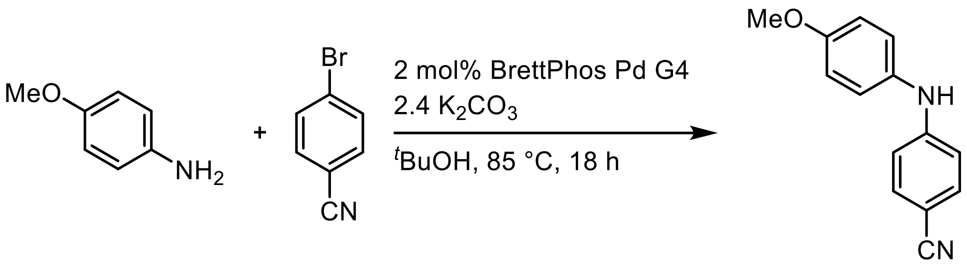

Scheme S2. Synthesis of 4-((4-Methoxyphenyl)amino)benzonitrile.

General Procedure for the Preparation of Substituted Anisidines.

This compound was prepared in analogy to a literature procedure, with modifications.[1] Under inert conditions, BrettPhos Pd G4 (36.76 mg, 0.04 mmol), K_2CO_3 (663.34 mg, 4.80 mmol), *p*-anisidine (270.48 mg, 2.20 mmol), and *p*-bromobenzonitrile (363.10 mg, 1.99 mmol) were suspended in ^{t}BuOH (10 mL) and heated to reflux for 18 h. The light-yellow solution was then cooled to room temperature and diluted with EtOAc (40 mL). The solution was washed with water (3 × 20 mL), brine (1 × 20 mL), dried over anhydrous sodium sulfate, and filtered. The light-yellow solution was then reduced to dryness. The resulting gum brown solid was purified by column chromatography over silica gel.

4-((4-methoxyphenyl)amino)benzonitrile. This anisidine was purified by column chromatography over silica gel and eluted with CH_2Cl_2/hexanes (1:1 v/v) to give a tan colored solid. Yield: 72%. ^{1}H NMR resonances were consistent with previously reported values.[2]

Methyl 4-((4-methoxyphenyl)amino)benzoate. This anisidine was purified by column chromatography over silica gel and eluted with diethyl ether/hexanes (7:3 v/v) to give a tan colored solid. Yield: 85%. ^{1}H NMR resonances were consistent with previously reported values.[2]

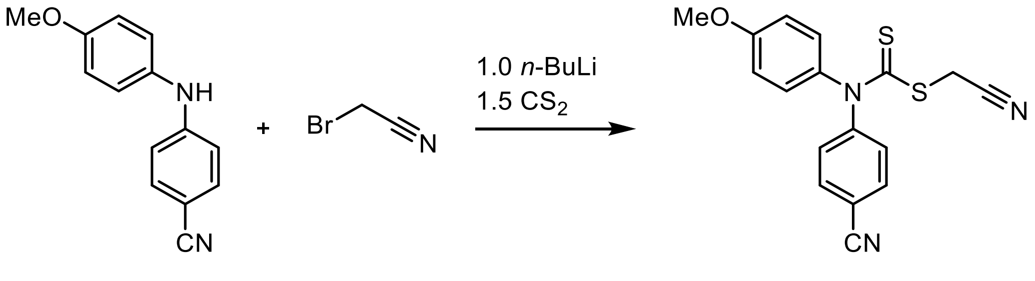

Scheme S3. Synthesis of RAFT agent **R4**.

General Procedure for the Preparation of RAFT Agents.

This compound was prepared in analogy to a literature procedure, with modifications.[3] Under inert conditions, 2.5 M *n*-Butyllithium (0.50 mL, 1.25 mmol) was added dropwise to a chilled (–10 °C) solution of 4-((4-methoxyphenyl)amino)benzonitrile (279.20 mg, 1.24 mmol) in THF (10.0 mL). The light-green solution was stirred for 1 h and then allowed to warm to 0 °C. Carbon disulfide (0.12 mL, 151.20 mg,

1.99 mmol) was added dropwise, allowed to warm to room temperature, and stirred for 18 h. Bromoacetonitrile (0.10 mL, 172.20 mg, 1.44 mmol) was then added dropwise to the chilled (0 °C) yellow solution and stirred for an additional 2 h. The solution was subsequently diluted with water (10 mL) and extracted with EtOAc (3 × 10 mL). The EtOAc fractions with combined, dried over anhydrous sodium sulfate, and reduced to dryness. The resulting gum brown liquid was purified by column chromatography over silica gel.

R4. This RAFT agent was obtained as a brown powder using diethyl ether/hexanes (7:3 %v/v) as eluent. Yield: 85%; Mp: 143–144 °C. ^{1}H NMR (500.1 MHz, $CDCl_3$): δ 3.85 (s, 3 H; CH_3), 4.06 (s, 2 H; CH_2), 6.95–6.99 (m, 2 H; *m*-CH, *p*-MeO-Ph), 7.26–7.31 (m, 2 H; *o*-CH, *p*-MeO-Ph), 7.51–7.54 (m, 2 H; *o*-CH, *p*-CN-Ph), 7.69–7.71 (m, 2 H; *m*-CH, *p*-CN-Ph). ^{13}C NMR (125.8 MHz, $CDCl_3$): δ 23.6 (CH_2), 55.8 (CH_3), 111.9 (*p*-C, *p*-CN-Ph) 115.5 (*m*-CH, *p*-MeO-Ph), 115.7 (CH_2***C***N), 118.1 (C***C***N), 128.4 (*o*-CH, *p*-CN-Ph), 129.7 (*o*-CH, *p*-MeO-Ph), 133.6 (*m*-CH, *p*-CN-Ph), 135.0 (*ipso*-C, *p*-MeO-Ph), 149.2 (*ipso*-C, *p*-CN-Ph), 160.7 (*p*-C, *p*-MeO-Ph), 197.3 (CS_2). MS (ESI(+)): *m/z* (relative intensity) 340 (100) $[M + H]^+$. IR (neat, cm^{-1}): $\tilde{\nu}$ = 3094, 3065, 3064, 3026, 2985, 2956 (w, ν(C–H_2)), 2941, 2914 (w), 2846 (w), 2233 (w, ν(C≡N)), 2230 (m, ν(C≡N)), (m), 1901 (w,) 1602 (m), 1590, 1504 (m), 1501 (w), 1463 (w), 1454 (w), 1442 (m), 1422 (w), 1408, 1368, 1338 (m), 1298, 1297, 1285, 1275, 1253 (s), 1192, 1178, 1175, 1173, 1159 (m), 1115, 1110 (m), 1043 (m), 1020 (m), 973, 962, 949, 943, 930 (m), 898 (m), 852 (m), 841 (m), 823 (m), 814, 802, 778 (m), 789 (m), 749 (m), 722 (w), 714 (m), 694 (m), 677 (w), 670 (w), 657 (w), 647 (w), 641 (w), 635 (m), 624 (w), 628 (w), 608 (w), 607 (w).

R5. This RAFT agent was obtained as a brown powder with hexanes/EtOAc (7:3 %v/v) as eluent. Yield: 88%; Mp: 144–145 °C. ^{1}H NMR (500.1 MHz, $CDCl_3$): δ 3.83 (s, 3 H; CH_3, *p*-MeO-Ph), 3.91 (s, 3 H; CH_3, COOMe), 4.07 (s, 2 H; CH_2), 6.94–6.97 (m, 2 H; *m*-CH, *p*-MeO-Ph), 7.26–7.33 (m, 2 H; *o*-CH, *p*-MeO-Ph), 7.45–7.48 (m, 2 H; *o*-CH, *p*-COOMe-Ph), 8.07–8.10 (m, 2 H; *m*-CH, *p*-COOMe-Ph). ^{13}C NMR (125.8 MHz, $CDCl_3$): δ 23.6 (CH_2), 52.5 (CH_3, *p*-COOMe-Ph), 55.8 (CH_3, *p*-MeO-Ph), 115.3 (*m*-CH, *p*-MeO-Ph) 115.9 (CH_2***C***N), 127.5 (*o*-C, *p*-COOMe-Ph), 129.5 (*o*-CH, *p*-MeO-Ph), 129.9 (*p*-C, *p*-COOMe-Ph), 131.2 (*m*-CH, *p*-COOMe-Ph), 135.9 (*ipso*-C, *p*-MeO-Ph), 149.2 (*ipso*-C, *p*-COOMe-Ph), 160.4 (*p*-C, *p*-MeO-Ph), 166.1 (CO_2), 196.9 (CS_2). MS (ESI(+)): *m/z* (relative intensity) 373 (100) $[M + H]^+$. IR (neat, cm^{-1}): $\tilde{\nu}$ = 3101, 3065, 2995, 2953 (w, ν(C–H_2)), 2932, 2903, 2835 (w), 2242 (w, ν(C≡N)), 1706 (m, ν(OC=O)), 1602 (m), 1585, 1504 (m), 1462 (w), 1455, 1441, 1430 (m), 1408 (w), 1367 (m), 1355 (m), 1287 (m), 1249 (m), 1183, 1166, 1159 (m), 1110 (m), 1098, 1043 (m), 1031 (m), 1016 (m), 987 (w), 960 (m), 930 (w), 929 (w), 903, 868 (m), 834 (m), 807 (w), 805 (w), 796 (m), 790 (m), 751 (s), 726 (m), 695 (m), 676, 666, 655 (m), 639, 630, 621 (w), 618 (w), 614 (w), 610 (w), 606 (w).

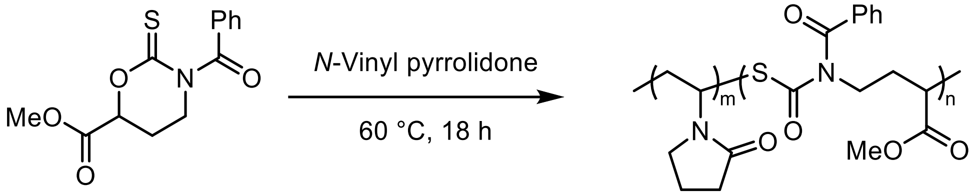

Scheme S4. Polymerization of **PhCTC** with *N*-vinyl pyrrolidone.

General Procedure for the Free Radical Polymerization of Vinyl Monomers with CTC.

N-vinylpyrrolidone (115 μL, 119.60 mg, 1.07 mmol) was added to a 4 mL vial with **PhCTC** (15.00 mg, 0.05 mmol). AIBN (3.0 mg/mL stock solution in dioxane, 150 μL, 0.45 mg, 2.6 × 10^{-3} mmol) was added to the mixture and dioxane added to obtain target concentration (4.2–6.1 M). The solution was degassed with nitrogen for 8 min, sealed with superglue, covered with electrical tape, and wrapped in parafilm. The vial was immediately placed in a preheated (60 °C) oil bath for 18 h and then allowed to cool to room temperature. The viscous solution was exposed to air, diluted with CH_2Cl_2 (1.0 mL), precipitation from diethyl ether (5.0 mL), and subjected to centrifugation (4200 rpm) for 5 min. The supernatant was then decanted, and the resulting pellet was dried under vacuum for 2 h.

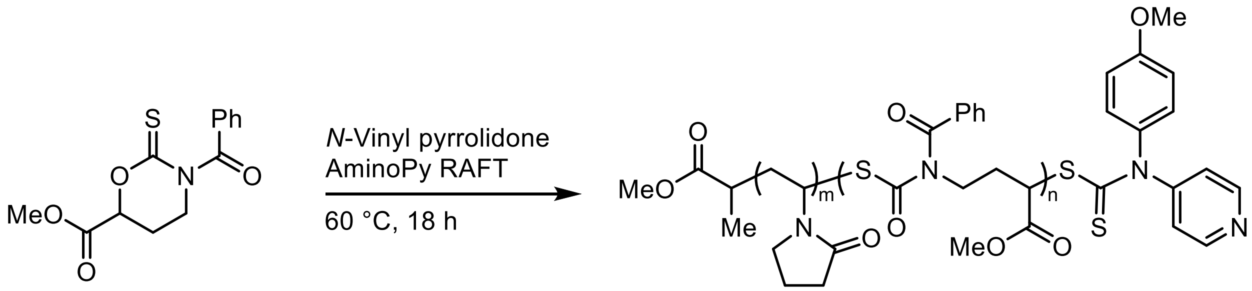

Scheme S5. Controlled radical polymerization of NVP and **PhCTC**.

General Procedure for the Controlled Radical Polymerization of Vinyl Monomers with CTC.

N-vinylpyrrolidone (689 μL, 716.56 mg, 6.45 mmol) was added to an 8 mL vial with **PhCTC** (90.02 mg, 0.32 mmol) and RAFT agent (20.36 mg, 6.45 × 10^{-2} mmol). AIBN (2.77 mg, 1.58 × 10^{-2} mmol) was added to the mixture and dioxane added to obtain a final concentration of 6.0 M. The solution was degassed with nitrogen for 8 min, sealed with superglue, covered with electrical tape, and wrapped in parafilm. The vial was immediately placed in a preheated (60 °C) oil bath for 18 h and then allowed to cool to room temperature. The solution was exposed to air, diluted with CH_2Cl_2 (1.0 mL), precipitation from diethyl ether (5.0 mL), and subjected to centrifugation (4200 rpm) for 5 min. The supernatant was then decanted, and the resulting pellet was dried under vacuum for 2 h.

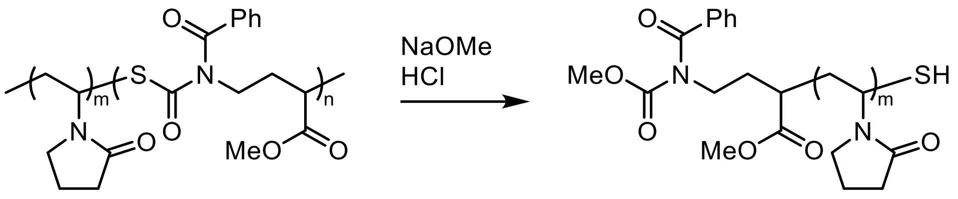

Scheme S6. Degradation of **PhCTC–*co*–PVP** by NaOMe.

General Procedure for the Degradation of CTC Copolymers.

Copolymer (15.0 mg) was dissolved in CH_2Cl_2 (1.0 mL) and NaOMe (0.25 M solution, 1.0 mL) and stirred for 18 h at room temperature. The precipitate was then treated with HCl (12.1 M) until acidic by litmus paper and subsequently neutralized with a saturated solution of NaHCO. The mixture was diluted with water (2.0 mL), extracted with CH_2Cl_2 (3.0 mL), and dried over anhydrous Na_2SO_4. The CH_2Cl_2 solution was then reduced to dryness to give the degraded polymer residue.

X-ray Crystallography.

Single crystals of ***ᵗ*BuCTC** were obtained as colorless triangular prisms from a solution diethyl ether and stored at 3 °C. Rectangular plates (**PhCTC**) and prisms (***ᵗ*BuOCTC**) were obtained by allowing hexane vapor to slowly diffuse into a CH_2Cl_2 solution at room temperature. X-ray data for ***ᵗ*BuCTC**, **PhCTC**, ***ᵗ*BuOCTC** were collected on a Bruker D8 Venture diffractometer (MoKa radiation, λ = 0.71073 Å) by using ω and *φ* scans at 100(1) K (Table S1). Absorption correction and scaling was performed using SADABS[4] as implemented in APEX4. The integrated intensity for each reflection was obtained by reduction of the data frames using SAINT V8.B40[5] in APEX4. Cell parameters were obtained and refined with 18367 (3180 unique, ***ᵗ*BuCTC**), 22981 (3661 unique, **PhCTC**), and 22119 (4128 unique, ***ᵗ*BuOCTC**) reflections, respectively. The structures were solved with the ShelXT structure solution program using the Intrinsic Phasing solution method and refined by full-matrix least-squares methods against F^2 using SHELXL-2014[6] and Olex2 1.5[7] as the graphical interface. The hydrogen atoms were placed in idealized positions and refined by using a riding model. Non-hydrogen atoms were refined with anisotropic thermal parameters. Absolute structure determination was determined by anomalous dispersion. For ***ᵗ*BuCTC**, elongated thermal ellipsoids on the C2, C3, C5, C7, C11, N1, O2, and O4 atoms indicated disorder, which was modeled successfully between two positions each with an occupancy ratio of 0.75:0.25. In modelling disorder in the structure of ***ᵗ*BuCTC,** the C8-C11B distance was restrained using DFIX (input = 1.55 0.005). Without restraining the distance, artificially long (>1.75 Å) C8-C11B distances were obtained due to incorporation of residual density around O4B in part 2 of the disorder model.

Deposition Number(s) CCDC 2311623 (for ***ᵗ*BuCTC**), 2311622 (for **PhCTC**), and 2311621 (for ***ᵗ*BuOCTC**) contain(s) the supplementary crystallographic data for this paper. These data are provided free

of charge by the joint Cambridge Crystallographic Data Centre and Fachinformationszentrum Karlsruhe Access Structures service www.ccdc.cam.ac.uk/structures.

Computational Details.

All structures were fully geometry-optimized using Gaussian 09 software package.[8] Density functional theory was used with the B3LYP functional and the 6-31G* basis set.[9-11] The solvation corrections in CH_2Cl_2 were performed with the SMD solvation model. No symmetry constraints were imposed on any structures during optimization. The energetic minimum of the calculated structures was confirmed by vibration analyses performed analytically. To obtain higher-precision free energies, single-point energy calculations were performed using the M06-2X functional and the def2-TZVPP basis set.[12-13] All of the energies reported are free energies in the unit of kcal/mol.

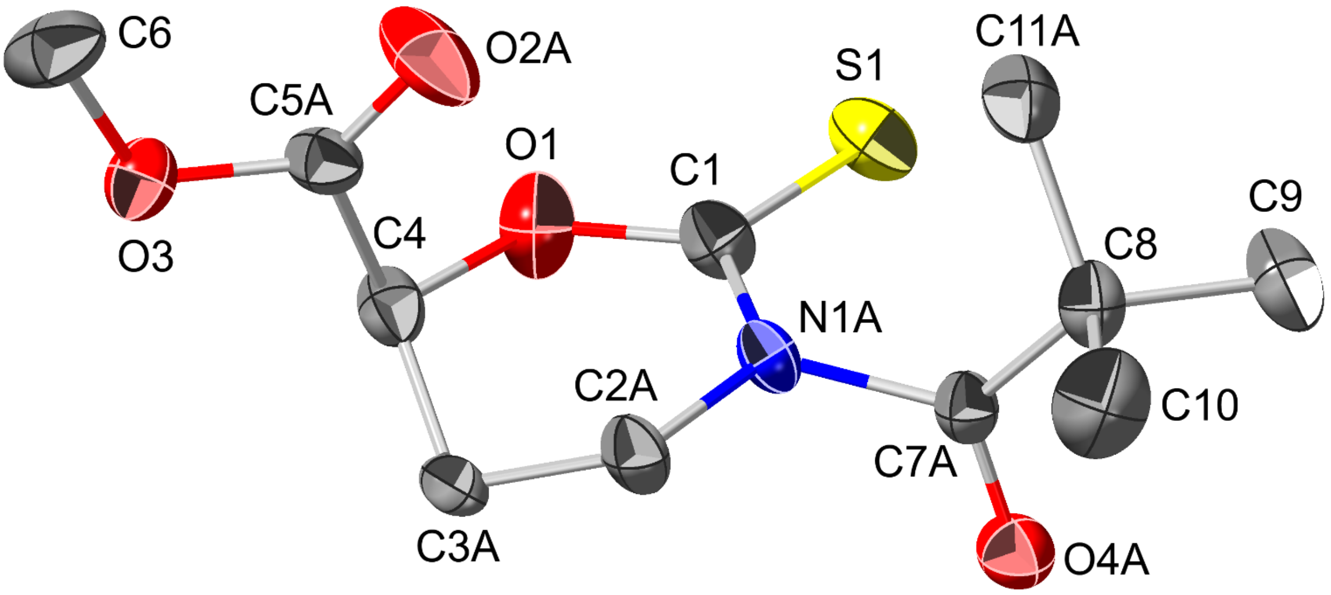

Figure S1. Molecular structure of **^{t}BuCTC** showing ellipsoids with anisotropic displacement factors of 50% probability. Atoms C2, C3, C5, C7, C11, O2, O4, and N1 indicated disorder and were modeled between two positions each (occupancy 0.75:0.25). The dominant conformation is depicted. Hydrogen atoms have been omitted for clarity. Selected interatomic distances (Å), bond angles (deg), and torsion angles (deg): S1–C1 1.664(2), O1–C1 1.325(3), O1–C4 1.446(3), N1A–C1 1.335(4), N1A–C2A 1.476(4), N1A–C7A 1.458(4), O4A–C7A 1.196(5), C8–C7A 1.490(4), C1–O1–C4 122.6(2), O1–C1–S1 118.15(19), N1A–C1–S1 122.1(2), O1–C1–N1A 118.9(2), C1–N1A–C2A 123.9(2), N1A–C7A–O4A 117.9(3), S1–C1–N1A–C7 –4.9(4), C1–N1A–C7–C8 96.3(4), C1–N1A–C7–O4 –96.3(4), C1–N1A–C2–C3 28.9(5), C1–O1–C4–C3A –34.1(4), O1–C1–N1A–C7A 164.4(3).

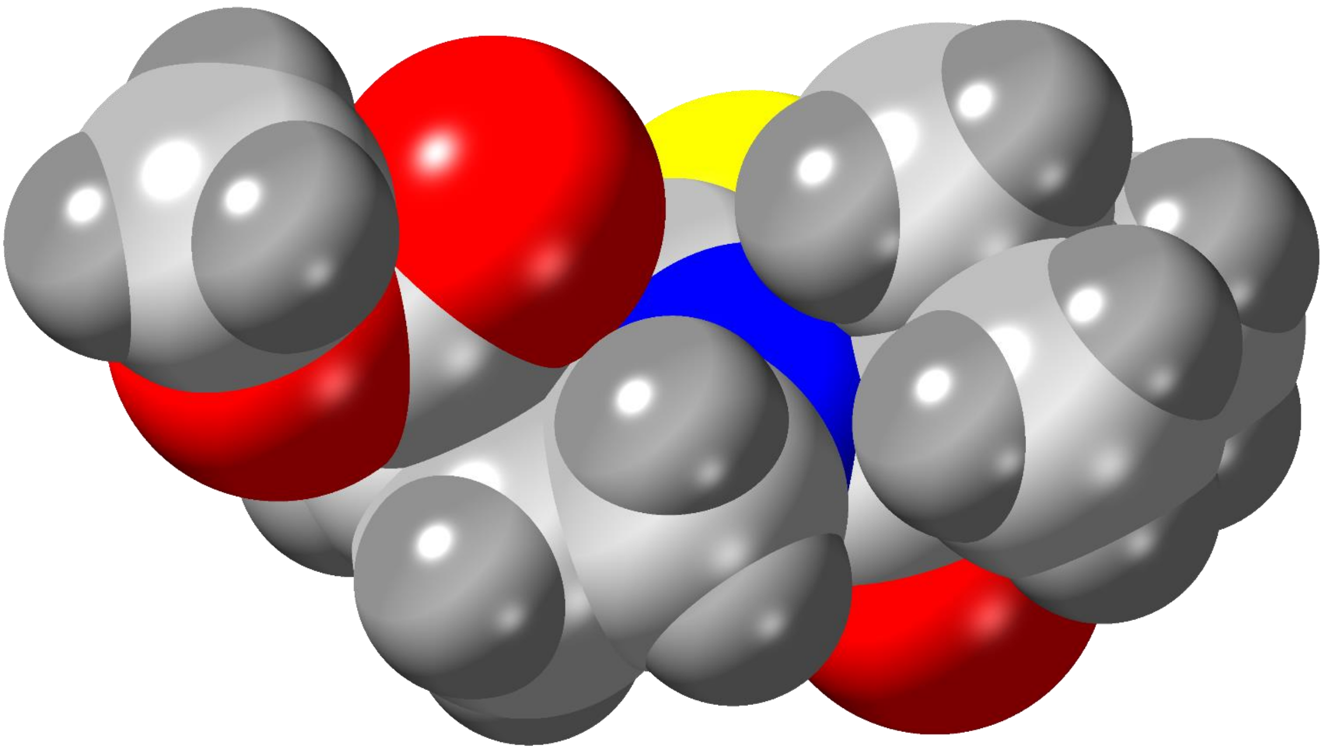

Figure S2. Space filling representation of the molecular structure (dominant conformation) of ***t*BuCTC**.

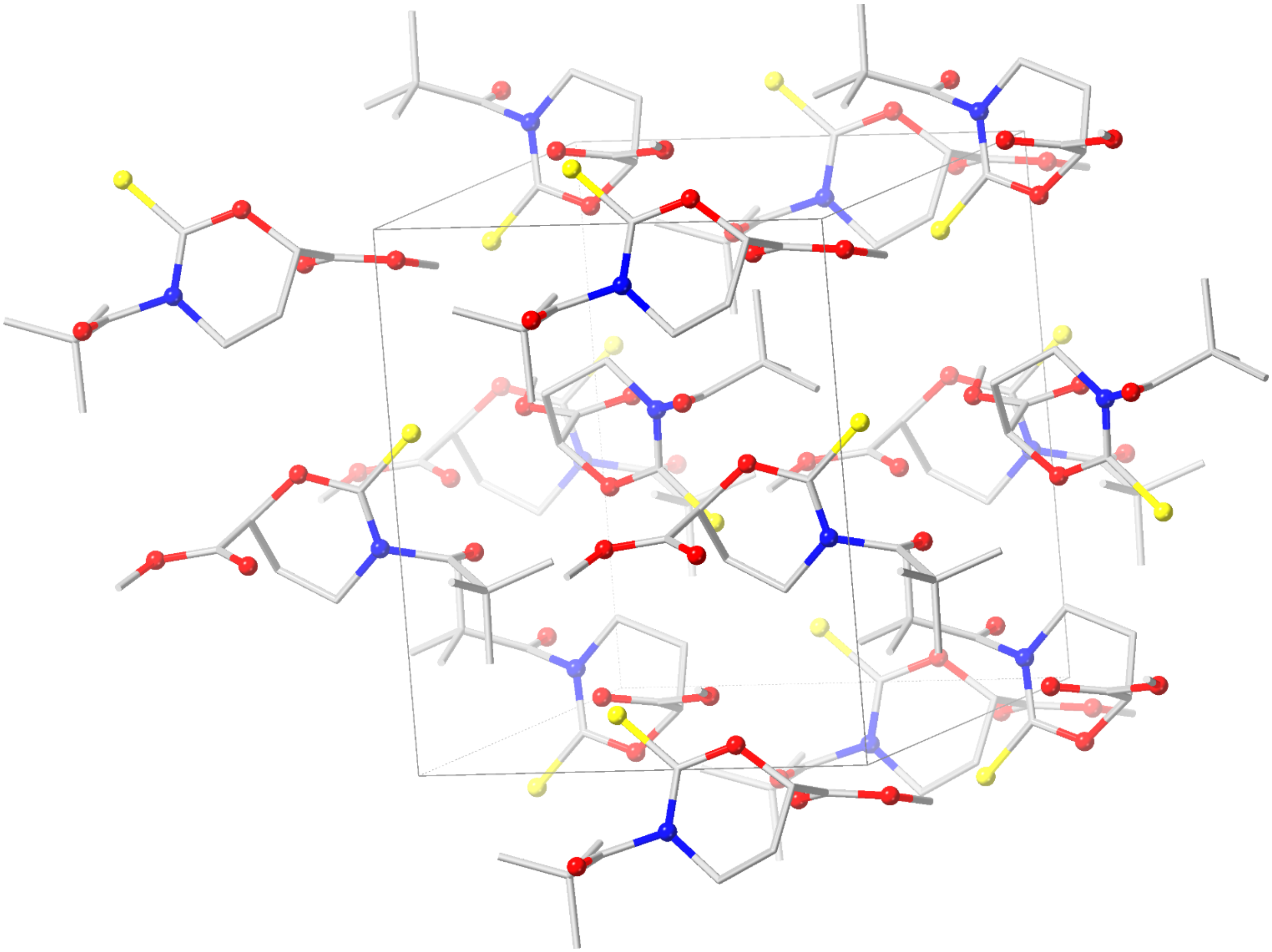

Figure S3. Crystal packing diagram of the unit cell of ^{t}**BuCTC**. Dominant conformation depicted.

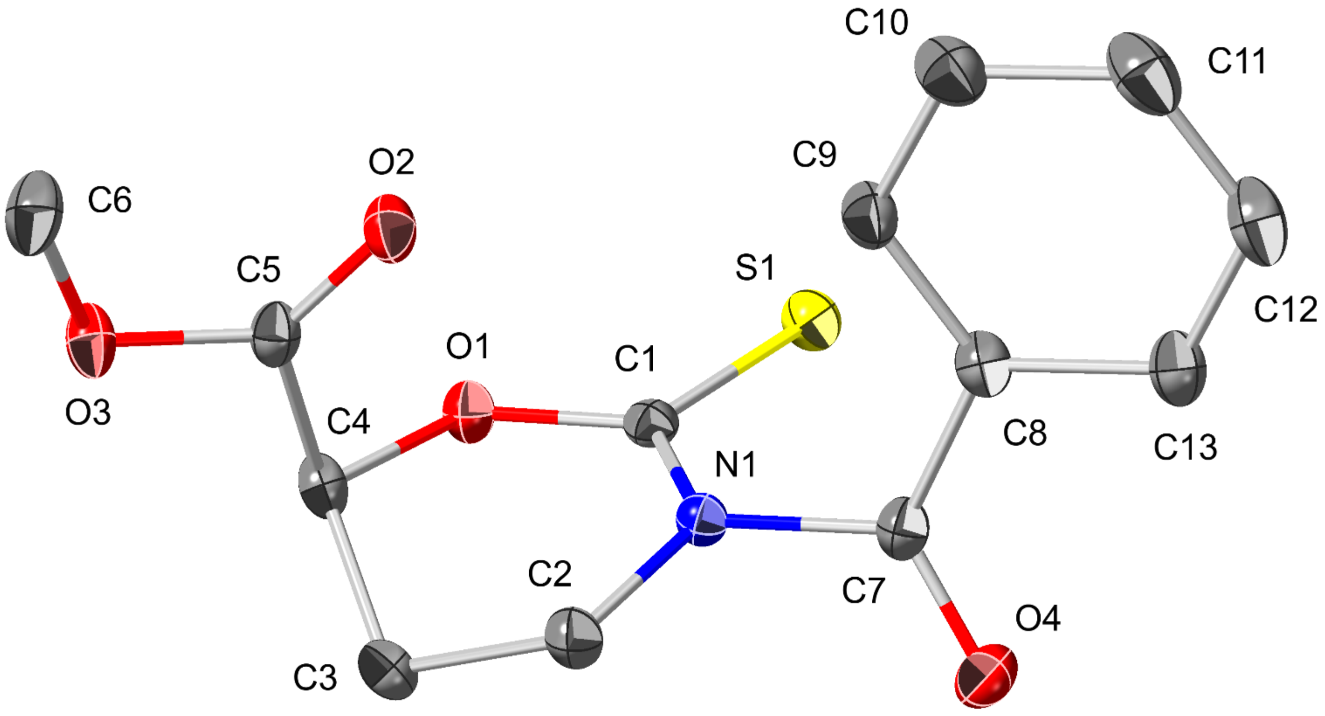

Figure S4. Molecular structure of **PhCTC** showing ellipsoids with anisotropic displacement factors of 50% probability. Hydrogen atoms have been omitted for clarity. Selected interatomic distances (Å), bond angles (deg), and torsion angles (deg): S1–C1 1.6724(18), O1–C1 1.331(2), O1–C4 1.447(2), N1–C1 1.336(2), N1–C2 1.478(2), N1–C7 1.460(2), O4–C7 1.205(2), C8–C7 1.480(2), C1–O1–C4 120.16(14), O1–C1–S1 117.82(13), N1–C1–S1 122.07(14), O1–C1–N1 120.08(16), C1–N1–C2 125.45(15), N1–C7–O4 117.95(15), S1–C1–N1–C7 2.6(2), C1–N1–C7–C8 81.7(2), C1–N1–C7–O4 –105.8(2), C1–N1–C2–C3 12.5(2), C1–O1–C4–C3 –38.8(2), O1–C1–N1–C7 –179.52(14).

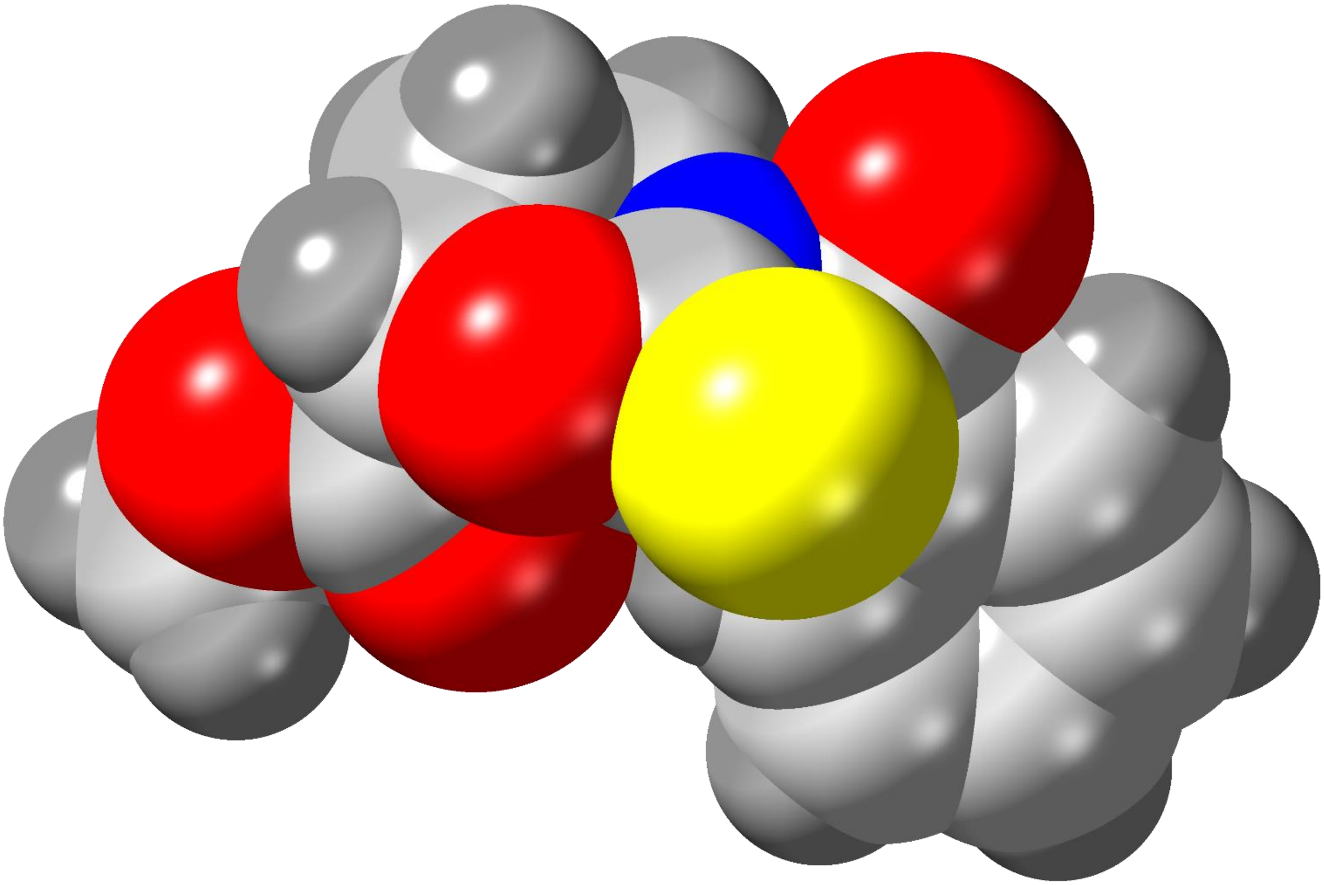

Figure S5. Space filling representation of the molecular structure of **PhCTC**.

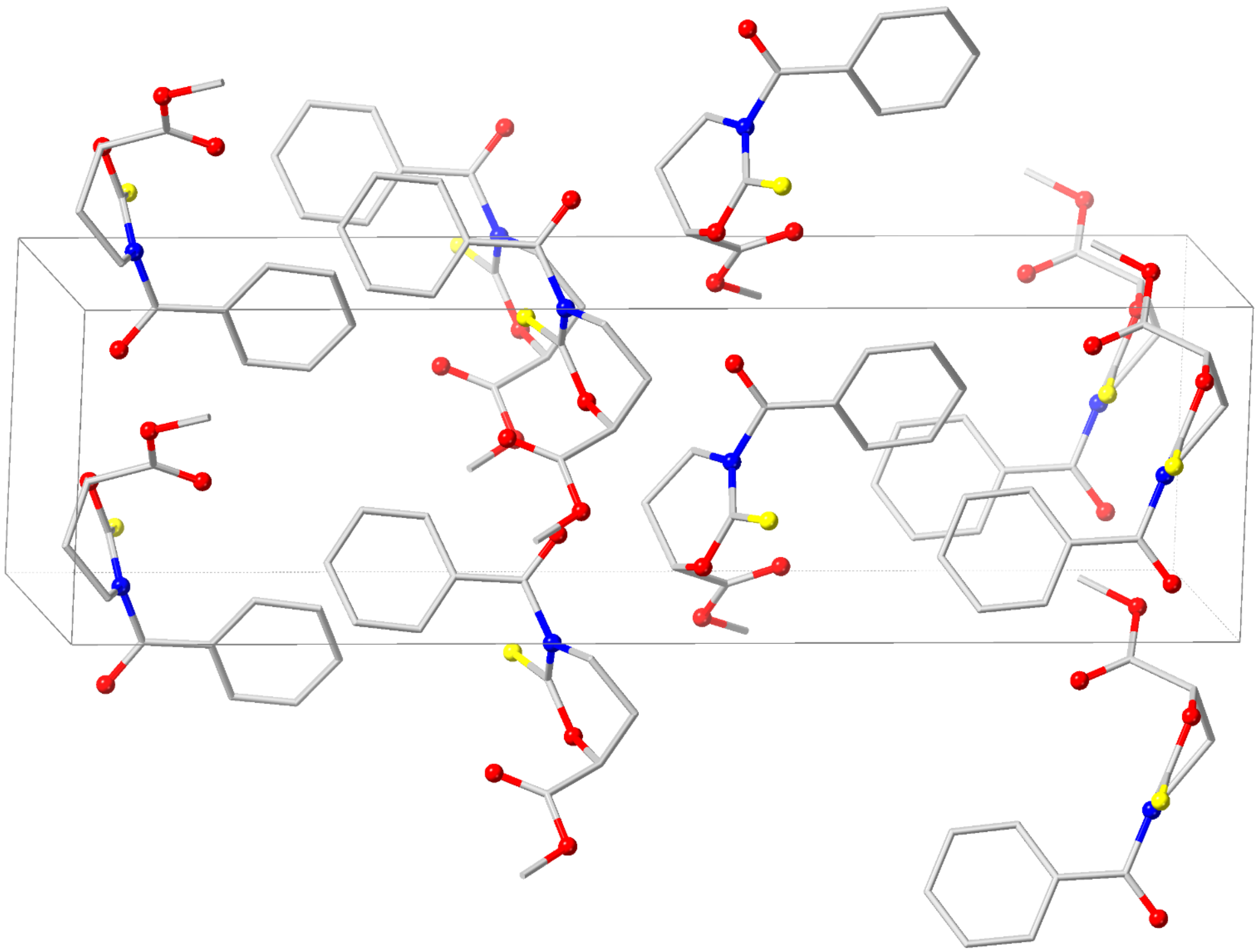

Figure S6. Crystal packing diagram of the unit cell of **PhCTC**. Hydrogen have been omitted for clarity.

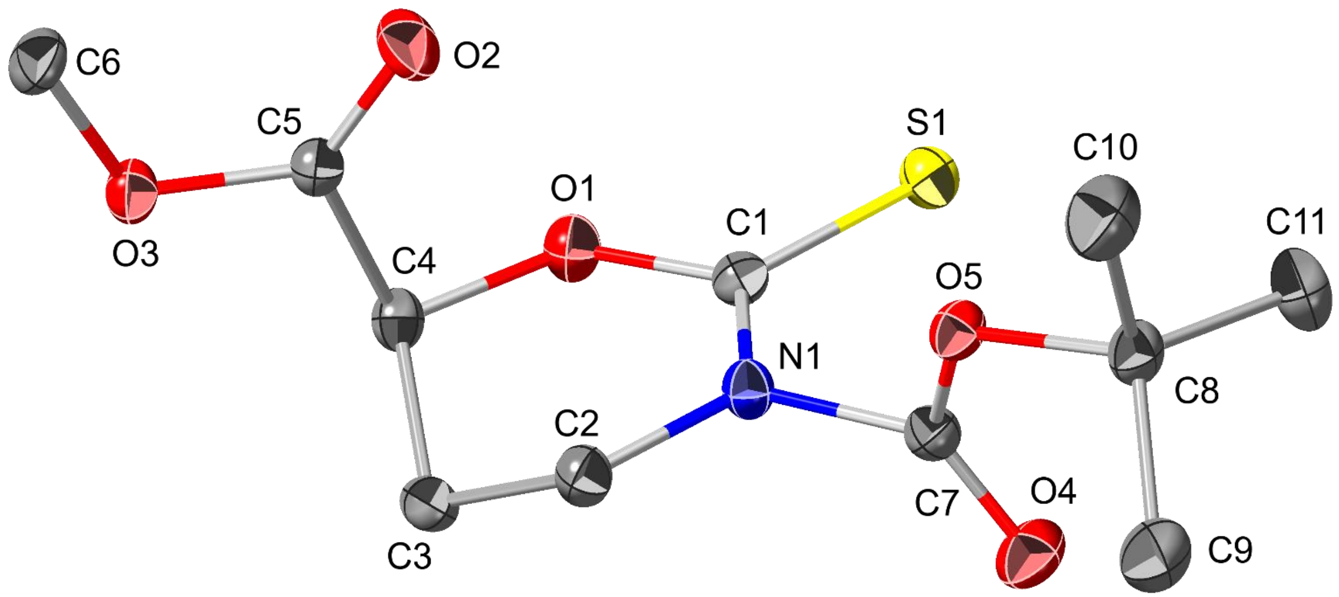

Figure S7. Molecular structure of ***t*BuOCTC** showing ellipsoids with anisotropic displacement factors of 50% probability. Hydrogen atoms have been omitted for clarity. Selected interatomic distances (Å), bond angles (deg), and torsion angles (deg): S1–C1 1.6661(19), O1–C1 1.343(2), O1–C4 1.452(2), N1–C1 1.337(2), N1–C2 1.480(2), N1–C7 1.453(2), O4–C7 1.193(2), O5–C7 1.316(2), C1–O1–C4 121.83(15), O1–C1–S1 117.76(14), N1–C1–S1 123.28(14), O1–C1–N1 118.96(16), C1–N1–C2 125.20(16), N1–C7–O4 121.88(18), S1–C1–N1–C7 –3.9(2), C1–N1–C7–O5 103.73(19), C1–N1–C7–O4 –81.1(2), C1–N1–C2–C3 27.9(2), C1–O1–C4–C3 –31.6(2), O1–C1–N1–C7 175.24(16).

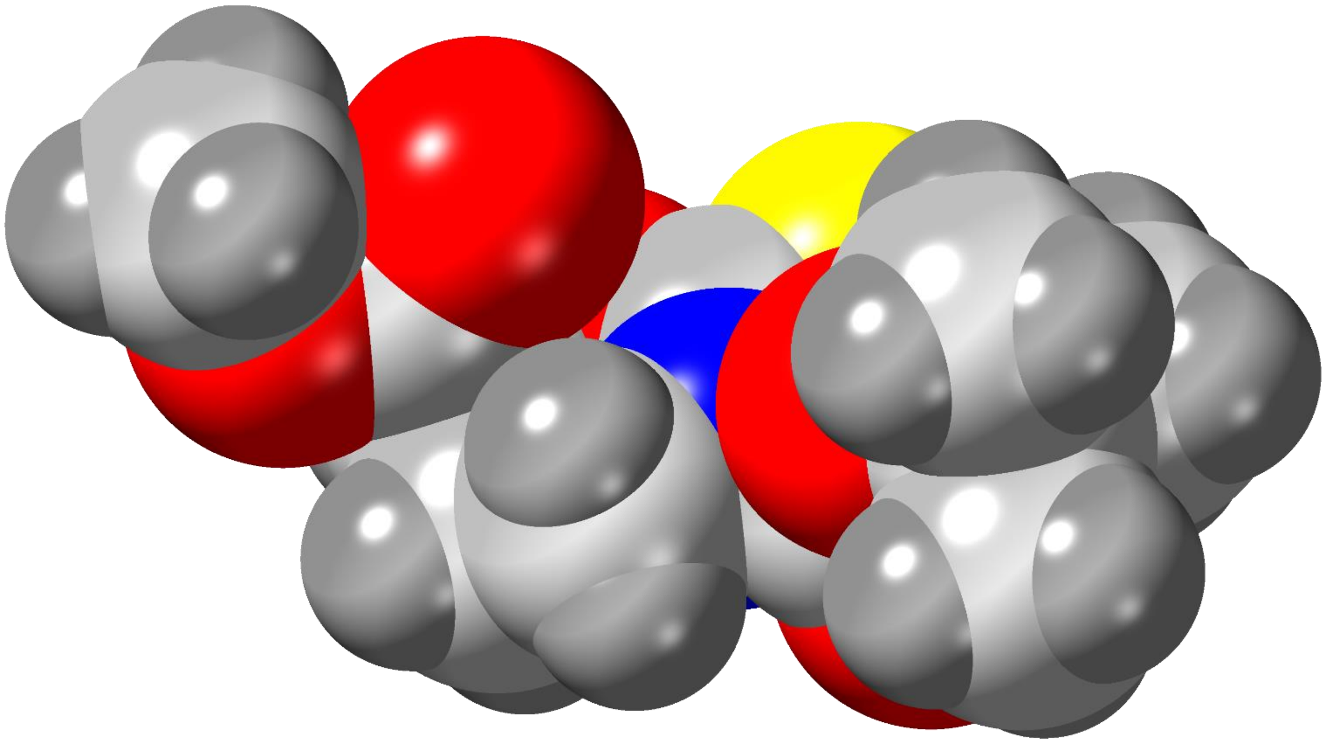

Figure S8. Space filling representation of the molecular structure of ***t*BuOCTC**.

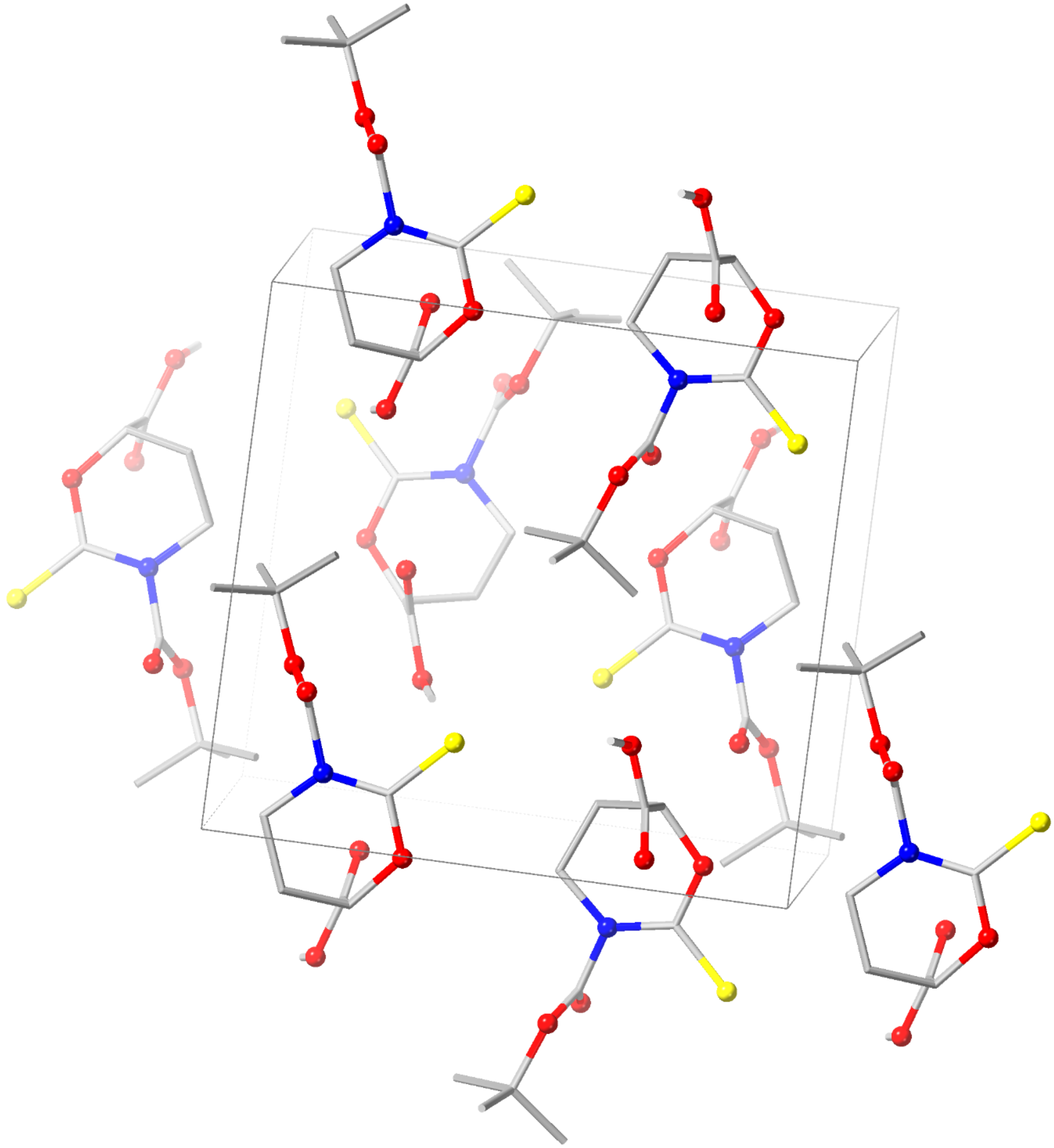

Figure S9. Crystal packing diagram of the unit cell of ***t*BuOCTC**. Hydrogen have been omitted for clarity.

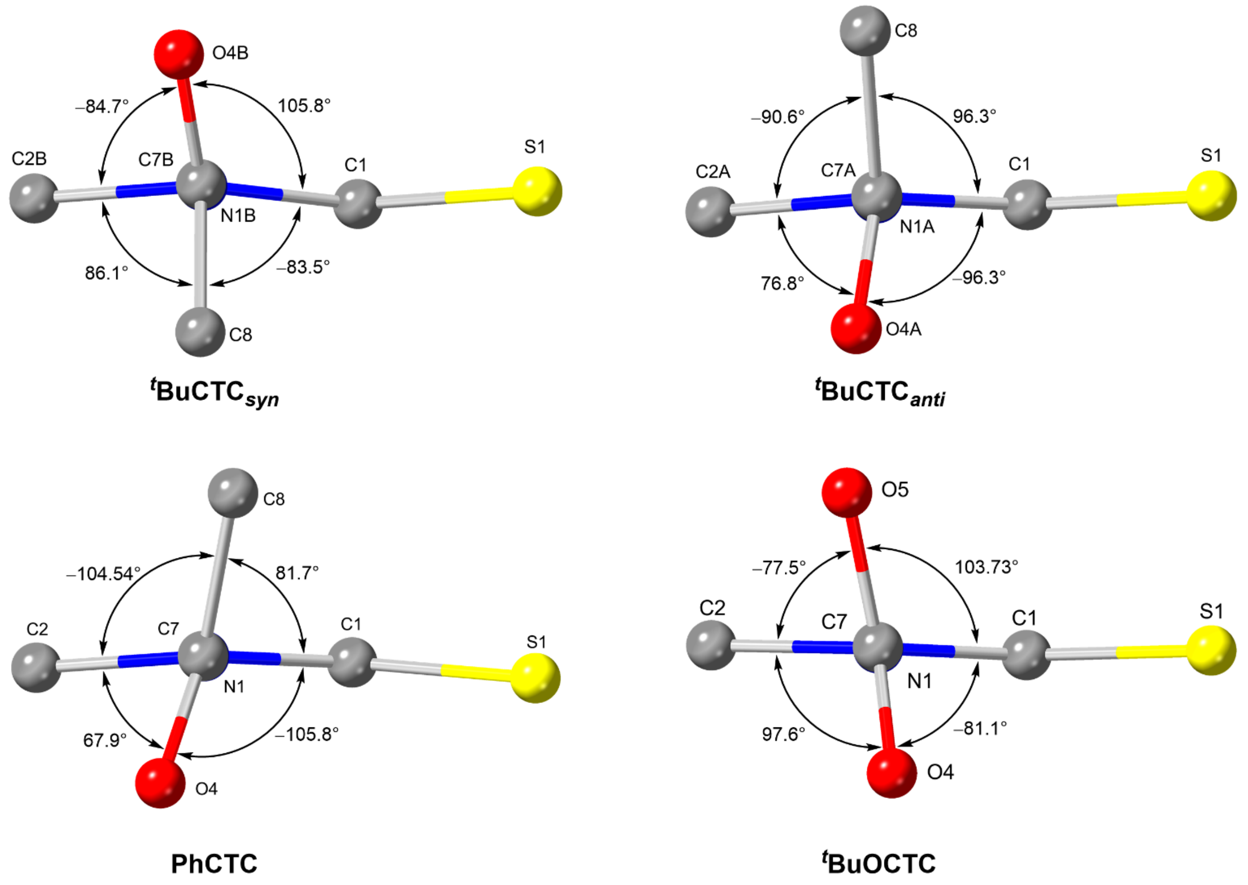

Figure S10. Newman projects of CTCs with torsion angles between acyl and carbamate moieties. Hydrogen atoms, C3, C4, C5, C6, O2, and O3 have been omitted for clarity. C9, C10, C11A, and C11B have been omitted for clarity for ***ᵗ*BuCTC**. C9, C10, C11, C12, and C13 have been omitted for clarity for **PhCTC**. C8, C9, C10, and C11 have been omitted for clarity for ***ᵗ*BuOCTC**.

Winkler–Dunitz Distortion Parameters

While currently speculative and limited by data points, the amide twist angle may provide insight into reactivity. The Winkler–Dunitz distortion parameters[2] indicate that the amide bonds are highly twisted (τ = 75–87°) with near planar nitrogen atoms (χN = 1–10°) for ***ᵗ*BuCTC*anti***, ***ᵗ*BuCTC*syn***, **PhCTC**, and ***ᵗ*BuOCTC** indicating a lack of orbital overlap for delocalization to occur between the carbamate and acyl moieties in the solid-state. These values are within range for experimentally obtained parameters of thiocarbonyl-containing twisted amides (Figure S10, Tables S3–S4).[3-6] The N(1)–C(7) bonding distances between 1.458(4)–1.468(14) Å further support the independence of each group by having single bond character. The relative stability (kcal/mol) of the molecular structures of the CTCs (*syn* and *anti*-positions of carbonyl isomers) was modeled in CH_2Cl_2 at the M06-2X/def2-TZVPP level of theory. All *anti* isomers for the CTCs are energetically favored by 0.74–2.40 kcal/mol. Computationally optimized molecular structures of ***ᵗ*BuCTC** and **PhCTC** have twist angles of 80° and 66°, respectively. Notably, the less sterically encumbered **MeCTC** and ***ᵗ*BuOCTC** have lowest twist angle values of 19° and 33°, respectively. These results are in agreement with UV-Vis spectroscopy data that qualitatively follows the same trend whereby the decreasing twist angle shows an increasing bathochromic shift when compared to the absorption maximum of **II** (Figure S45–S52). Conversely, the absorption maxima of the sterically encumbered **PhCTC** and ***ᵗ*BuCTC** have a hypsochromic shift when compared to **II**.

Table S1. Crystal data and refinement details for ***t*****BuCTC**, **PhCTC**, and ***t*****BuOCTC**.

	t**BuCTC**	**PhCTC**	***t*****BuOCTC**
Empirical formula	$C_{11}H_{17}NO_4S$	$C_{13}H_{13}NO_4S$	$C_{11}H_{17}NO_5S$
M_r	259.31	279.30	275.31
Crystal size [mm]	0.255 × 0.252 × 0.09	0.434 × 0.315 × 0.03	0.32 × 0.2 × 0.19
Crystal system	orthorhombic	orthorhombic	orthorhombic
Space group	$P2_12_12_1$	$P2_12_12_1$	$P2_12_12_1$
a [Å], α [°]	10.0236(15), 90	7.2081(6), 90	10.7412(16), 90
b [Å], β [°]	11.1878(17), 90	7.2498(6), 90	10.8464(15), 90
c [Å], γ [°]	11.4279(17), 90	25.110(2), 90	11.5731(18), 90
V [Å^3]	1281.5(3)	1312.19(19)	1348.3(3)
Z	4	4	4
$\rho_{calcd.}$ [g cm^{-3}]	1.344	1.414	1.356
$F(000)$	552	584	584
μ [mm^{-1}]	0.256	0.256	0.253
T_{max}/T_{min}	0.746 / 0.661	0.746 / 0.653	0.734 / 0.669
hkl range	±13, ±14, ±15	–9 +10, ±10, ±34	±15, ±15, ±16
θ range [°]	5.096 – 56.614	5.848 – 59.148	5.148 – 61.014
Measured refl.	18367	22981	22119
Unique refl. [R_{int}]	3180 [0.0541]	3661 [0.0502]	4128 [0.0635]
Data / restr. / param.	3180 / 12 / 231	3661 / 0 / 173	4128 / 0 / 167
Goodness-of-fit	1.080	1.085	1.042
$R1$ (I>2σ(I))	0.0376	0.0300	0.0342
$wR2$ (I>2σ(I))	0.0897	0.0719	0.0799
$R1$ (all data)	0.0427	0.0335	0.0400
$wR2$ (all data)	0.0939	0.0748	0.0836
Flack parameter	0.00(4)	0.03(3)	0.06(4)
Resid. electron dens. [e Å^{-3}]	0.26 / –0.38	0.24 / –0.25	0.24 / –0.25

Table S2. Key crystallographic distances and angles of ***t*BuCTC**, **PhCTC**, and ***t*BuOCTC**.

	***t*BuCTC**	**PhCTC**	***t*BuOCTC**
	bond/angle [Å]/[°]		
C=S	1.664(2)	1.6724(18)	1.6661(19)
O–$C_{Thiocarbamate}$	1.325(3)	1.331(2)	1.343(2)
N–$C_{Thiocarbamate}$	1.335(4)<br>1.417(9)*	1.336(2)	1.337(2)
N–C_{Amide}	1.458(4)<br>1.468(14)*	1.460(2)	1.453(2)
C_{Amide}=O	1.196(5)<br>1.197(12)*	1.205(2)	1.193(2)
O–C=S	118.15(19)	117.82(13)	117.76(14)
N–C=S	122.1(2)<br>120.6(4)*	122.07(14)	123.28(14)
O–$C_{Thiocarbamate}$–N	118.9(2)<br>111.9(4)*	120.08(16)	118.96(16)
N–C=O	117.9(3)<br>119.0(10)*	117.95(15)	121.88(18)
S=C–N–C_{Amide}	–4.9(4)<br>–10.1(10)*	2.6(2)	–3.9(2)
SC–N–C=O	−96.3(4)<br>105.8(11)*	−105.8(2)	–81.1(2)
SC–N–CH_2–CH_2	28.9(5)<br>6.4(13)*	12.5(2)	27.9(2)
O–C–N–C_{Amide}	164.4(3)<br>–156.2(7)*	−179.52(14)	175.24(16)

*) Bond distances and angles for ***t*BuCTC*syn***.

Table S3. Experimental Winkler–Dunitz distortion parameters.

	^{t}**BuCTC**	**PhCTC**	^{t}**BuOCTC**
	angle [°]		
τ	87<br>84*	75	79
χ_N	7<br>10*	6	1
χ_C	13<br>9*	8	5
$\tau + \chi_N$	93<br>94*	81	81

*) Winkler–Dunitz distortion parameters for ^{t}**BuCTC**$_{syn}$.

Table S4. Calculated Winkler–Dunitz distortion parameters.

	**MeCTC**	^{t}**BuCTC**	**PhCTC**	^{t}**BuOCTC**	***p*–CF$_3$–PhCTC**	***p*–MeO–PhCTC**
	angle [°]					
τ	19	80	66	33	61	70
χ_N	3	12	9	4	10	9
χ_C	6	12	7	5	8	7
$\tau + \chi_N$	22	92	75	37	71	79

Table S5. Absorbance maximum in 1,4-dioxane for CTCs.

**MeCTC**	^{t}**BuCTC**	**PhCTC**	^{t}**BuOCTC**	***p*–CF$_3$–PhCTC**	***p*–MeO–PhCTC**
[nm]					
282	242,<br>277*	242,<br>281<br>313*	276	247,<br>284,<br>318*	249,<br>282,<br>319*

*) Broad shoulder

Table S6. Selected experimental ^{1}H NMR shifts of CTCs.

	**MeCTC**	**^{t}BuCTC**	**PhCTC**	**^{t}BuOCTC**	***p*–CF$_3$–PhCTC**	***p*–MeO–PhCTC**
			δ ($CDCl_3$) [ppm]			
CHC***H***$_2$	2.34–2.55	2.38–2.47	2.51–2.61	2.33–2.50	2.54–2.63	2.49–2.59
NCH$_2$	3.68–3.80	3.44–3.49, 3.61–3.66	3.58–3.64, 3.98–4.05	3.70–3.72	3.58–3.64 4.06–4.212	3.52–3.58 3.93–3.98
COOCH$_3$	3.81	3.85	3.90	3.82	3.04	3.90
CH	4.94–4.96	5.01–5.03	5.14–5.16	4.88–4.90	5.17–5.19	5.13–5.15

Table S7. Selected experimental ^{13}C NMR shifts of CTCs.

	**MeCTC**	**^{t}BuCTC**	**PhCTC**	**^{t}BuOCTC**	***p*–CF$_3$–PhCTC**	***p*–MeO–PhCTC**
			δ ($CDCl_3$) [ppm]			
CH***C***H$_2$	25.2	22.8	23.8	24.6	24.0	23.6
NCH$_2$	41.5	43.5	42.7	43.3	42.7	42.7
COO***C***H$_3$	53.3	53.3	53.4	53.2	53.5	53.4
CH	75.6	75.2	75.6	75.4	75.7	75.4
CO$_2$	168.1	168.3	168.4	168.2	168.4	168.6
CON	174.3	186.3	172.7	152.8	171.9	171.7
CS	188.3	182.6	186.2	185.3	186.8	185.4

Table S8. Cartesian coordinates for the geometry-optimized structure of **MeCTC** (B3LYP/6-31G*).

S	2.4321	–1.726184	–0.444551
O	–3.170098	0.064349	–0.023785
O	–0.034571	–1.663368	0.257078
O	1.89451	2.483463	0.58474
O	–1.460128	–0.106276	–1.500099
N	1.004579	0.405353	0.462531
C	–1.947483	–0.316879	–0.411505
C	1.844719	1.440146	–0.043969
C	1.074818	–0.923617	0.100769
C	–1.256146	–1.068283	0.731794
H	–1.882909	–1.916801	1.01444
C	0.051107	0.889024	1.507189
H	–0.485098	1.741284	1.081897
H	0.636728	1.254829	2.352917
C	–0.916129	–0.203539	1.938015
H	–0.470869	–0.854435	2.696869
H	–1.815837	0.246806	2.364272
C	–3.953563	0.784838	–1.004212
H	–3.4401	1.702841	–1.299953
H	–4.895327	1.016611	–0.506908
H	–4.128316	0.158156	–1.882176
C	2.529924	1.293324	–1.379899
H	3.542194	0.901089	–1.252601
H	2.599969	2.300034	–1.80119
H	1.992736	0.635625	–2.065034

Table S9. Cartesian coordinates for the geometry-optimized structure of ***t*BuCTC** (B3LYP/6-31G*).

S	–1.26913	2.257583	–1.06964
O	3.848843	–0.5513	0.073086

O	1.133227	1.740781	–0.23261
O	–2.55952	0.382193	1.663245
O	2.053803	–0.62025	–1.30583
N	–0.50624	0.341381	0.622488
C	2.657429	–0.1371	–0.37234
C	–1.9421	–0.01648	0.704979
C	–0.15521	1.366608	–0.16927
C	2.155581	1.024523	0.489888
H	2.965397	1.746195	0.612879
C	0.378741	–0.28676	1.630259
H	0.645259	–1.29315	1.288793
H	–0.1919	–0.37961	2.557614
C	1.617572	0.570555	1.844252
H	1.372703	1.461432	2.430989
H	2.37915	0.002612	2.384772
C	4.435804	–1.67403	–0.62589
H	3.784204	–2.54841	–0.55471
H	5.38455	–1.8625	–0.12362
H	4.602024	–1.42014	–1.67555
C	–2.48309	–1.07485	–0.26662
C	–2.4343	–2.40099	0.545081
H	–2.8579	–3.20693	–0.06445
H	–1.40617	–2.68056	0.803097
H	–3.01575	–2.32236	1.469285
C	–3.94841	–0.73902	–0.60003
H	–4.38321	–1.54849	–1.19699
H	–4.5462	–0.62134	0.308578
H	–4.0157	0.188246	–1.17883
C	–1.66286	–1.25124	–1.55292
H	–1.71733	–0.36605	–2.19133

Table S10. Cartesian coordinates for the geometry-optimized structure of **PhCTC** (B3LYP/6-31G*).

S	–0.73063	–1.8383	1.938068
O	1.658008	–1.26159	1.11603
O	1.852092	1.452654	0.658041
O	3.905606	1.147775	–0.24249
O	–1.77495	–1.8669	–1.67047
N	0.013497	–1.0138	–0.49961
C	–2.24053	0.105681	–0.41601
C	–1.40061	–1.02212	–0.88354
C	2.354955	–1.32803	–1.20785
H	2.406877	–2.42084	–1.17508
H	3.099719	–0.97135	–1.92406
C	0.967639	–0.86713	–1.62519
H	0.972978	0.183138	–1.93805
H	0.590504	–1.47088	–2.4536
C	2.661599	–0.79852	0.190254
H	3.598614	–1.21047	0.569054
C	–3.62166	0.042006	–0.66804
H	–4.0319	–0.84257	–1.14426
C	–1.69435	1.248132	0.191905
H	–0.62779	1.310816	0.382553
C	–2.52592	2.309675	0.543893
H	–2.10029	3.193845	1.009876
C	–3.90051	2.237101	0.301719
H	–4.54536	3.065333	0.583106
C	–4.44758	1.100969	–0.30402
H	–5.51583	1.04393	–0.4931
C	2.735269	0.729333	0.247678
C	4.103706	2.580894	–0.28812
H	4.06972	2.997726	0.721331

H	5.091529	2.721308	–0.72659
H	3.337346	3.049973	–0.90981
C	0.361887	–1.32399	0.766979

Table S11. Cartesian coordinates for the geometry-optimized structure of ***p*–CF₃–PhCTC** (B3LYP/6-31G*).

S	0.91875	–2.03393	2.048141
O	4.285151	2.274751	–0.25456
O	2.979317	–0.6495	1.312561
O	2.181203	1.854029	0.464404
O	0.33272	–2.73235	–1.66111
N	1.56085	–1.2298	–0.4274
C	–0.9515	–1.06853	–0.5464
C	0.301566	–1.79208	–0.89486
C	3.924885	–0.70927	–0.91787
H	4.381886	–1.68087	–0.70552
H	4.562951	–0.18042	–1.63059
C	2.530502	–0.88438	–1.49841
H	2.183708	0.031662	–1.98897
H	2.512049	–1.69554	–2.22911
C	3.83403	0.062956	0.394939
H	4.803378	0.100698	0.89475
C	–2.17127	–1.71262	–0.80474
H	–2.16722	–2.7294	–1.18241
C	–0.9459	0.249201	–0.06566
H	–0.00932	0.761548	0.128023
C	–2.14975	0.911227	0.159091
H	–2.14431	1.930959	0.529041
C	–3.36072	0.257144	–0.08552
C	–3.37312	-1.0565	–0.56727

H	–4.31479	–1.562	–0.75399
C	3.313393	1.492076	0.221577
C	3.929977	3.655885	–0.50412
H	3.620673	4.138496	0.425994
H	4.835836	4.120362	–0.89316
H	3.123059	3.71286	–1.23865
C	1.854784	–1.24973	0.894221
C	–4.66108	0.988035	0.111892
F	–4.57096	1.946655	1.060164
F	–5.06644	1.605145	–1.02651
F	–5.66212	0.155661	0.476832

Table S12. Cartesian coordinates for the geometry-optimized structure of ***p*–MeO–PhCTC** (B3LYP/6-31G*).

S	0.380281	–2.07176	2.018516
O	–4.73433	1.849414	0.379932
O	4.087946	1.963193	–0.24385
O	2.531216	–0.91216	1.160838
O	1.989418	1.73829	0.572871
O	–0.57082	–2.58528	–1.55199
N	0.893856	–1.18117	–0.45811
C	–1.56746	–0.69997	–0.48781
C	–0.46192	–1.59925	–0.84933
C	3.236519	–0.86688	–1.16169
H	3.580147	–1.90391	–1.09447
H	3.861794	–0.34647	–1.89194
C	1.7779	–0.81052	–1.58745
H	1.500747	0.192965	–1.92984
H	1.583622	–1.51584	–2.39884
C	3.382418	–0.23085	0.217796

H	4.392596	–0.36717	0.607613
C	–2.87783	–1.10298	–0.79517
H	–3.03602	–2.07413	–1.25315
C	–1.36615	0.564732	0.101958
H	–0.36347	0.904906	0.33965
C	–2.44324	1.390199	0.373406
H	–2.29897	2.367596	0.823405
C	–3.75339	0.972492	0.070679
C	–3.96693	–0.28573	–0.51926
H	–4.96711	–0.62522	–0.76152
C	3.049112	1.263142	0.221921
C	3.898936	3.394131	–0.35031
H	3.701907	3.822799	0.63515
H	4.835068	3.778918	–0.75439
H	3.068928	3.617918	–1.02491
C	1.301146	–1.33534	0.816973
C	–6.09213	1.491774	0.104876
H	–6.388	0.596476	0.663623
H	–6.69192	2.341493	0.435502
H	–6.25017	1.32865	–0.96746

Table S13. Cartesian coordinates for the geometry-optimized structure of ***t*BuOCTC** (B3LYP/6-31G*).

S	–0.74345	2.449608	–1.05586
O	3.932403	–1.06805	–0.13162
O	1.644509	1.663901	–0.53599
O	–1.83531	–0.28723	–0.21887
O	–1.62156	–0.31235	2.047597
O	2.010492	–0.94559	–1.32499
N	0.048852	0.567124	0.741959
C	2.807869	–0.47146	–0.54681

C	–1.24147	–0.02298	0.930107
C	0.345305	1.477145	–0.24343
C	2.678191	0.903699	0.115958
H	3.594373	1.466845	–0.07392
C	1.020342	0.163493	1.800158
H	1.124773	–0.92524	1.753561
H	0.58338	0.420305	2.766378
C	2.363783	0.845834	1.60535
H	2.343909	1.873352	1.98206
H	3.138877	0.299401	2.148359
C	4.189052	–2.38531	–0.67162
H	3.378145	–3.06863	–0.40819
H	5.124511	–2.7077	–0.21453
H	4.291988	–2.33418	–1.75829
C	–3.481	–0.90368	–1.77977
H	–4.47089	–1.33405	–1.96403
H	–3.45561	0.103571	–2.20738
H	–2.73499	–1.51903	–2.29355
C	–3.18924	–2.27507	0.314753
H	–2.98485	–2.25783	1.387125
H	–4.16519	–2.74675	0.154615
H	–2.43008	–2.88898	–0.1826
C	–3.2107	–0.86478	–0.27652
C	–4.19464	0.062994	0.43623
H	–4.1337	1.076535	0.026225
H	–5.21294	–0.30878	0.277249
H	–4.0077	0.102748	1.511421

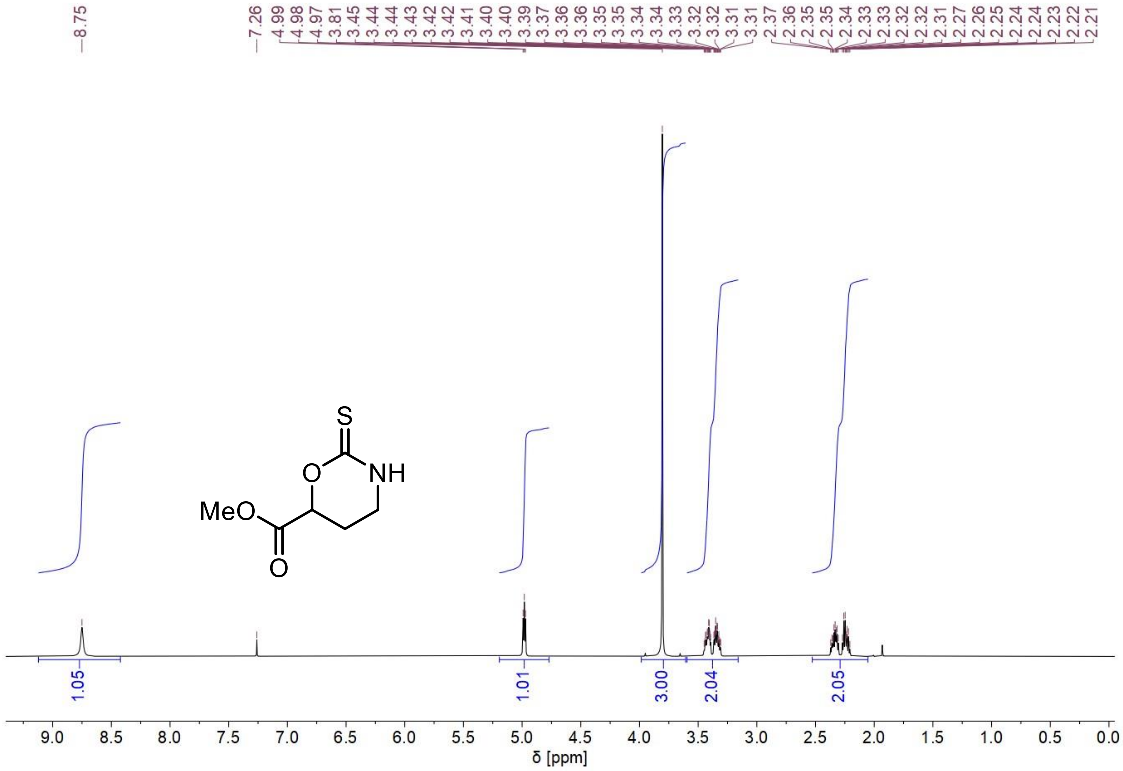

Figure S11. ^{1}H NMR Spectrum of **II** ($CDCl_3$, 500.1 MHz).

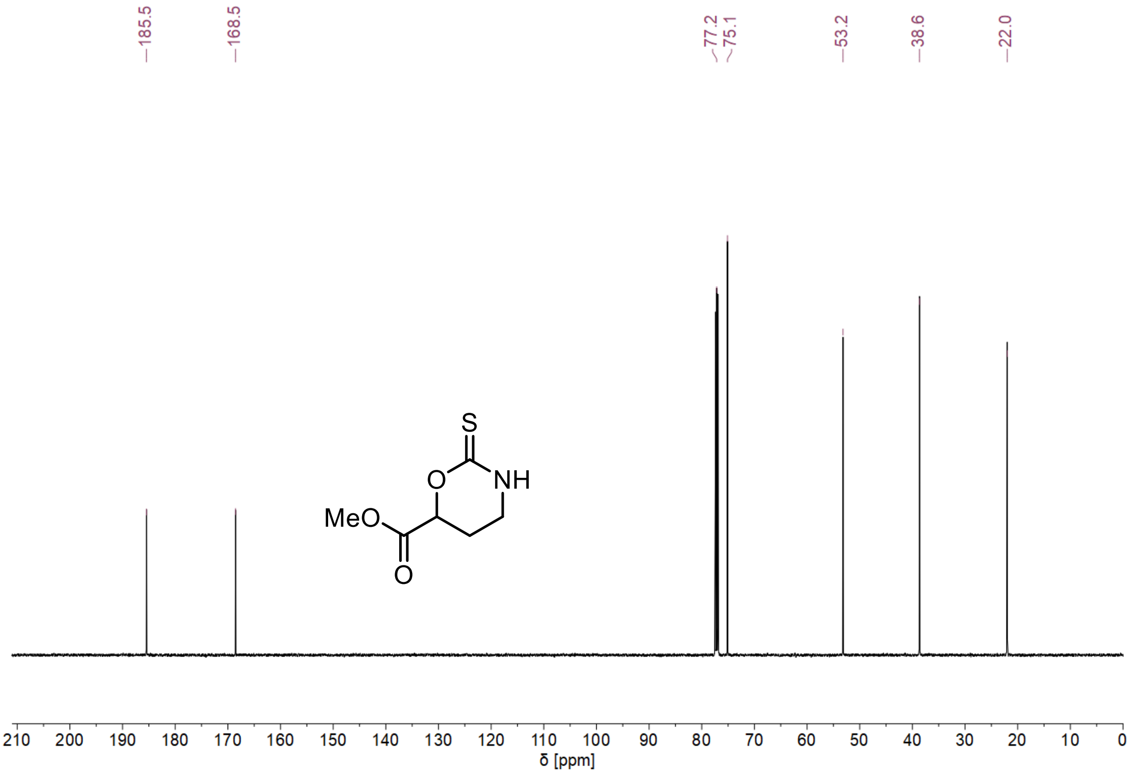

Figure S12. ^{13}C NMR Spectrum of **II** ($CDCl_3$, 125.8 MHz).

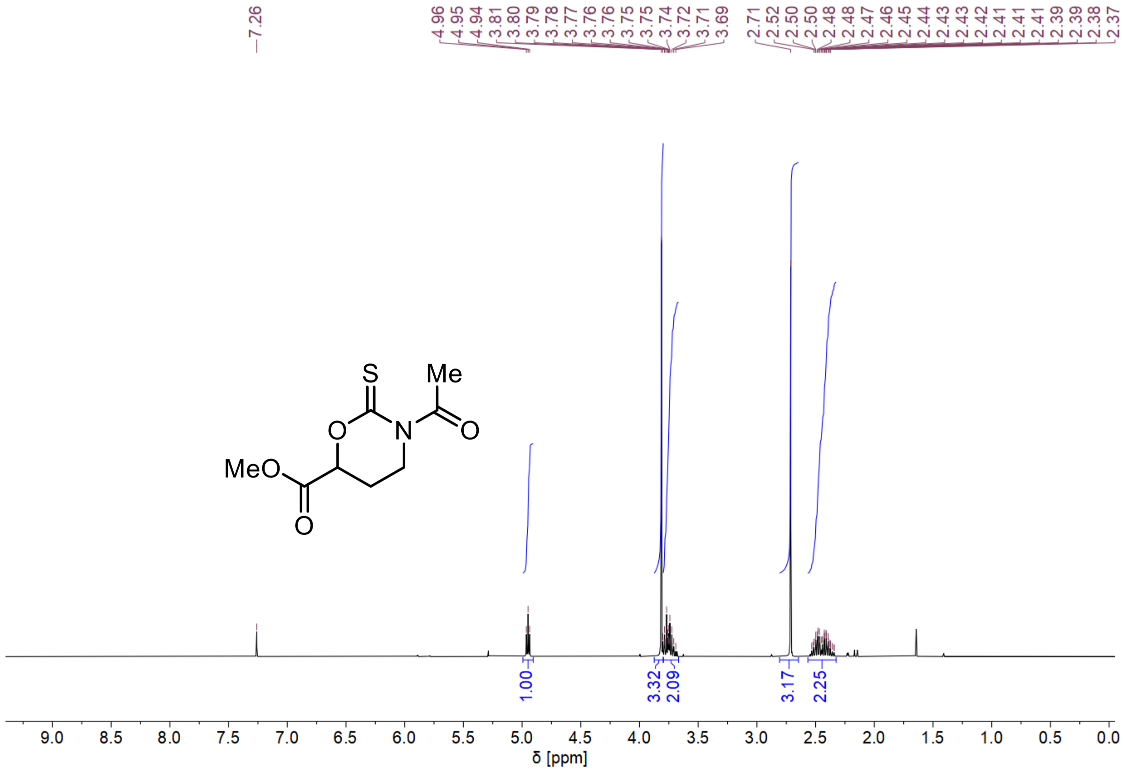

Figure S13. ^{1}H NMR Spectrum of **MeCTC** ($CDCl_3$, 400.1 MHz)

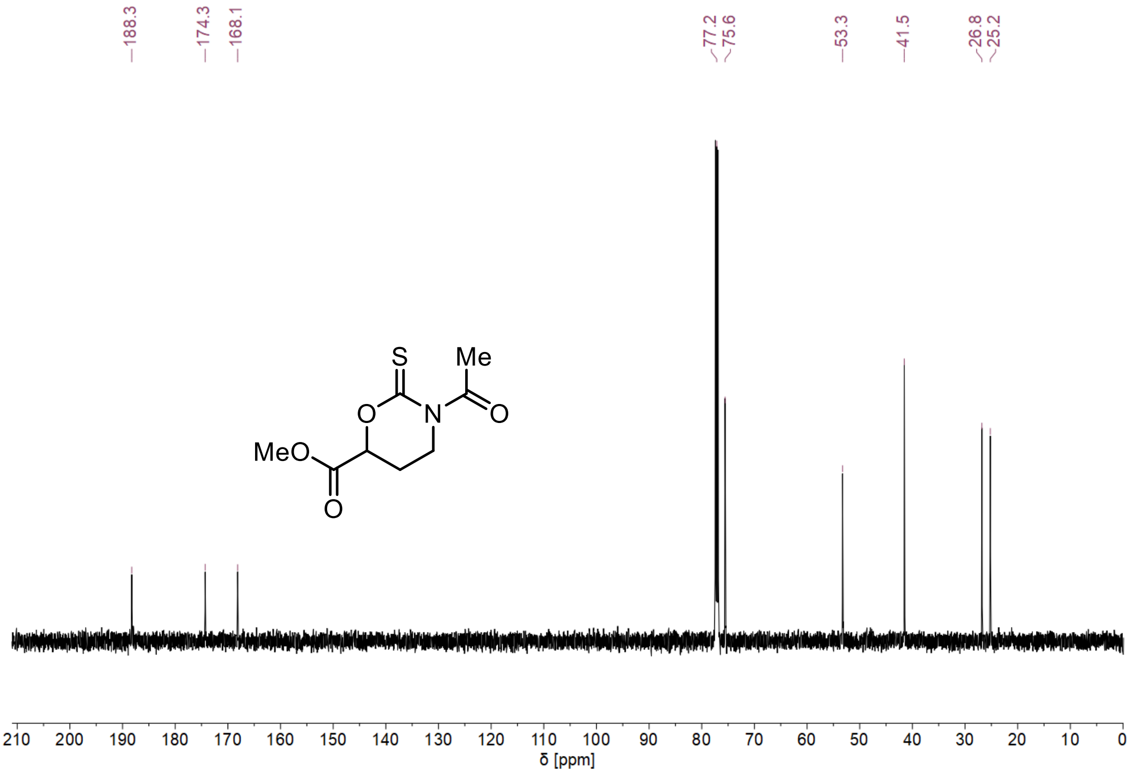

Figure S14. ^{13}C NMR Spectrum of **MeCTC** ($CDCl_3$, 125.8 MHz).

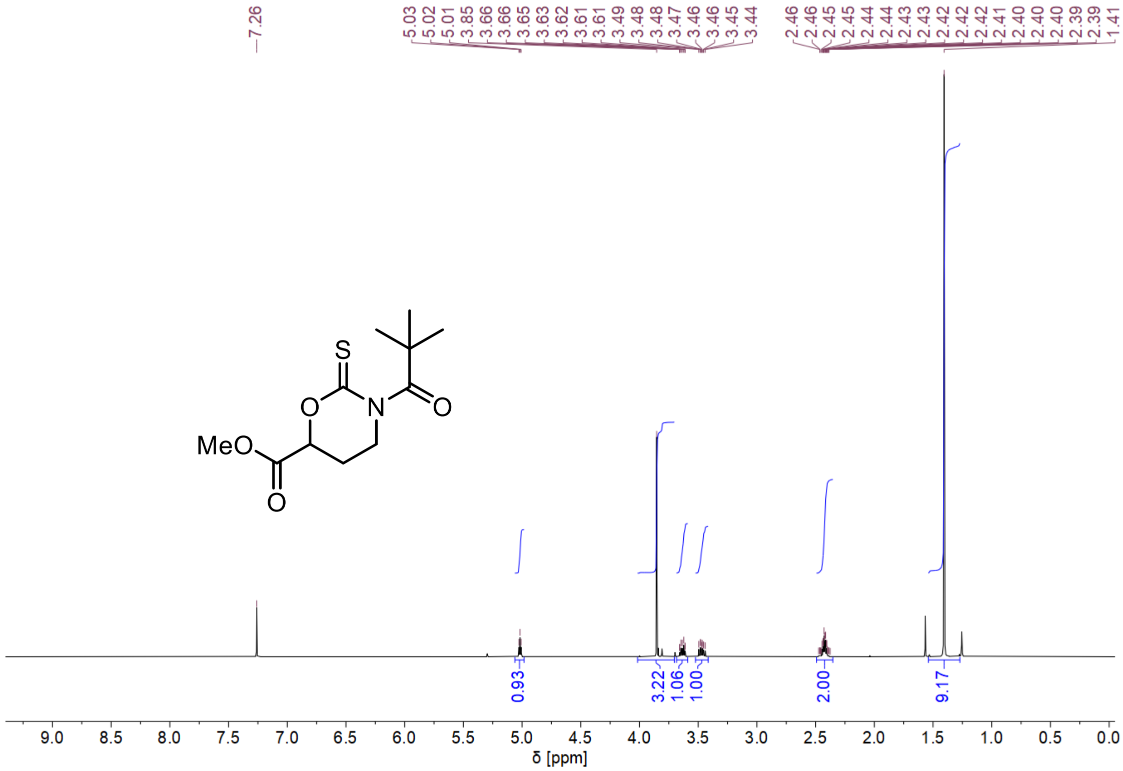

Figure S15. ^{1}H NMR Spectrum of ***^{t}*BuCTC** ($CDCl_3$, 500.1 MHz).

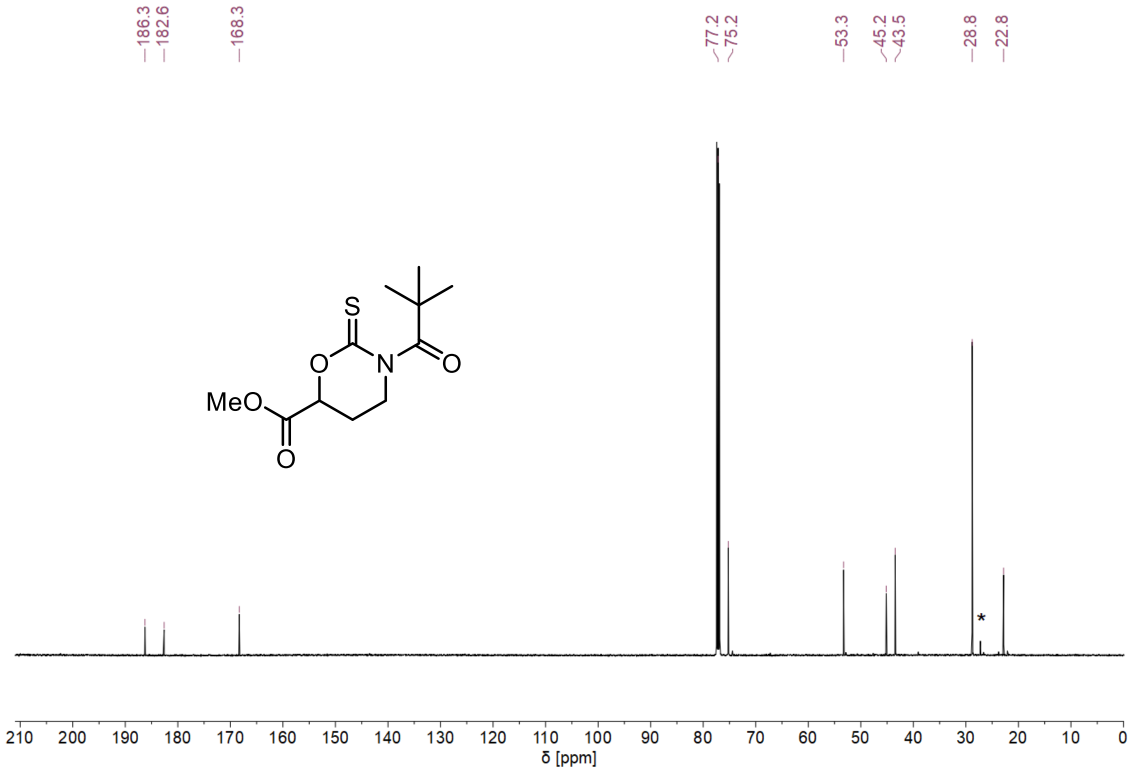

Figure S16. ^{13}C NMR Spectrum of ***^{t}*BuCTC** ($CDCl_3$, 125.8 MHz) *) indicates residual grease.

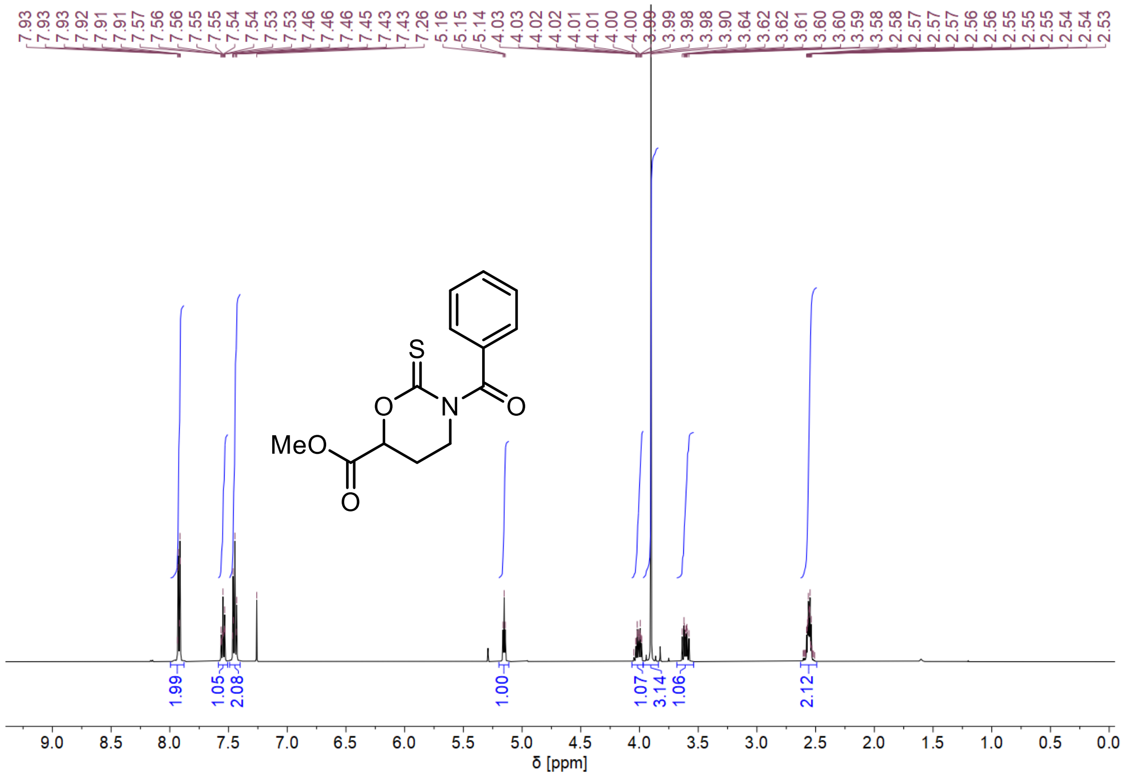

Figure S17. ^{1}H NMR Spectrum of **PhCTC** ($CDCl_3$, 500.1 MHz).

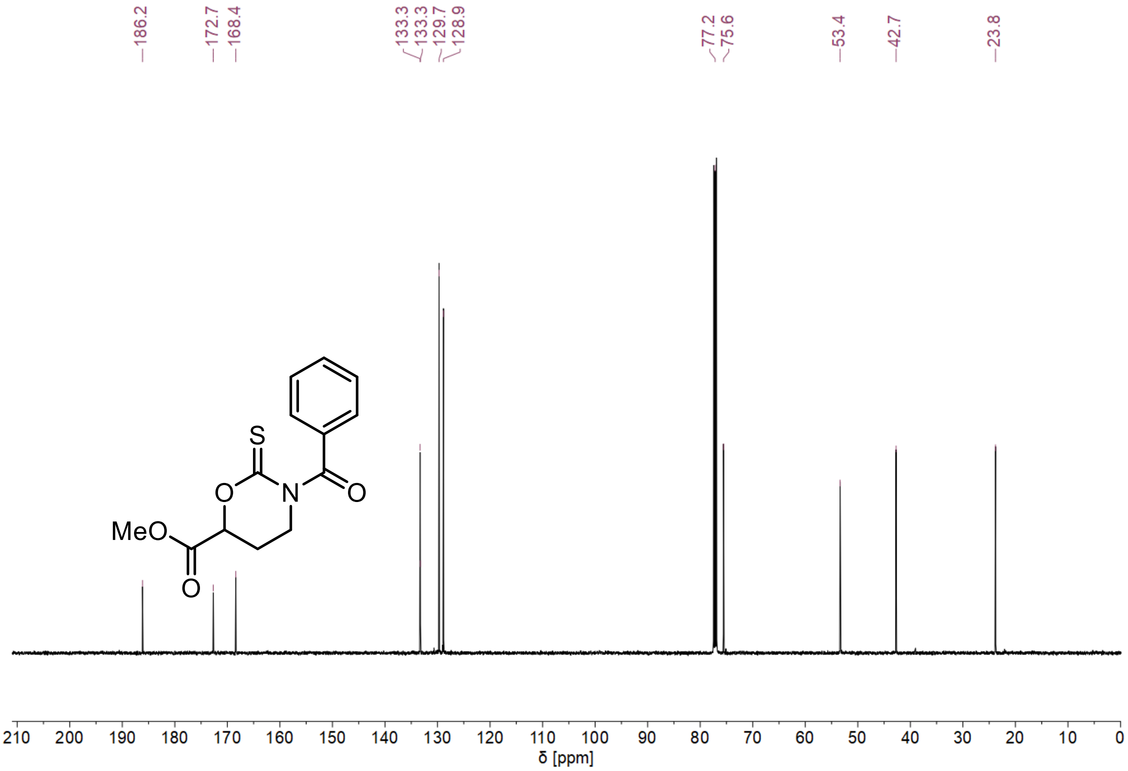

Figure S18. ^{13}C NMR Spectrum of **PhCTC** ($CDCl_3$, 125.8 MHz).

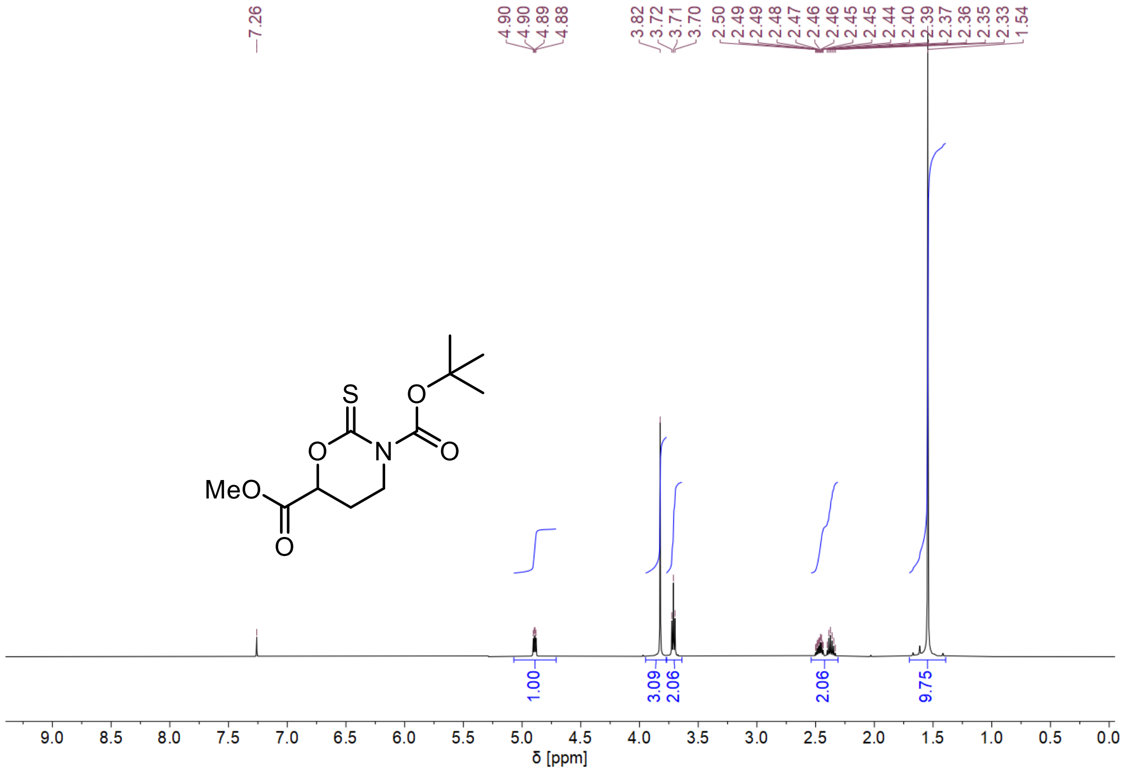

Figure S19. ^{1}H NMR Spectrum of ***^t^*BuOCTC** ($CDCl_3$, 500.1 MHz).

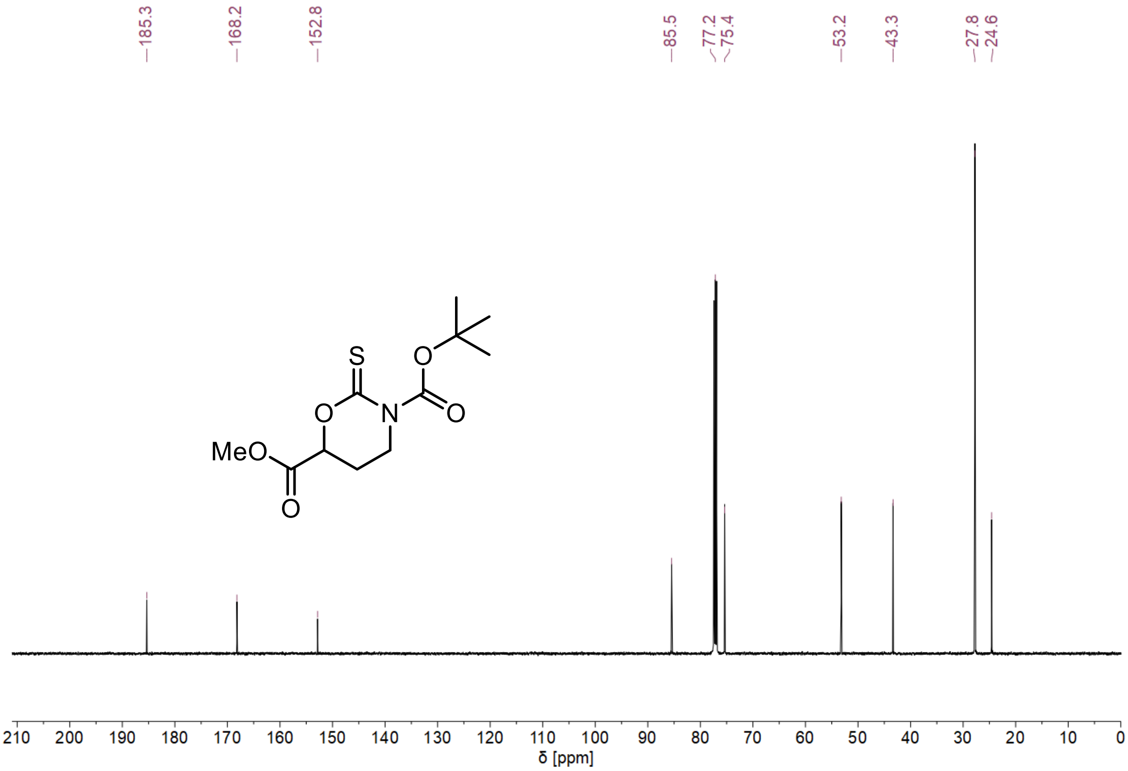

Figure S20. ^{13}C NMR Spectrum of ***^t^*BuOCTC** ($CDCl_3$, 125.8 MHz).

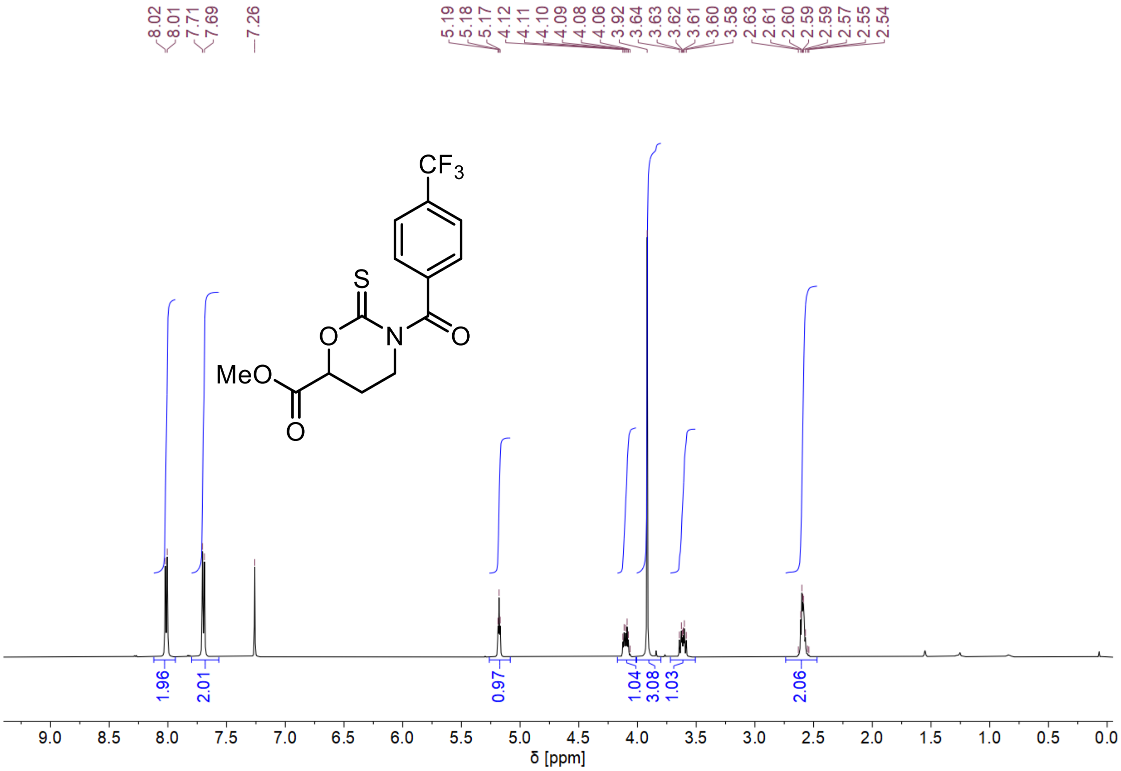

Figure S21. ^{1}H NMR Spectrum of ***p*–CF3–PhCTC** ($CDCl_3$, 500.1 MHz).

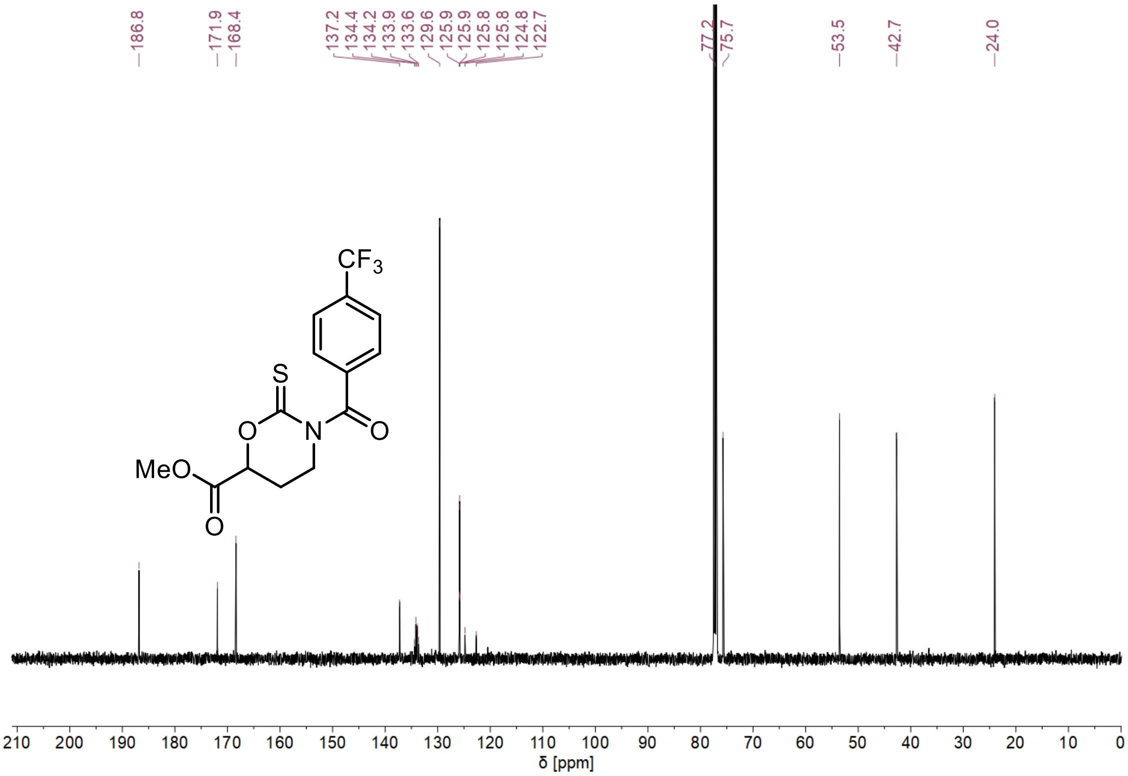

Figure S22. ^{13}C NMR Spectrum of ***p*–CF3–PhCTC** ($CDCl_3$, 125.8 MHz).

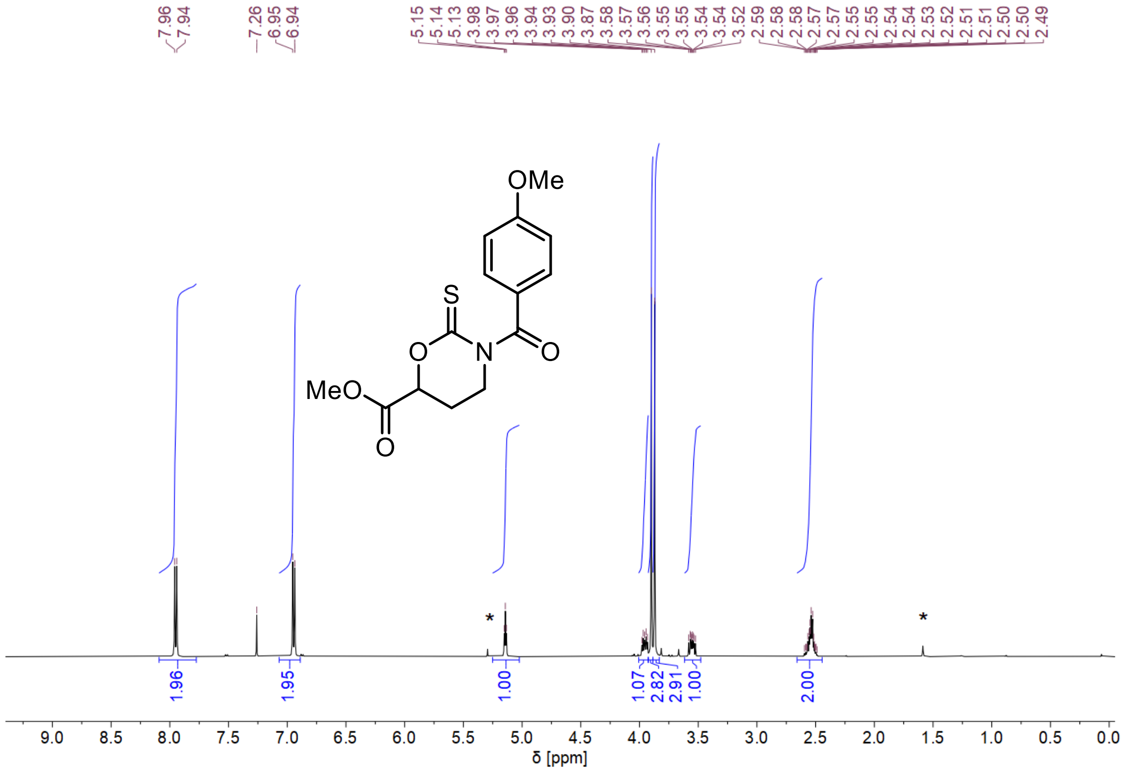

Figure S23. ^{1}H NMR Spectrum of ***p*–MeO–PhCTC** ($CDCl_3$, 500.1 MHz) *) indicates residual CH_2Cl_2 and water.

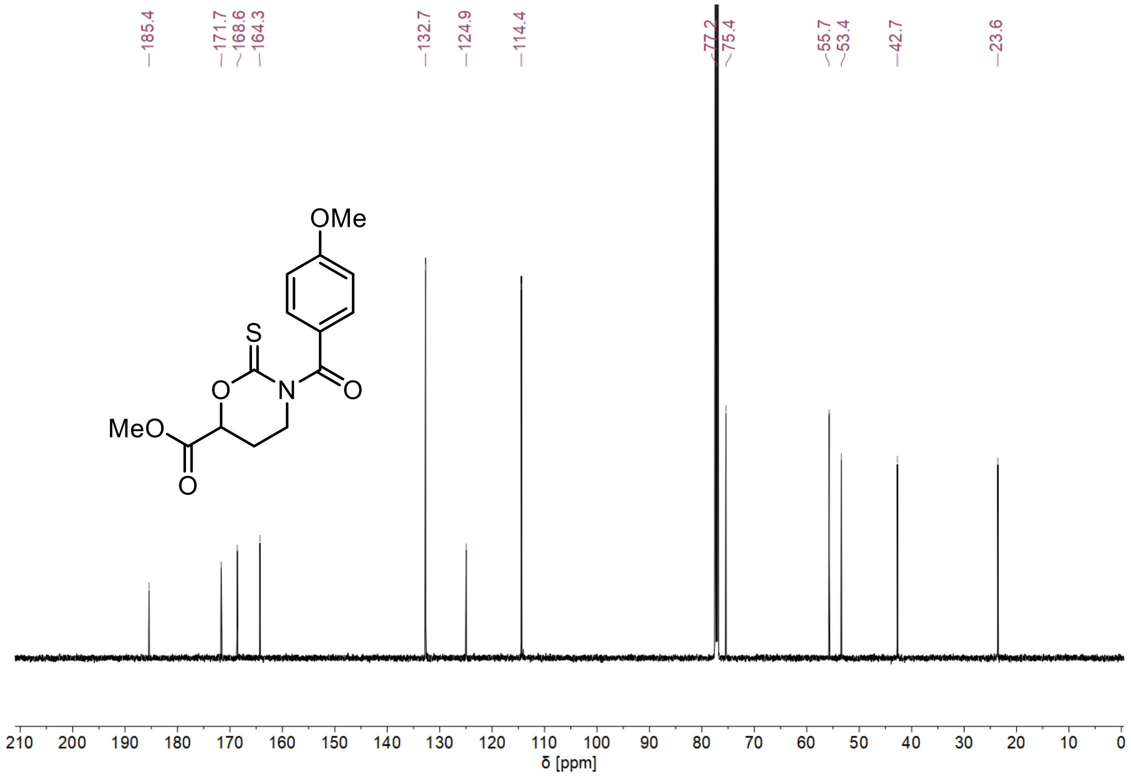

Figure S24. ^{13}C NMR Spectrum of ***p*–MeO–PhCTC** ($CDCl_3$, 125.8 MHz).

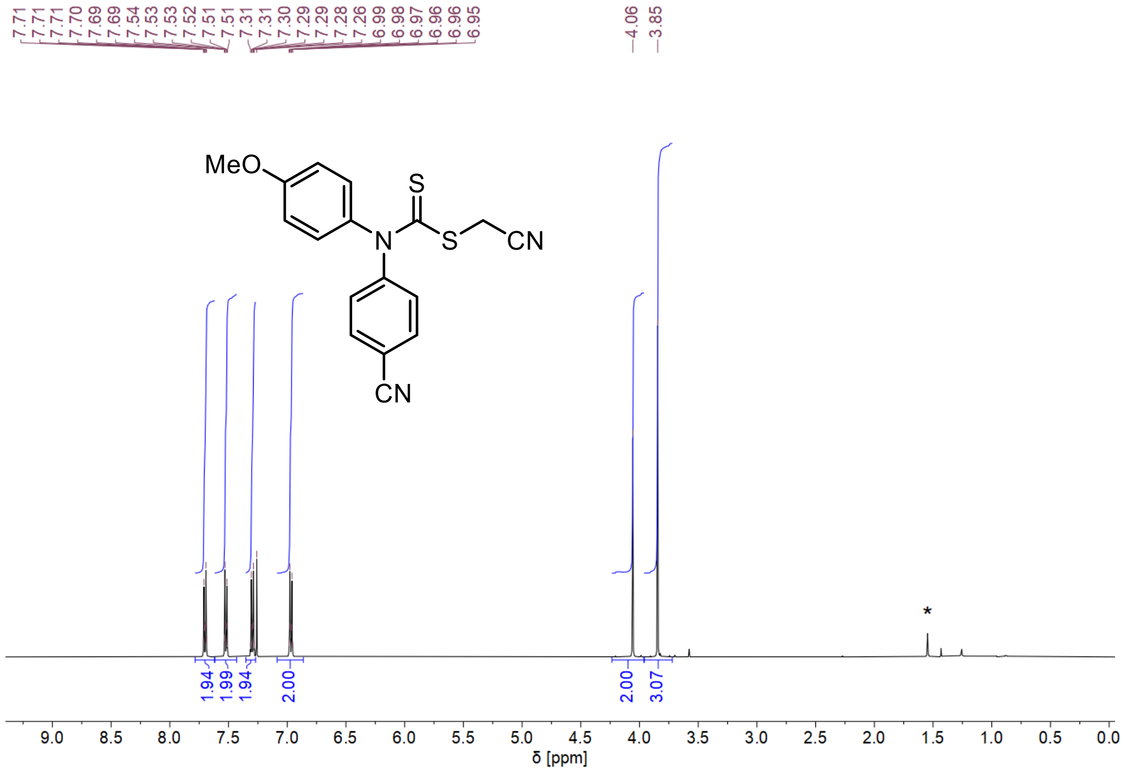

Figure S25. ^{1}H NMR Spectrum of **R4** ($CDCl_3$, 500.1 MHz) *) indicates residual water.

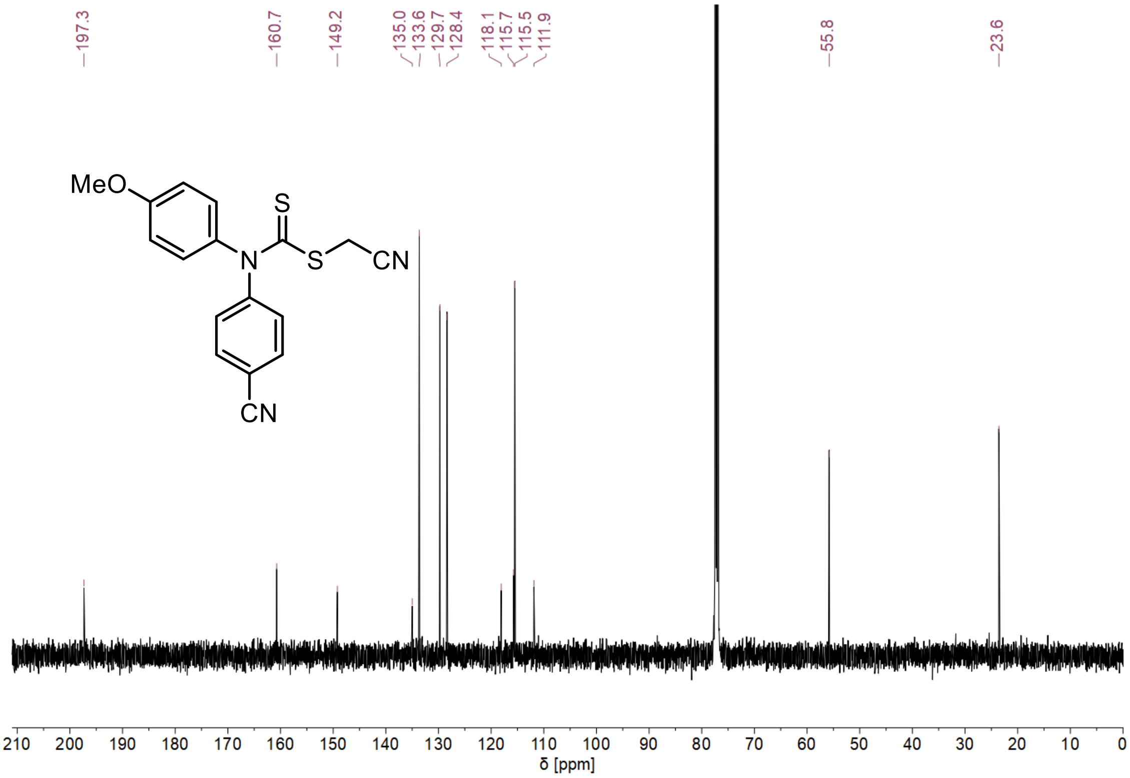

Figure S26. ^{13}C NMR Spectrum of **R4** ($CDCl_3$, 125.8 MHz).

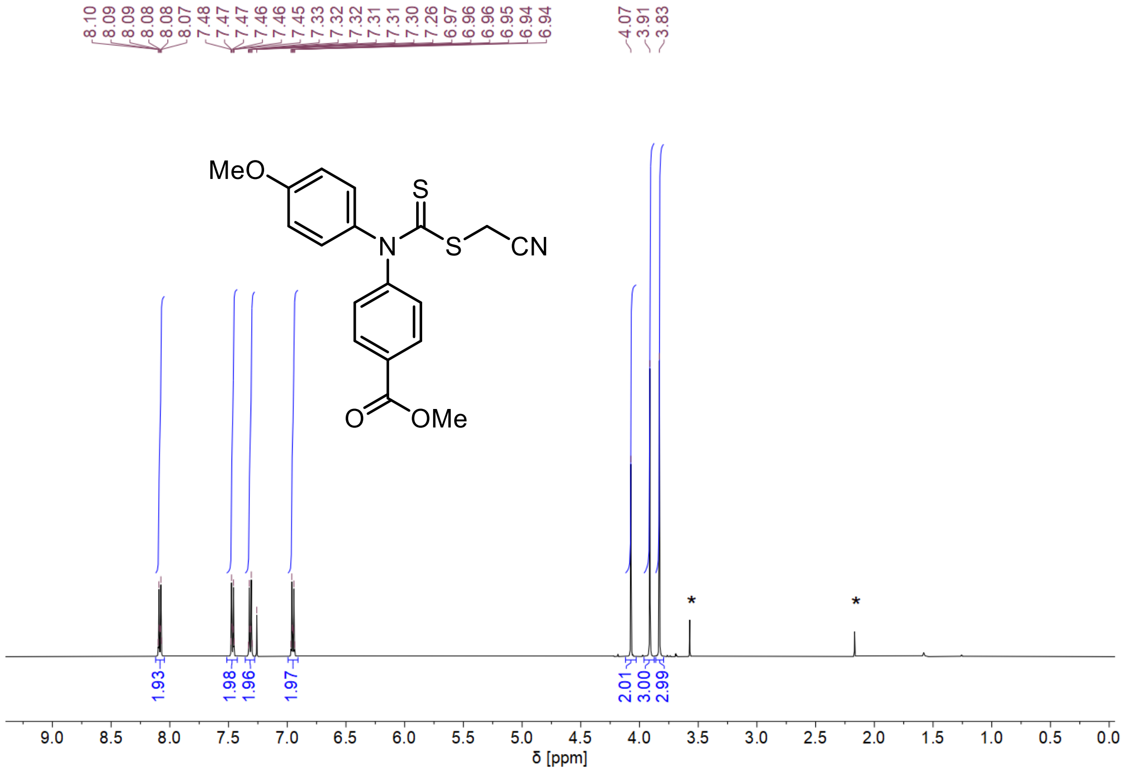

Figure S27. ^{1}H NMR Spectrum of **R5** ($CDCl_3$, 500.1 MHz) *) indicates residual acetone and methanol.

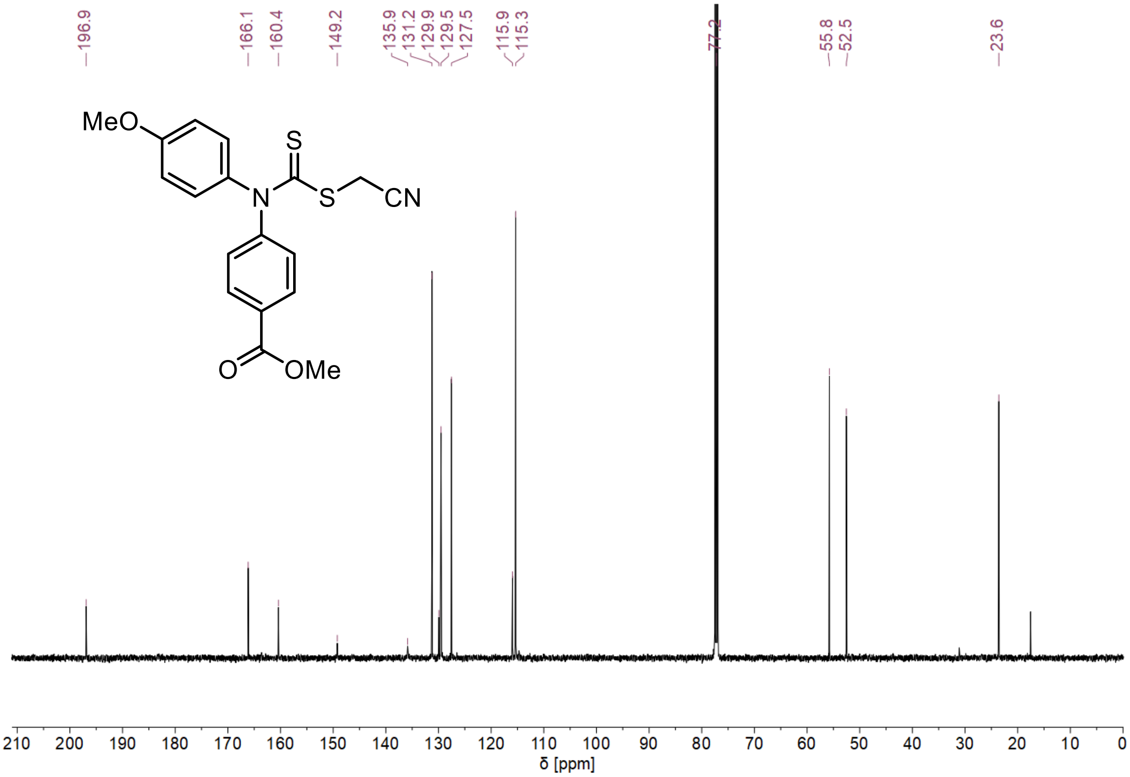

Figure S28. ^{13}C NMR Spectrum of **R5** ($CDCl_3$, 125.8 MHz)

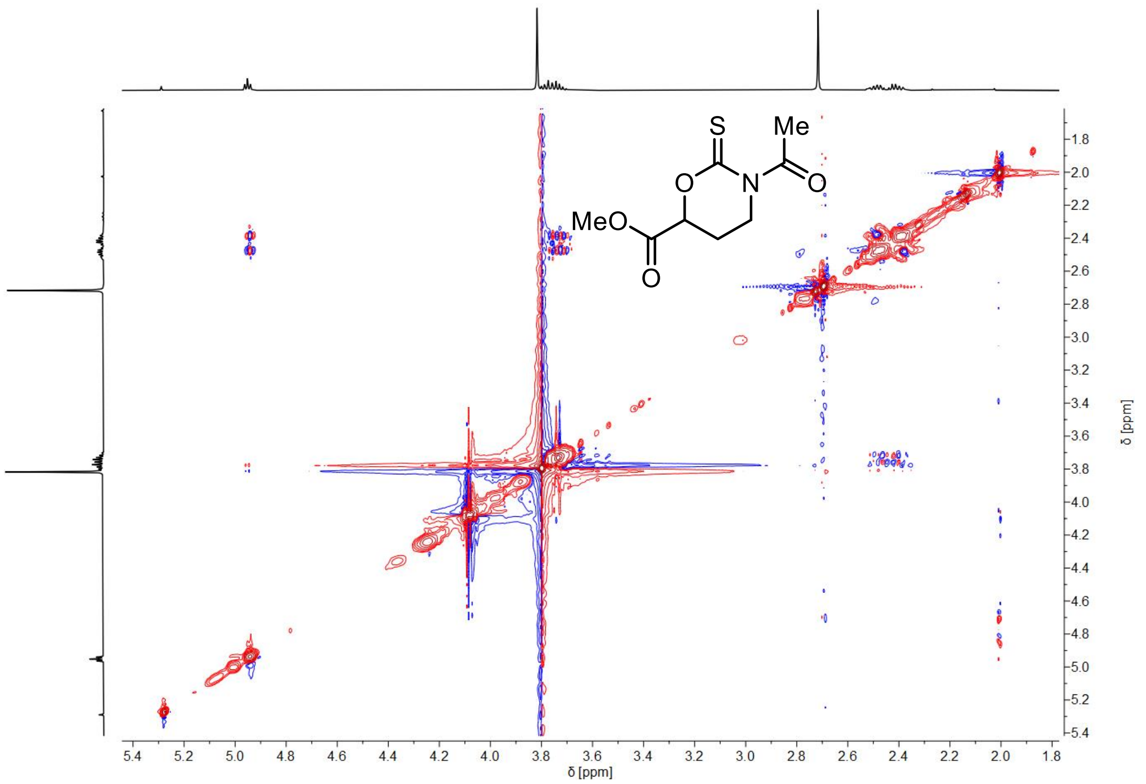

Figure S29. ROESY NMR Spectrum of **MeCTC** ($CDCl_3$, 500.1 MHz).

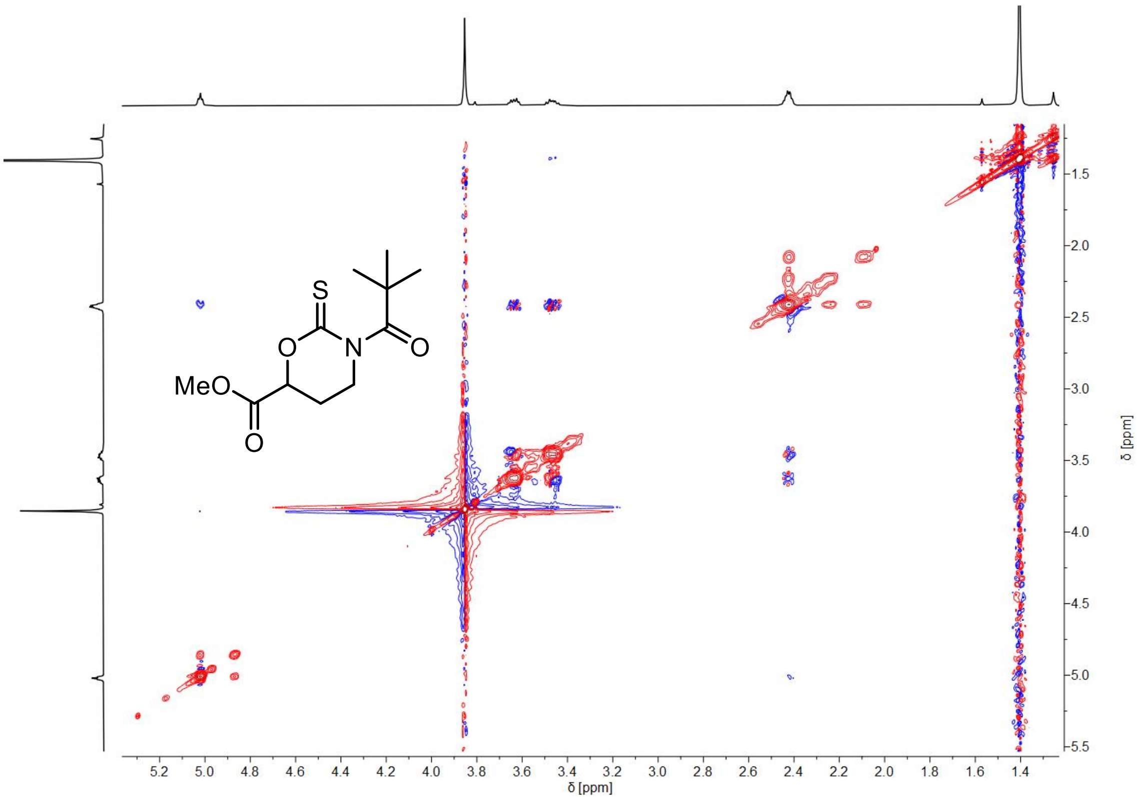

Figure S30. ROESY NMR Spectrum of ***t*BuCTC** ($CDCl_3$, 500.1 MHz).

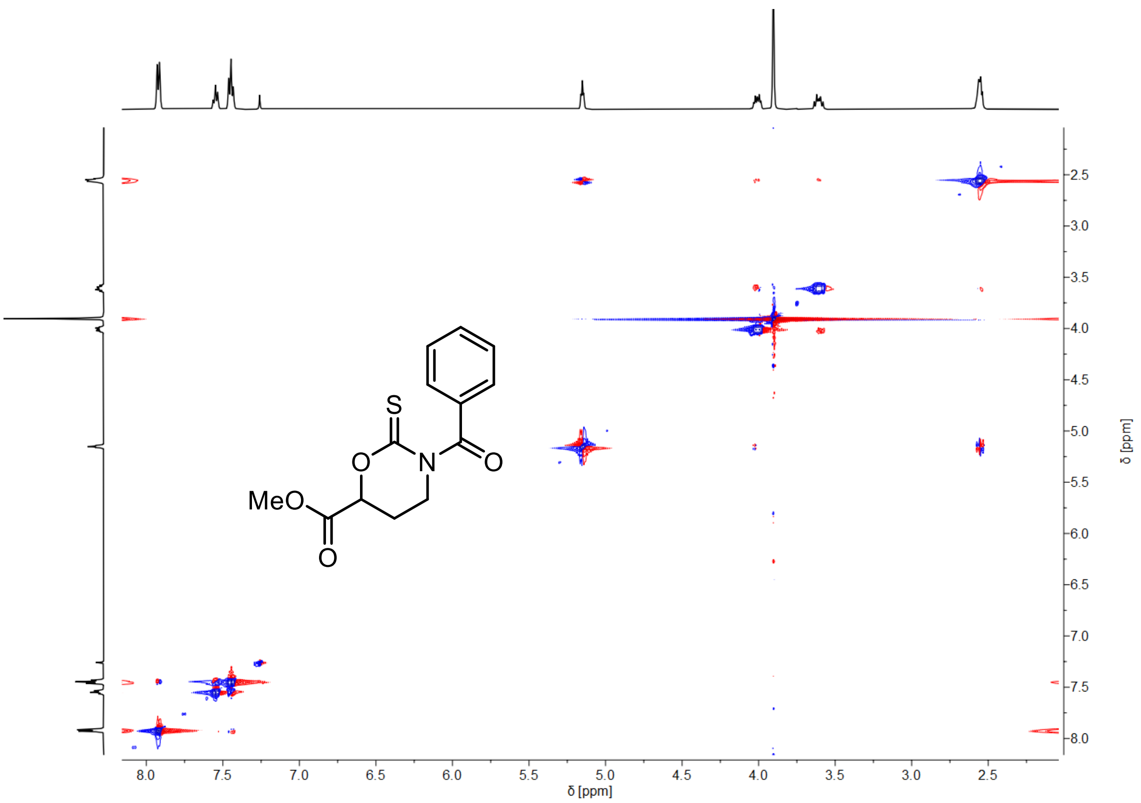

Figure S31. ROESY NMR Spectrum of **PhCTC** ($CDCl_3$, 500.1 MHz).

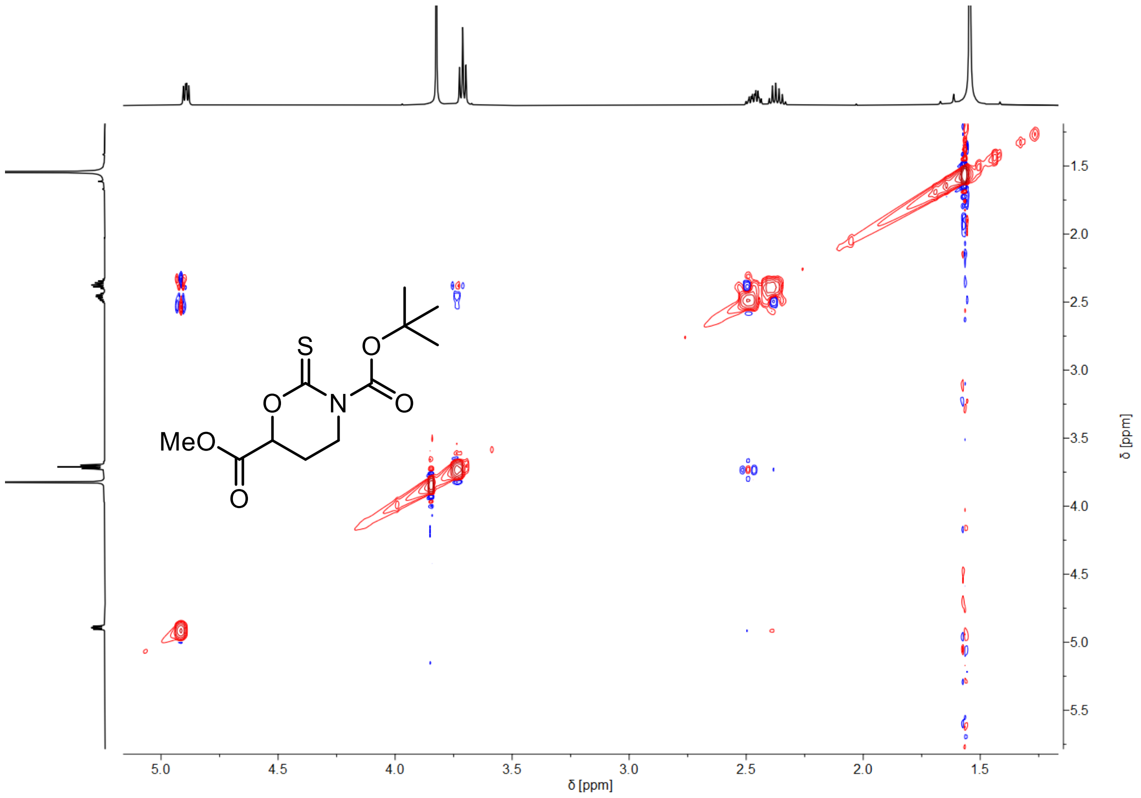

Figure S32. NOESY NMR Spectrum of ***t*BuOCTC** ($CDCl_3$, 500.1 MHz).

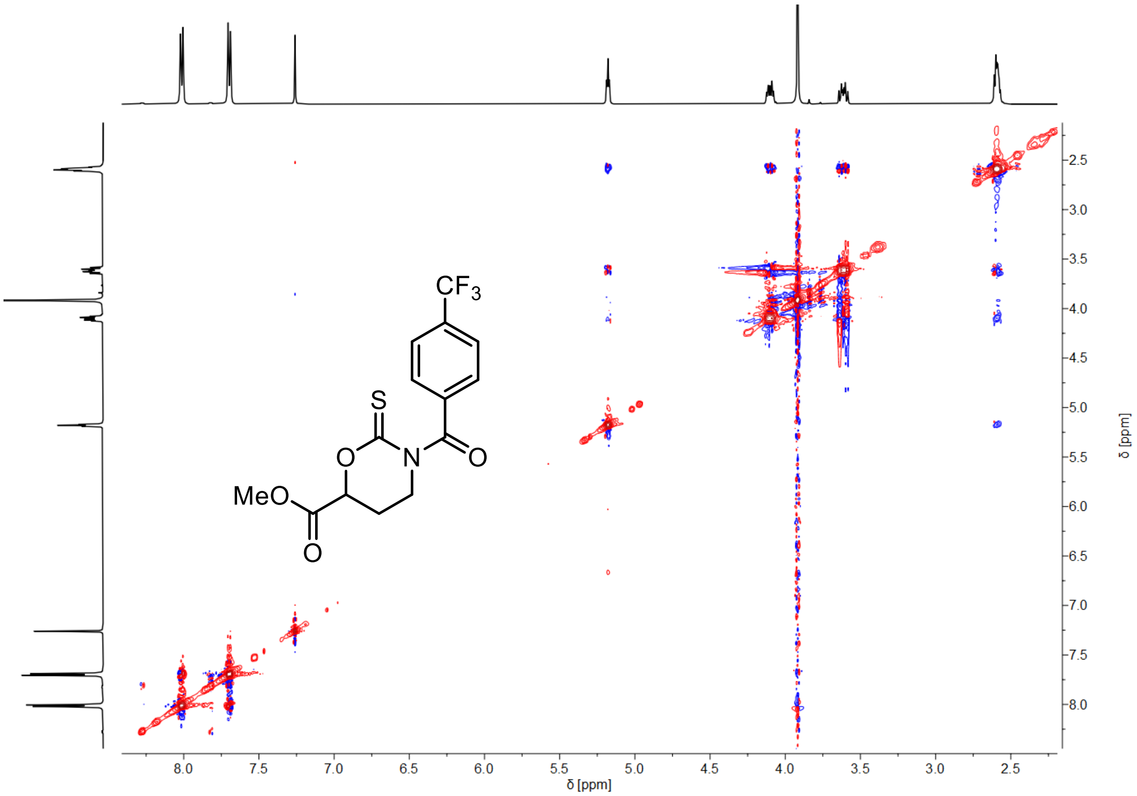

Figure S33. ROESY NMR Spectrum of ***p*–CF₃–PhCTC** (CDCl₃, 500.1 MHz).

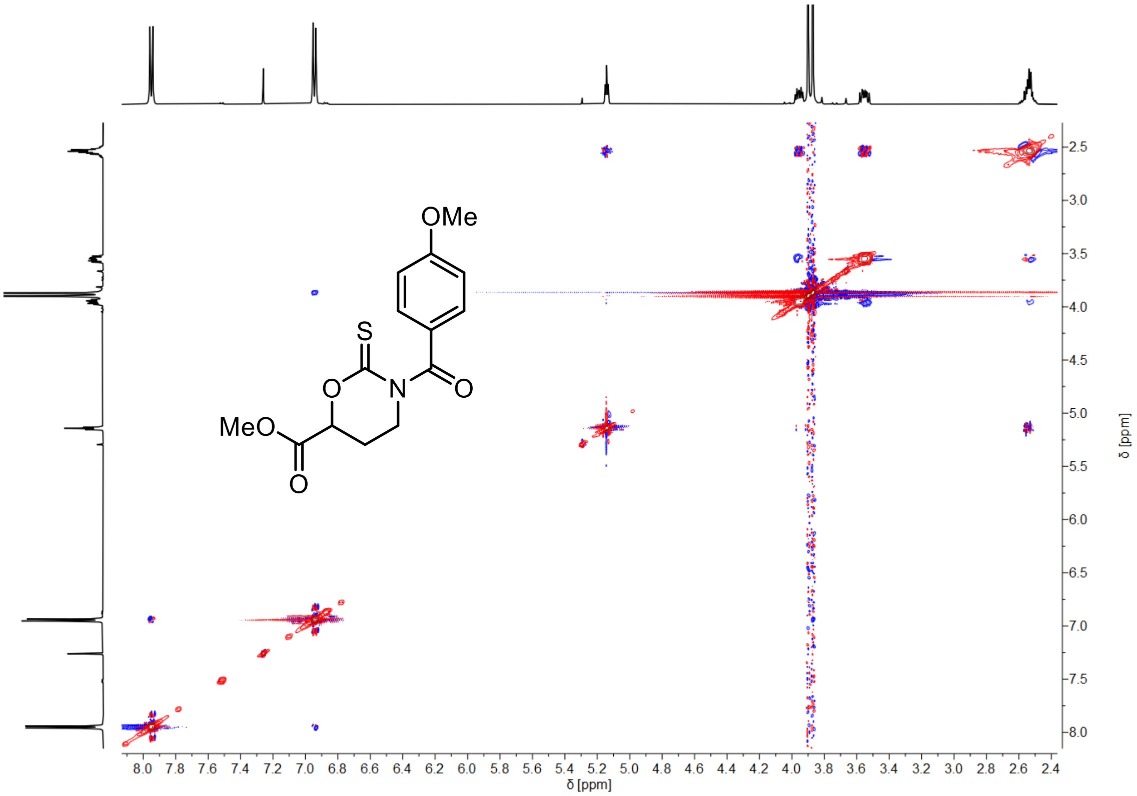

Figure S34. ROESY NMR Spectrum of ***p*–MeO–PhCTC** (CDCl₃, 500.1 MHz).

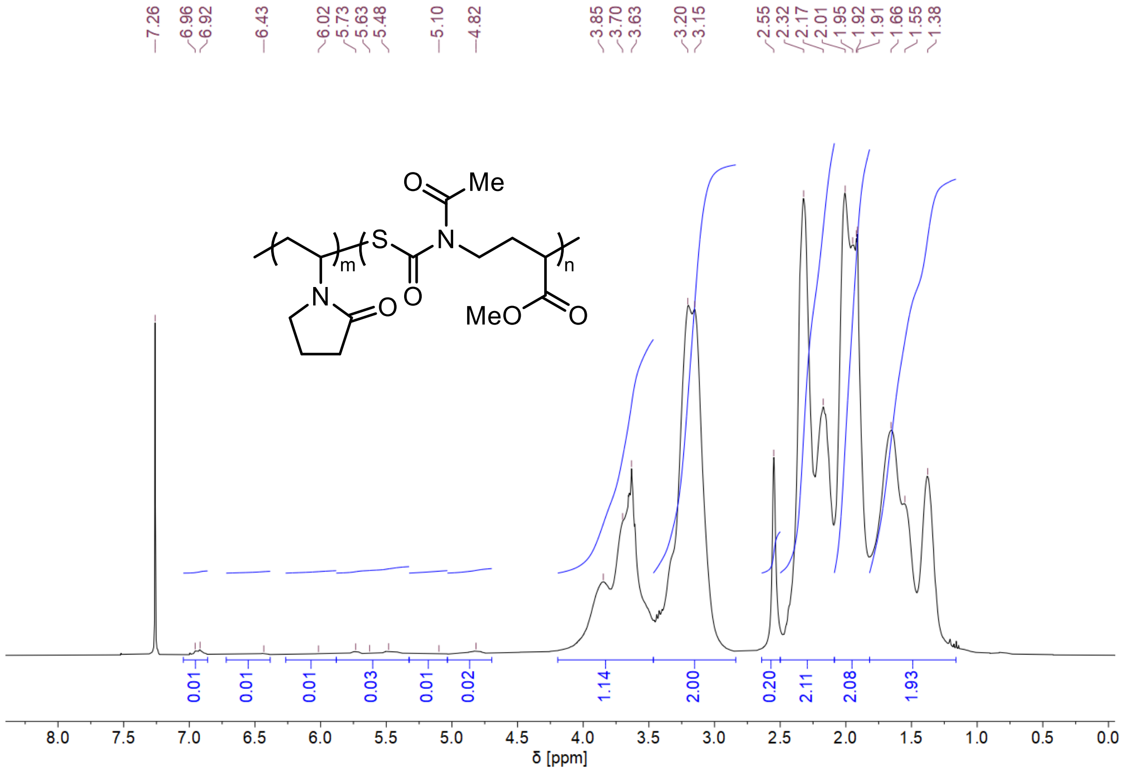

Figure S35. ^{1}H NMR spectrum of **MeCTC–*co*–PVP** ($CDCl_3$, 400.1 MHz)

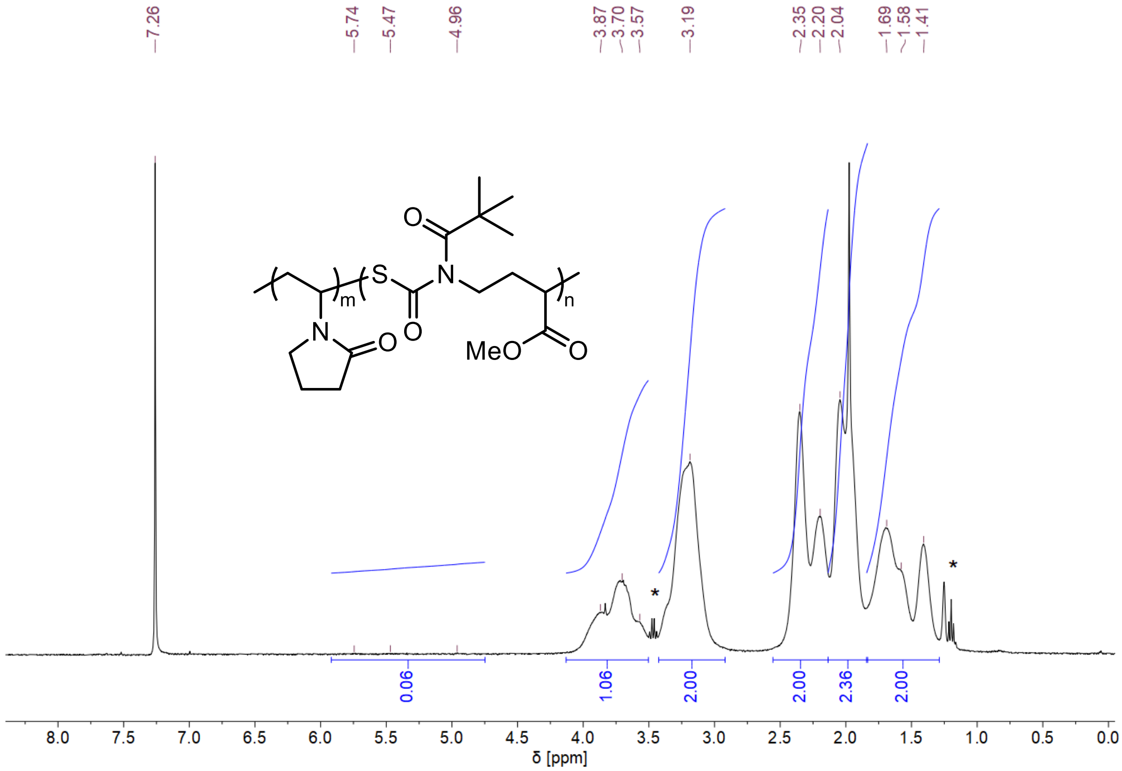

Figure S36. ^{1}H NMR spectrum of ***^{t}*BuCTC–*co*–PVP** ($CDCl_3$, 500.1 MHz) *) indicates residual diethyl ether.

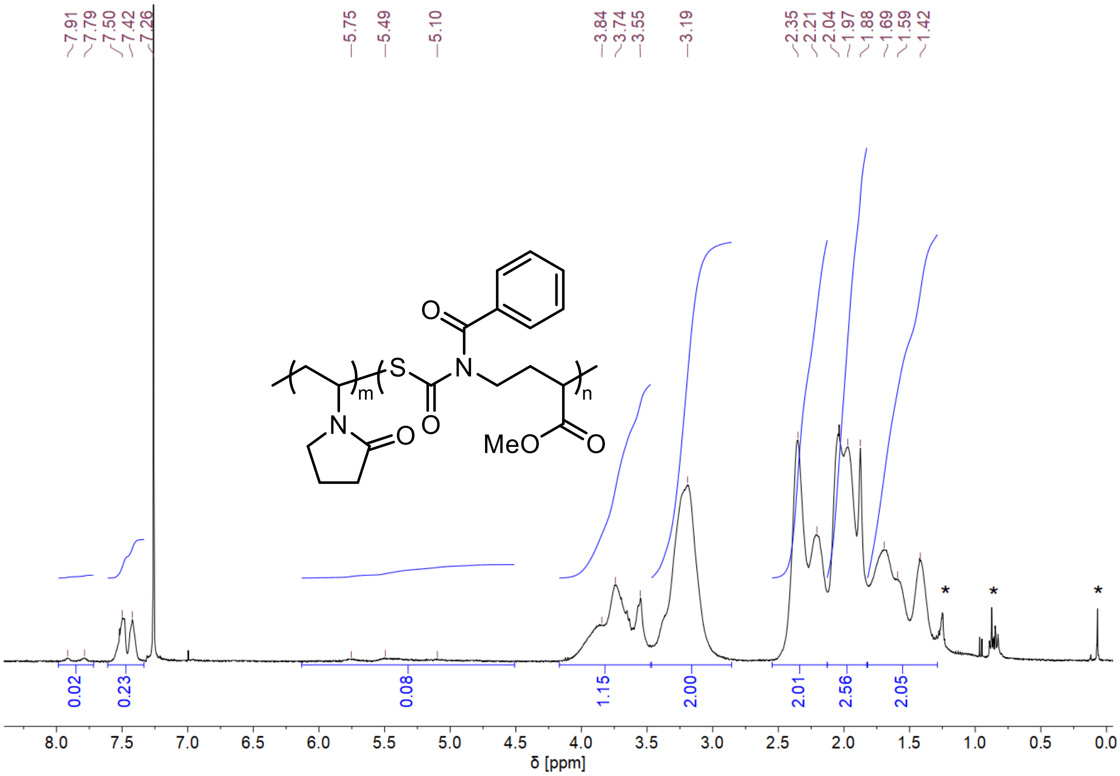

Figure S37. ^{1}H NMR spectrum of **PhCTC–*co*–PVP** ($CDCl_3$, 400.1 MHz) *) indicates residual hexanes and grease.

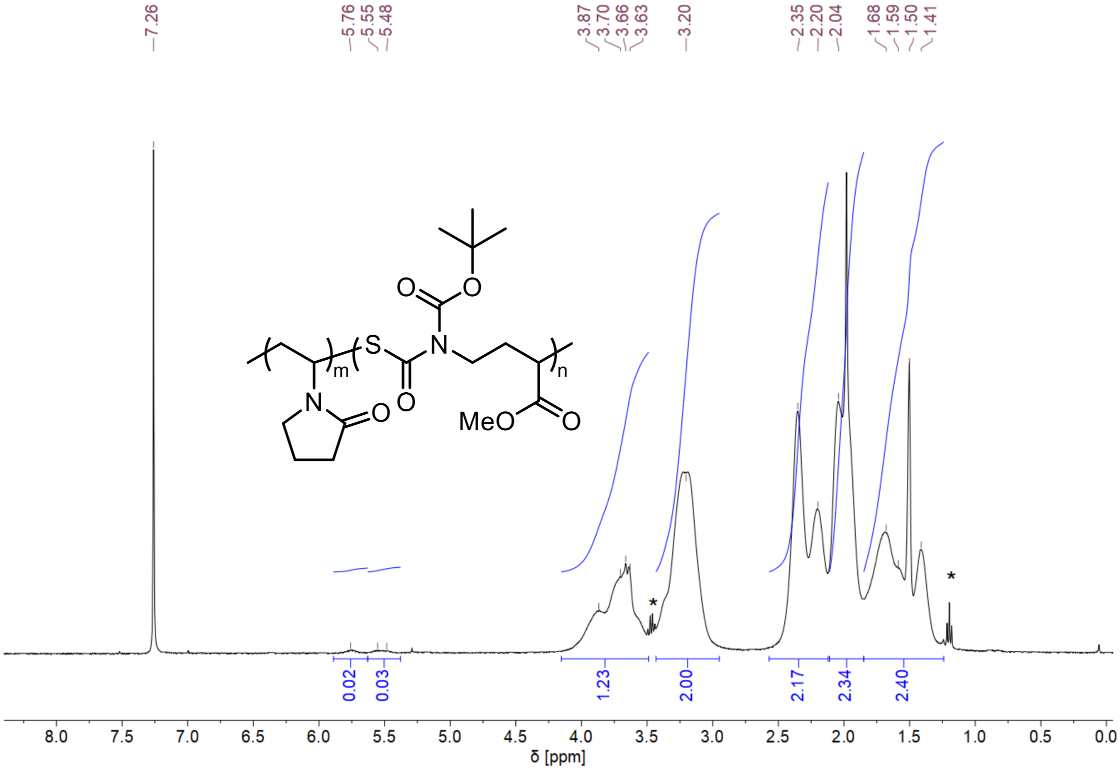

Figure S38. ^{1}H NMR spectrum of ***t*BuOCTC–*co*–PVP** ($CDCl_3$, 500.1 MHz) *) indicates residual diethyl ether.

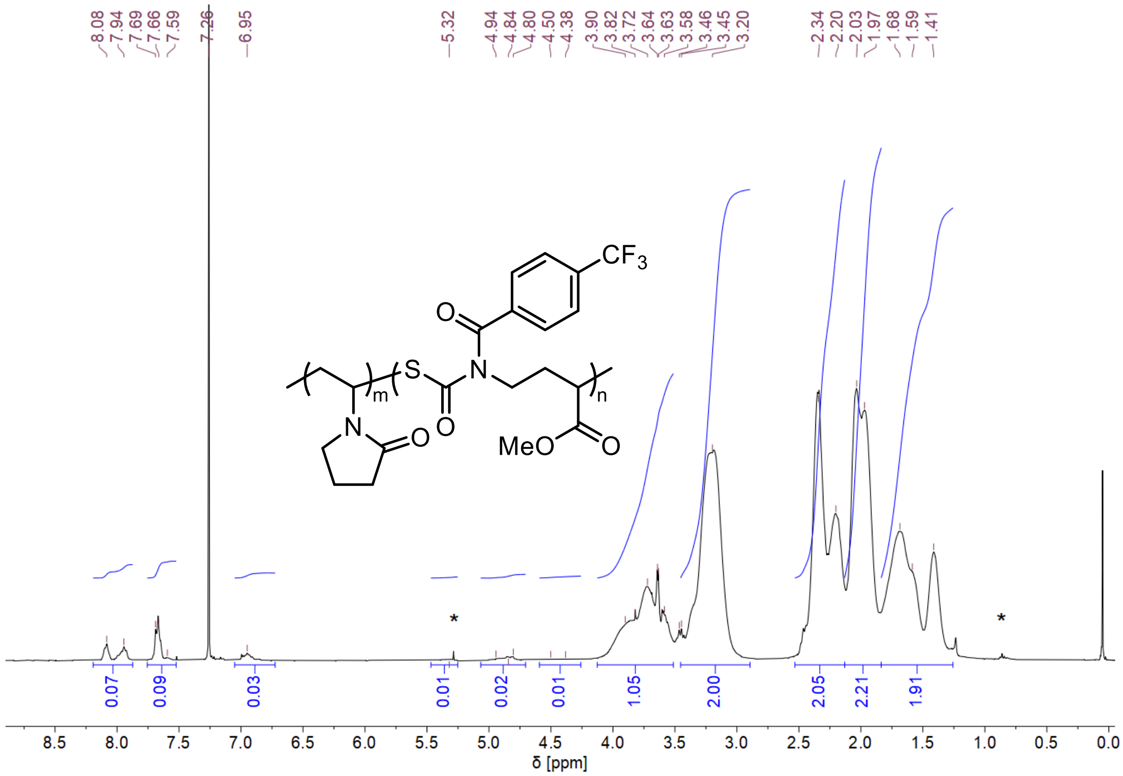

Figure S39. ^{1}H NMR spectrum of ***p*–CF₃–PhCTC–*co*–PVP** (CDCl₃, 400.1 MHz) *) indicates residual hexanes and CH_2Cl_2.

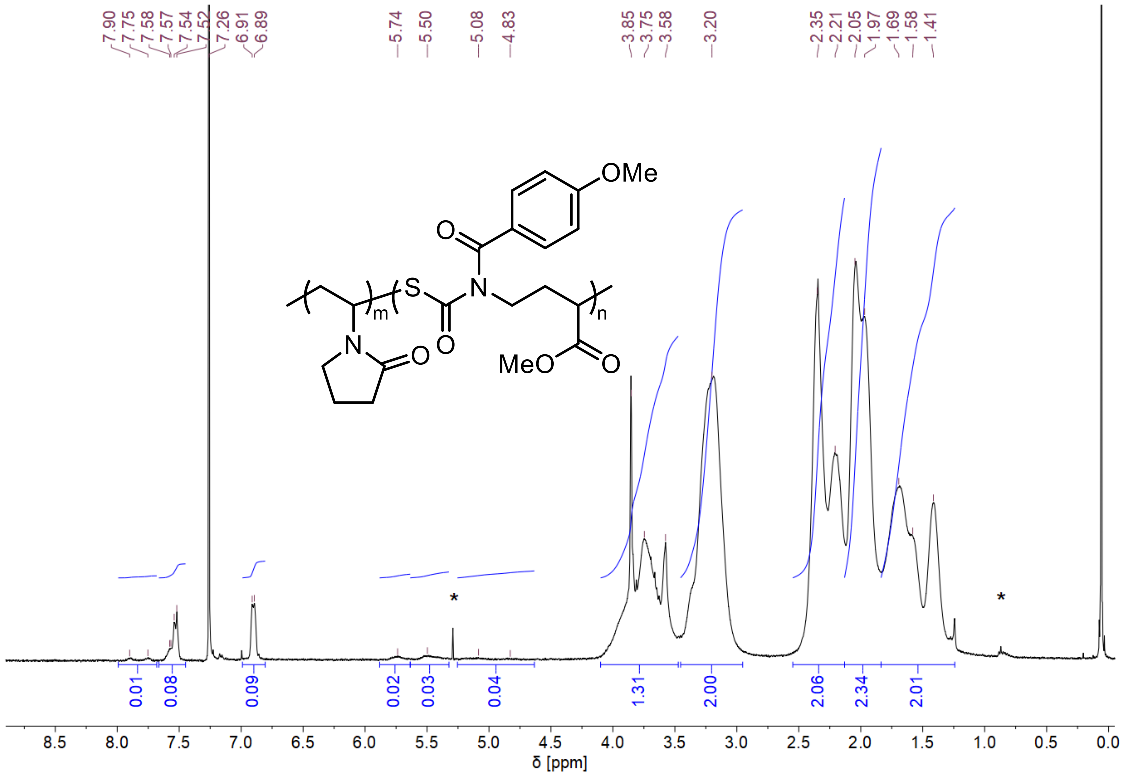

Figure S40. ^{1}H NMR spectrum of ***p*–MeO–PhCTC–*co*–PVP** (CDCl₃, 400.1 MHz) *) indicates residual hexanes and CH_2Cl_2.

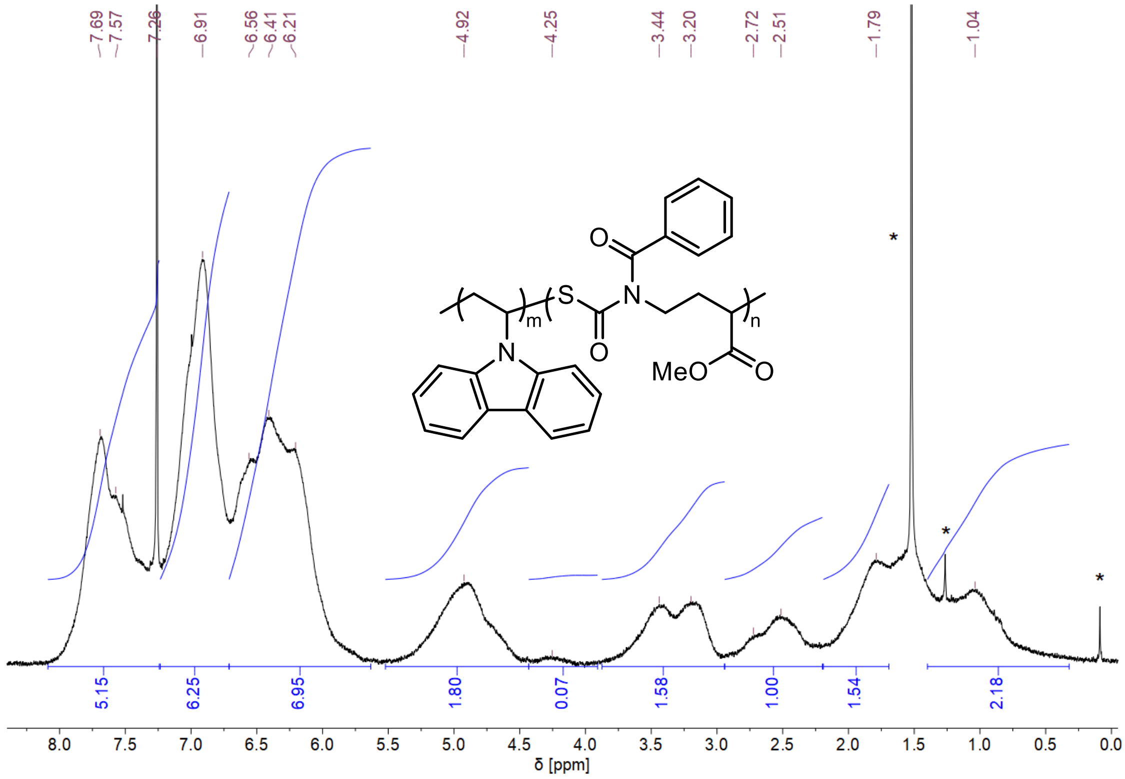

Figure S41. ^{1}H NMR spectrum of **PhCTC–*co*–PVC** ($CDCl_3$, 400.1 MHz) *) indicates residual hexanes and grease.

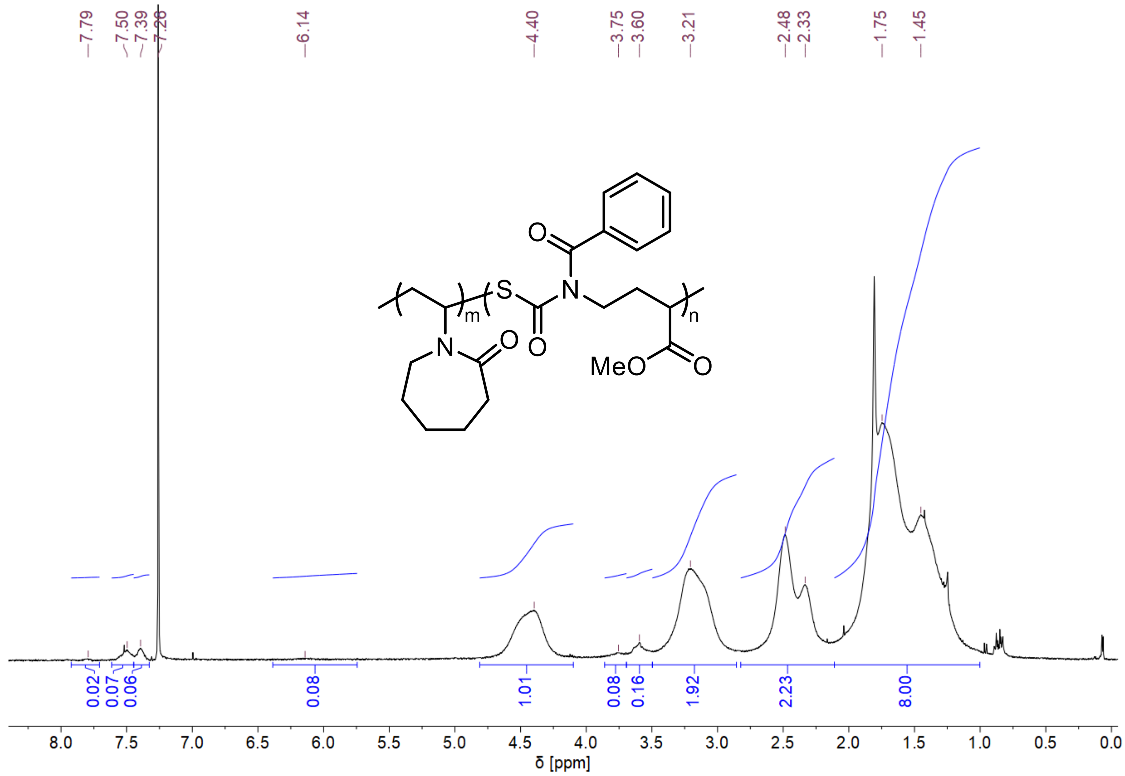

Figure S42. ^{1}H NMR spectrum of **PhCTC–*co*–PVCl** ($CDCl_3$, 400.1 MHz) *) indicates residual hexanes and grease.

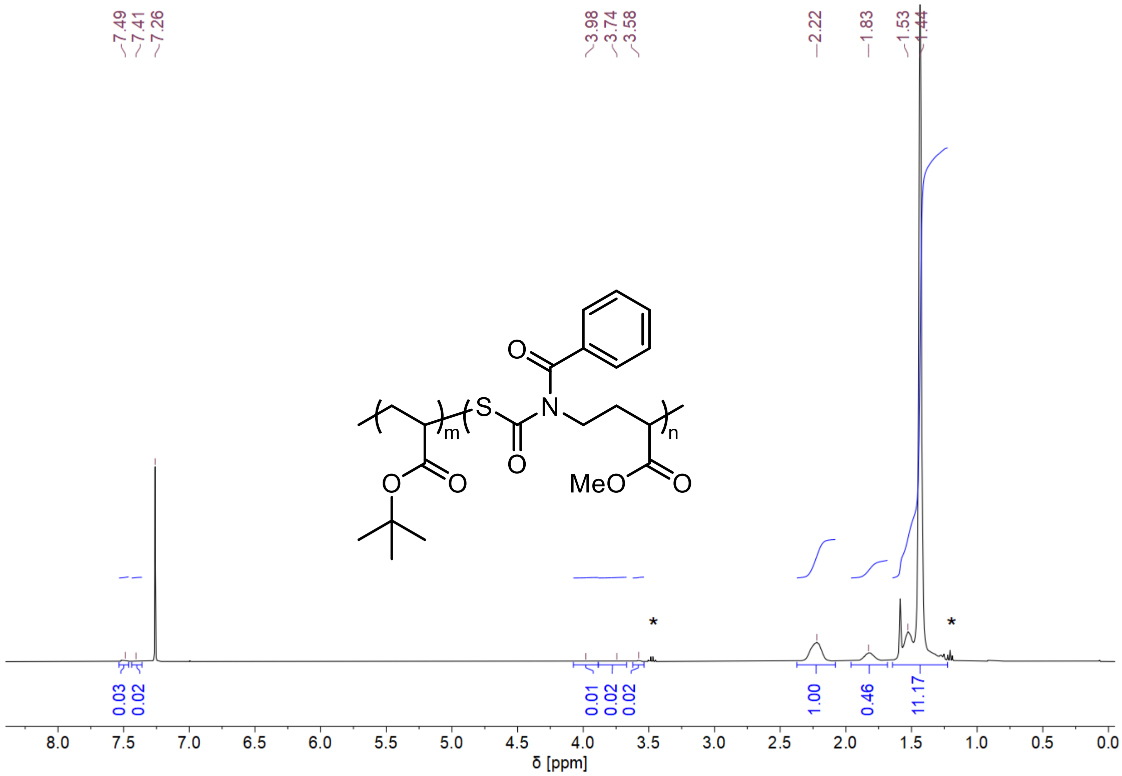

Figure S43. ^{1}H NMR spectrum of **PhCTC–*co*–P^{t}BuA** (CDCl$_3$, 400.1 MHz) *) indicates residual diethyl ether.

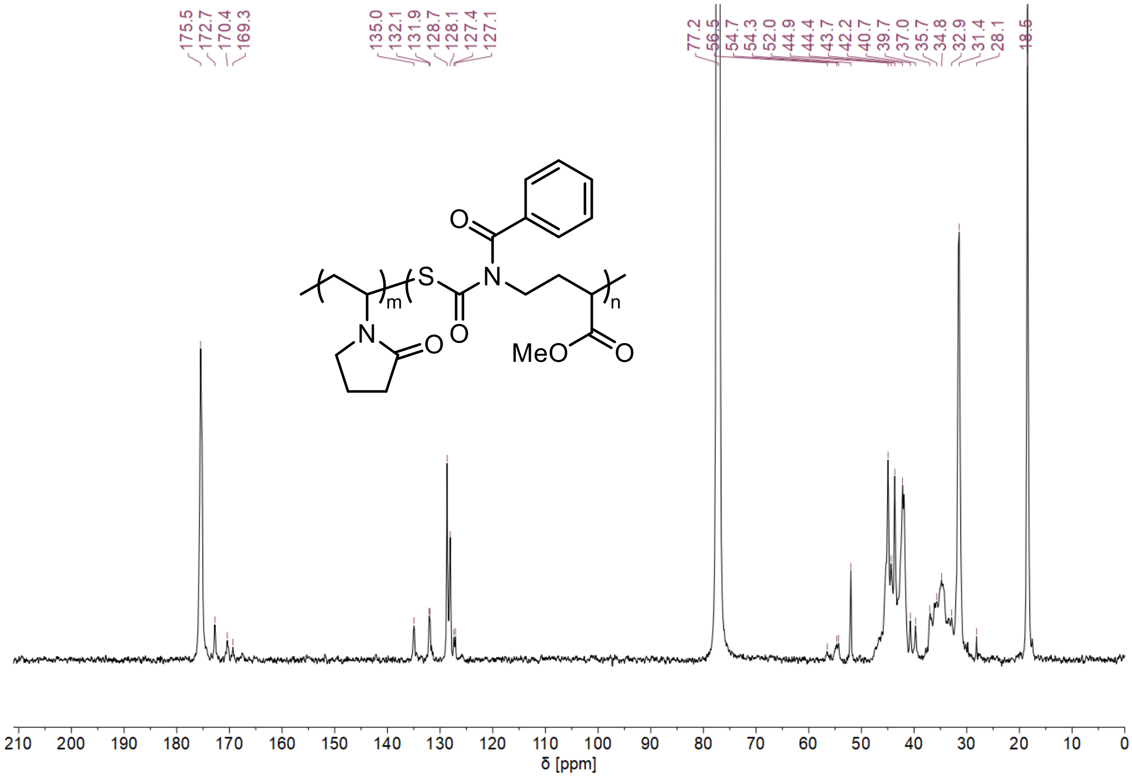

Figure S44. ^{13}C NMR spectrum of **PhCTC–*co*–PVP** (CDCl$_3$, 125.8 MHz).

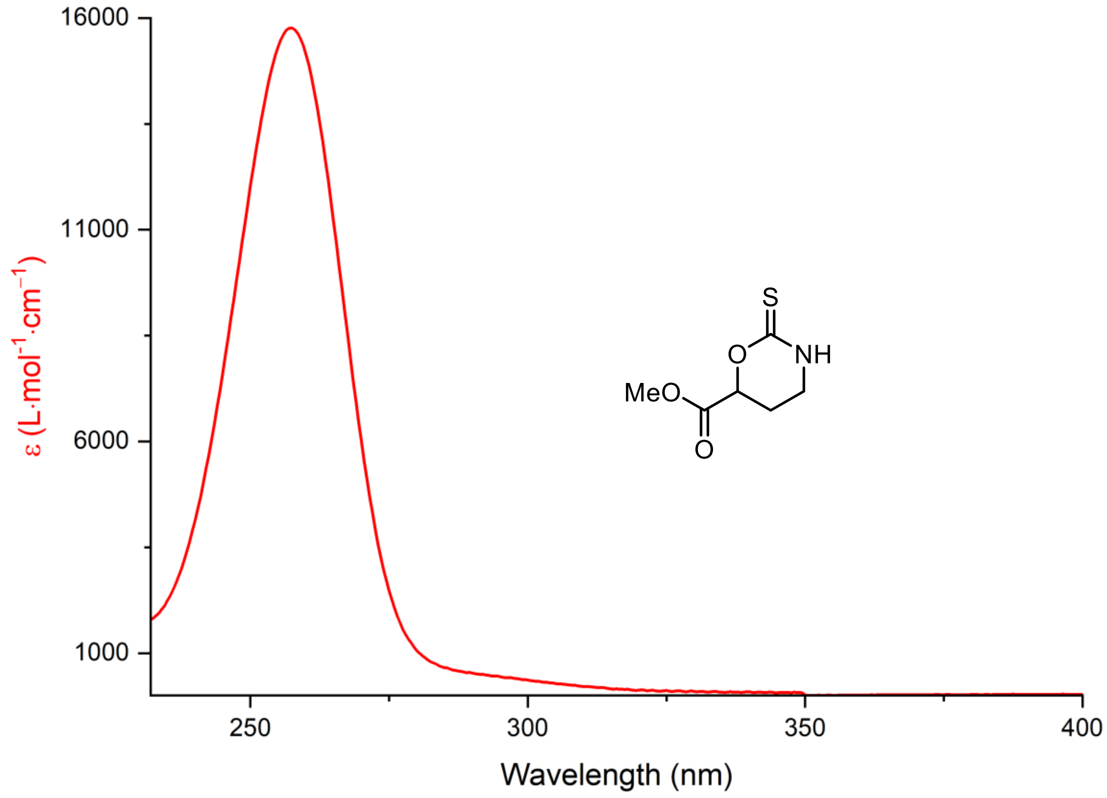

Figure S45. UV-Vis spectrum of **II** in CH_2Cl_2.

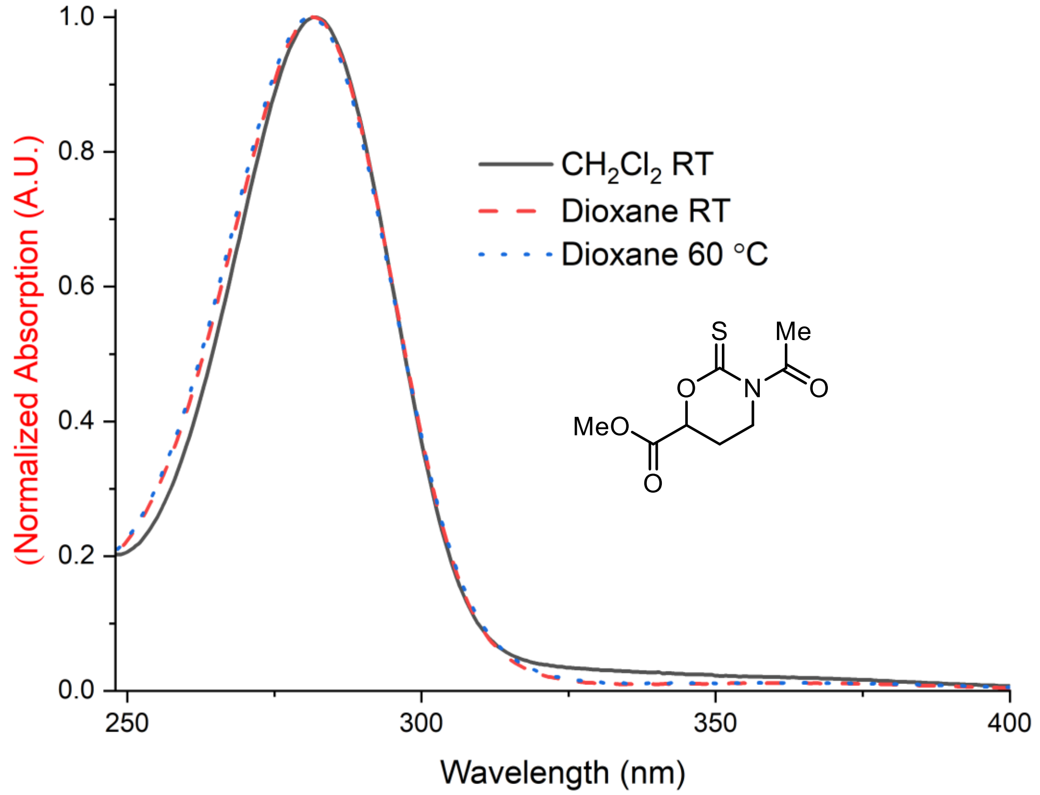

Figure S46. Normalized VT UV-Vis spectrum of **MeCTC** in CH_2Cl_2 and dioxane.

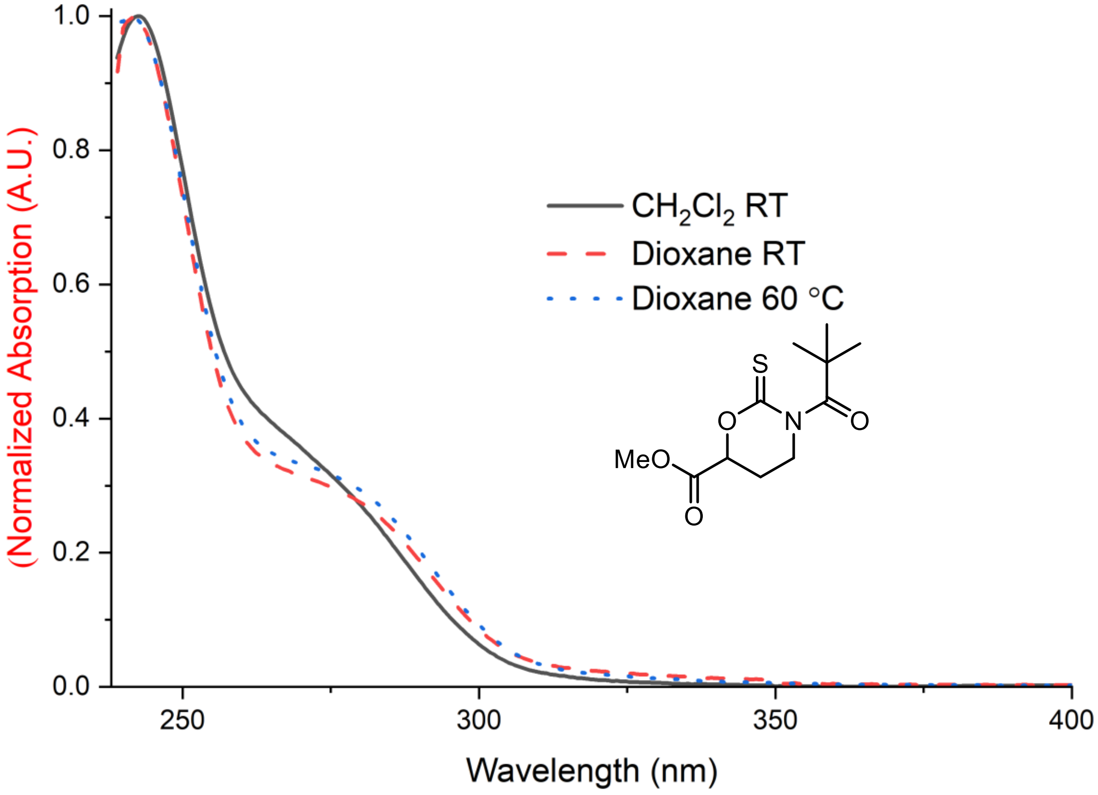

Figure S47. Normalized VT UV-Vis spectrum of ***t*BuCTC** in CH_2Cl_2 and dioxane.

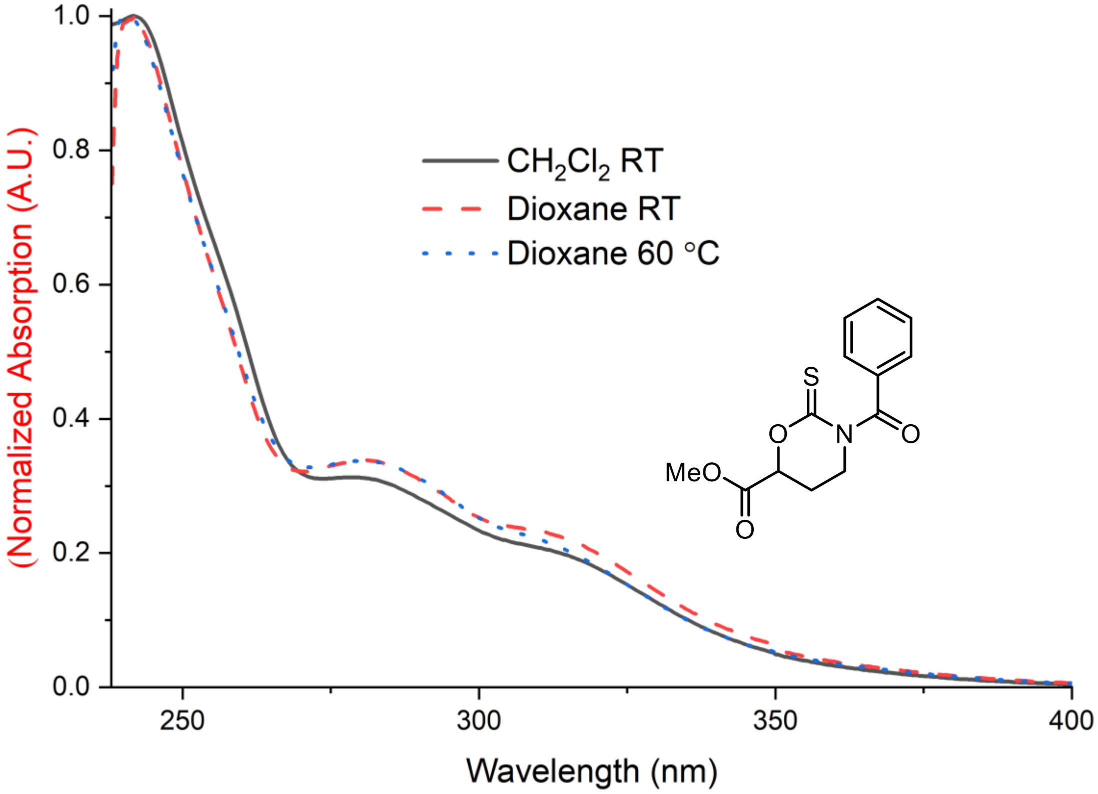

Figure S48. Normalized VT UV-Vis spectrum of **PhCTC** in CH_2Cl_2 and dioxane.

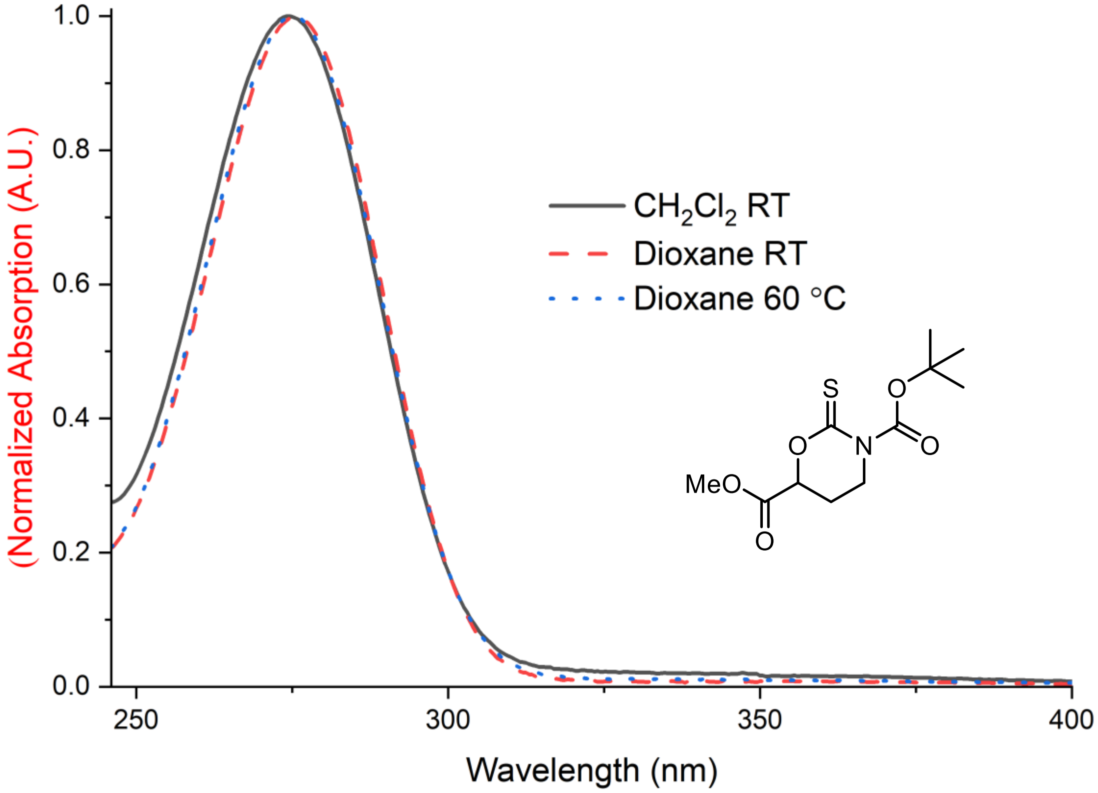

Figure S49. Normalized VT UV-Vis spectrum of ***t*BuOCTC** in CH_2Cl_2 and dioxane.

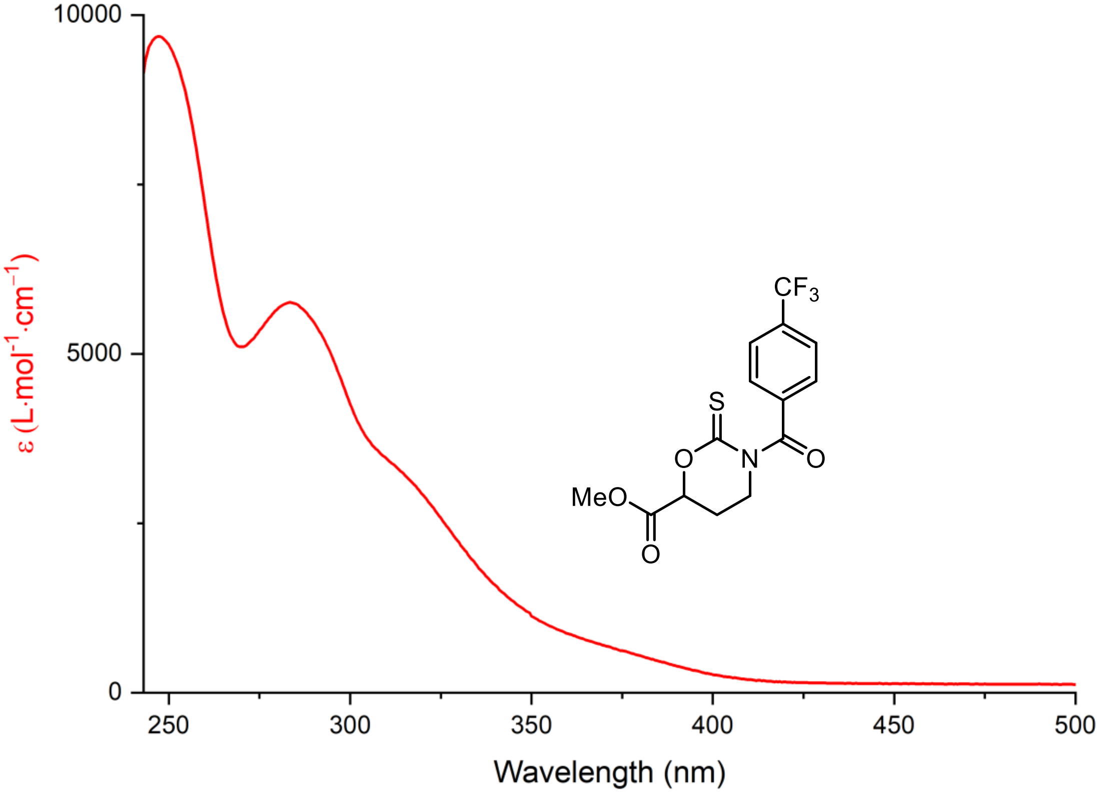

Figure S50. UV-Vis spectrum of ***p*–CF_3–PhCTC** in dioxane.

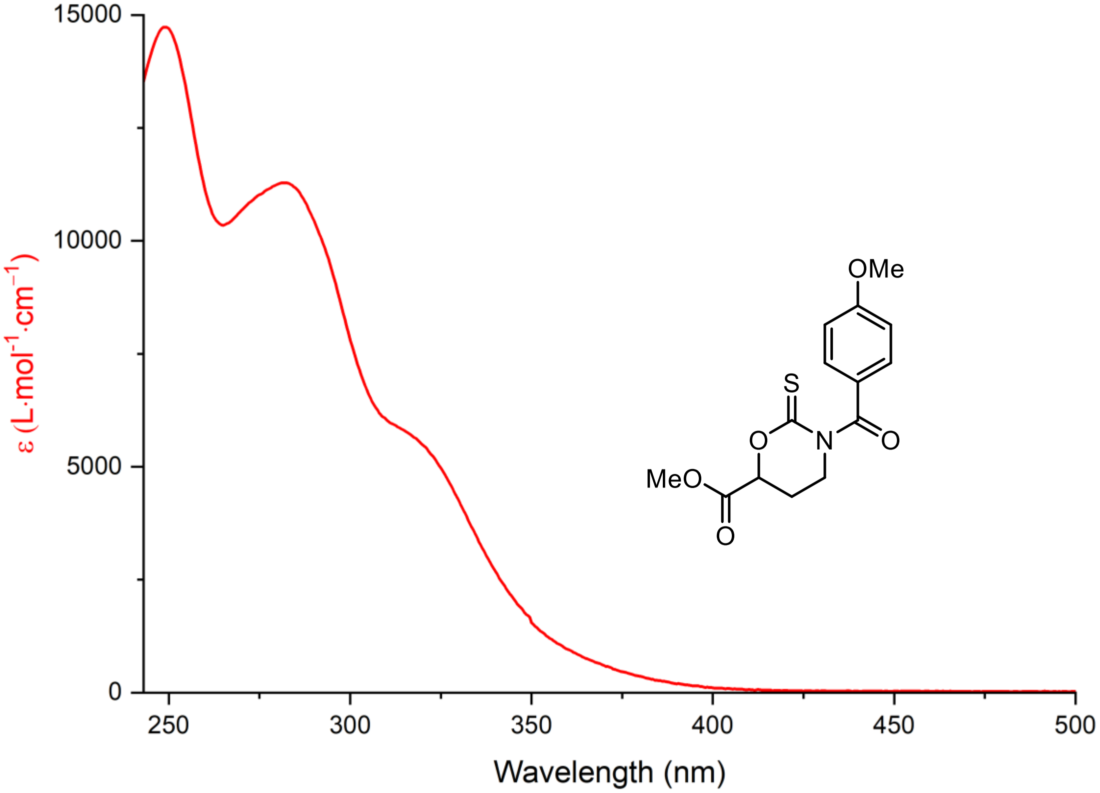

Figure S51. UV-Vis spectrum of ***p*–MeO–PhCTC** in dioxane.

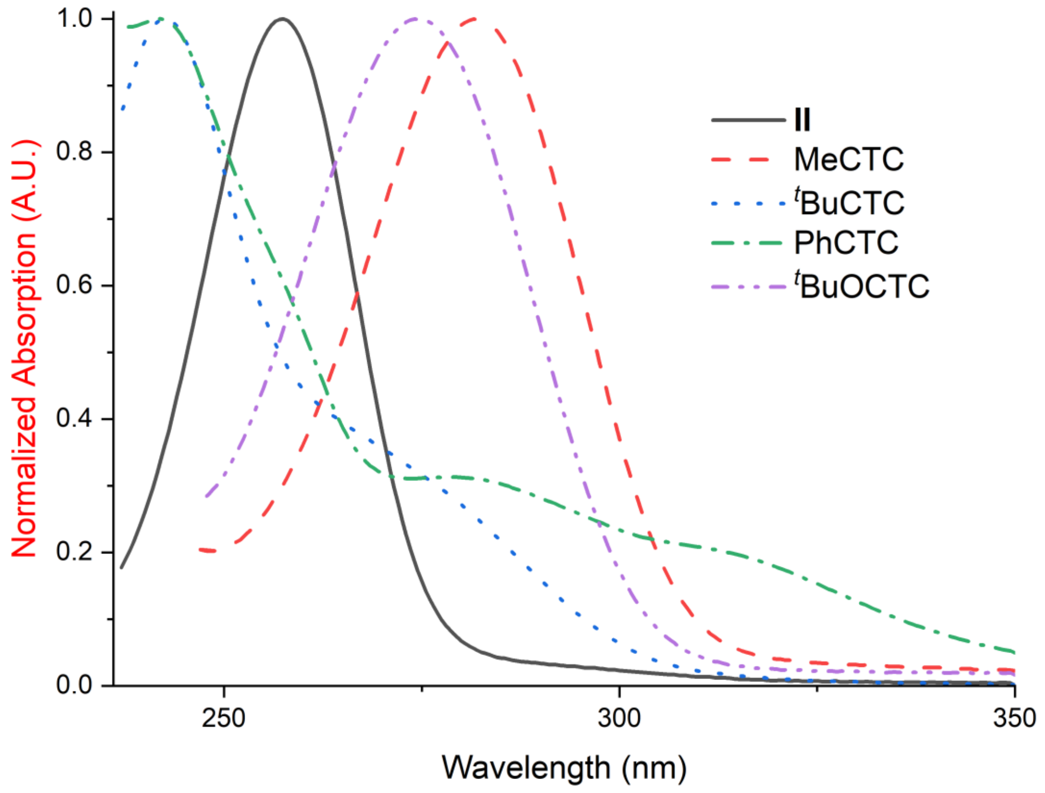

Figure S52. Normalized UV-Vis spectrum of CTCs.

Table S14. RAFT copolymerization of CTC and NVP with **R_1–R_5**.

Entry	CTC:NVP	CTC	M	RAFT	CTC Conv (%)	M Conv (%)	M_n (kg/mol)	$Đ$
1	5:100	Me	NVP	R1	55	22	2.6	1.71
2	5:100	Ph	NVP	R1	68	57	1.3	1.63
3	5:100	Ph	NVP	R2	70	98	2.2	1.67
4	5:100	Ph	NVP	R3	>98	86	4.5	1.67
5	5:100	Ph	NVP	R4	75	81	3.2	1.83
6	5:100	Ph	NVP	R5	64	78	2.2	1.76

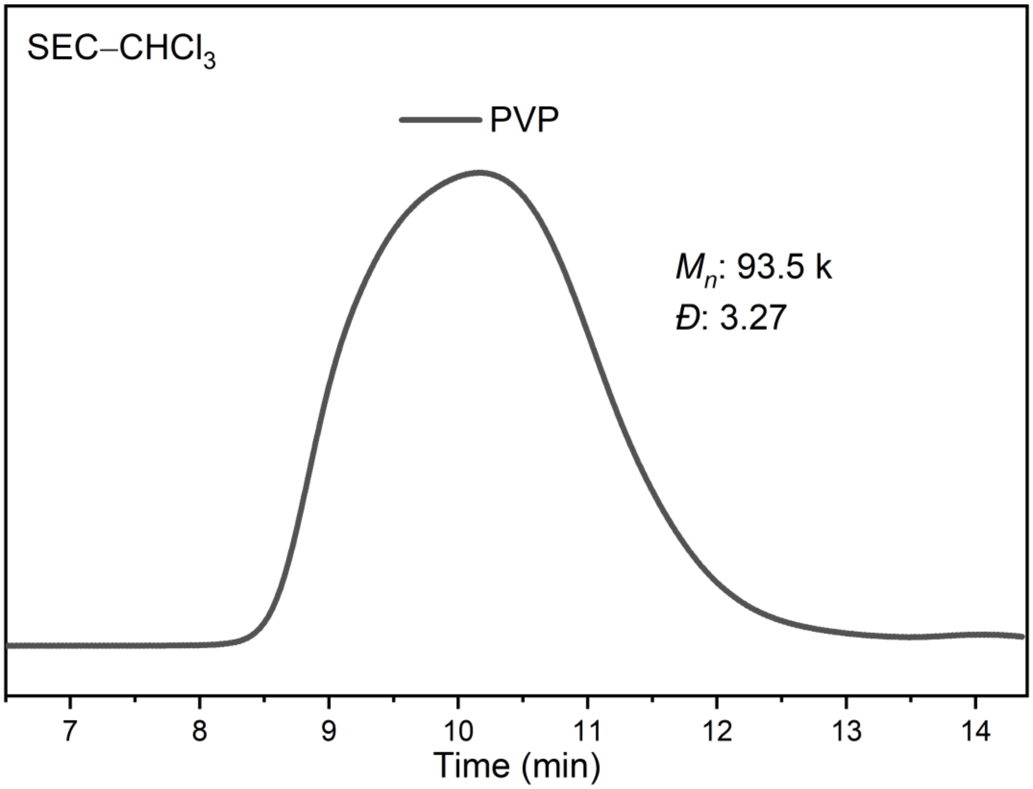

Figure S53. SEC trace of **PVP**.

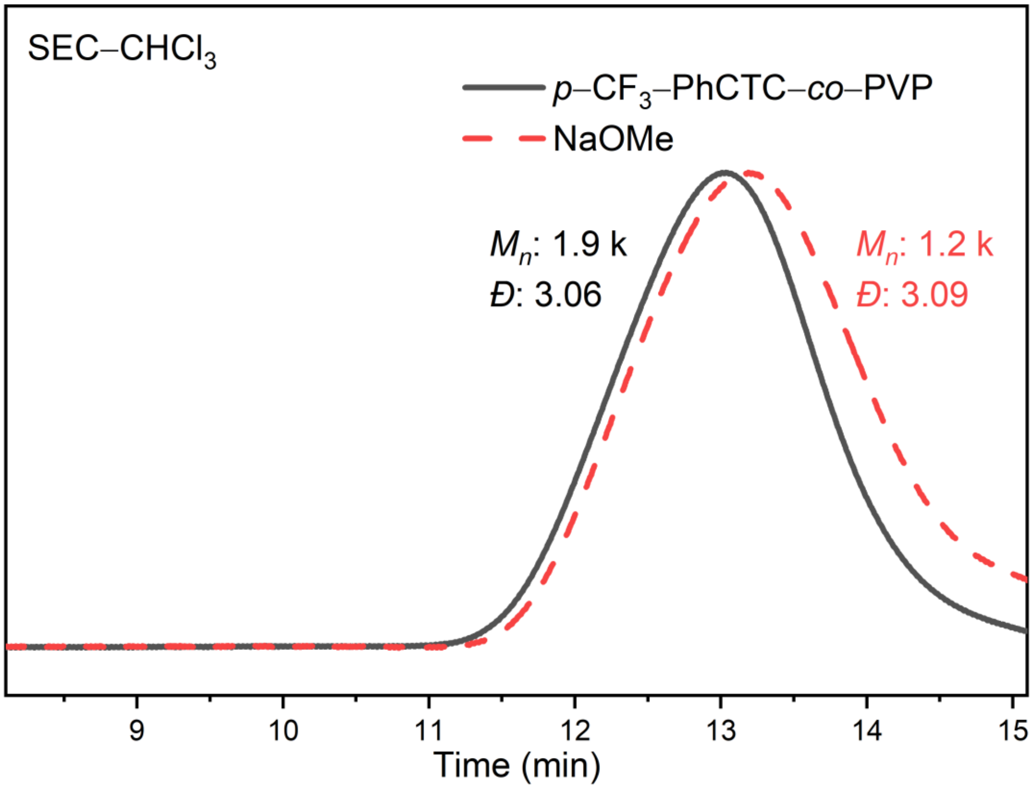

Figure S54. SEC trace of ***p*–CF$_3$–PhCTC–*co*–PVP**.

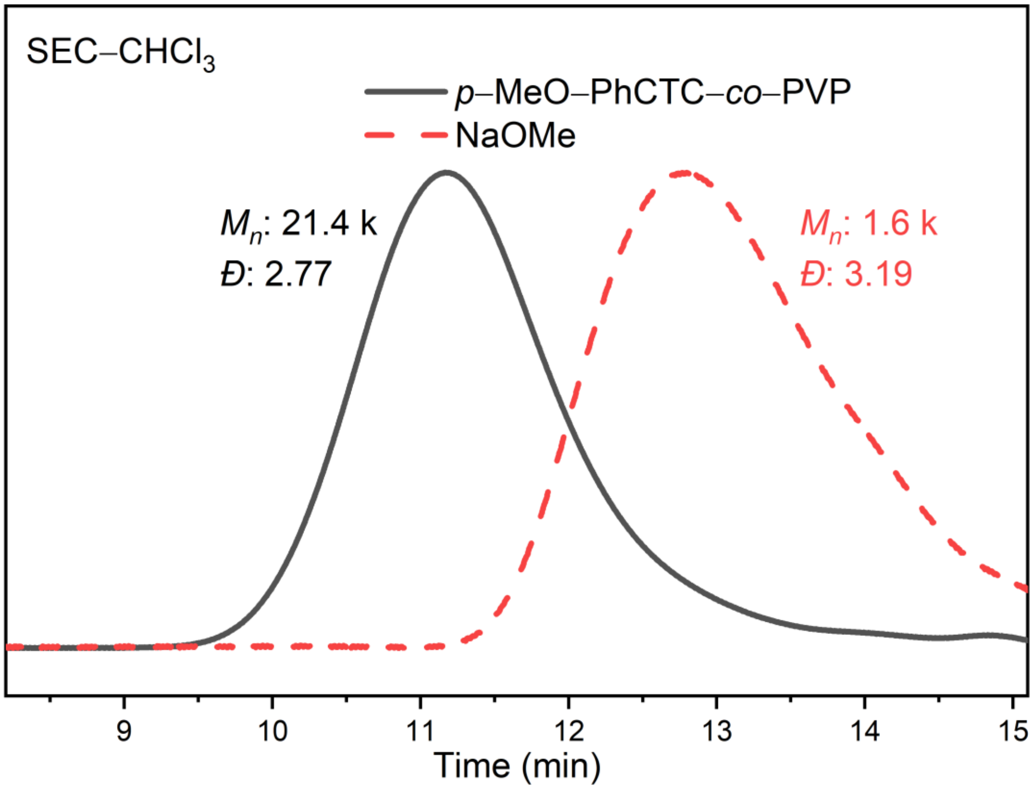

Figure S55. SEC trace of ***p*–MeO–PhCTC–*co*–PVP**.

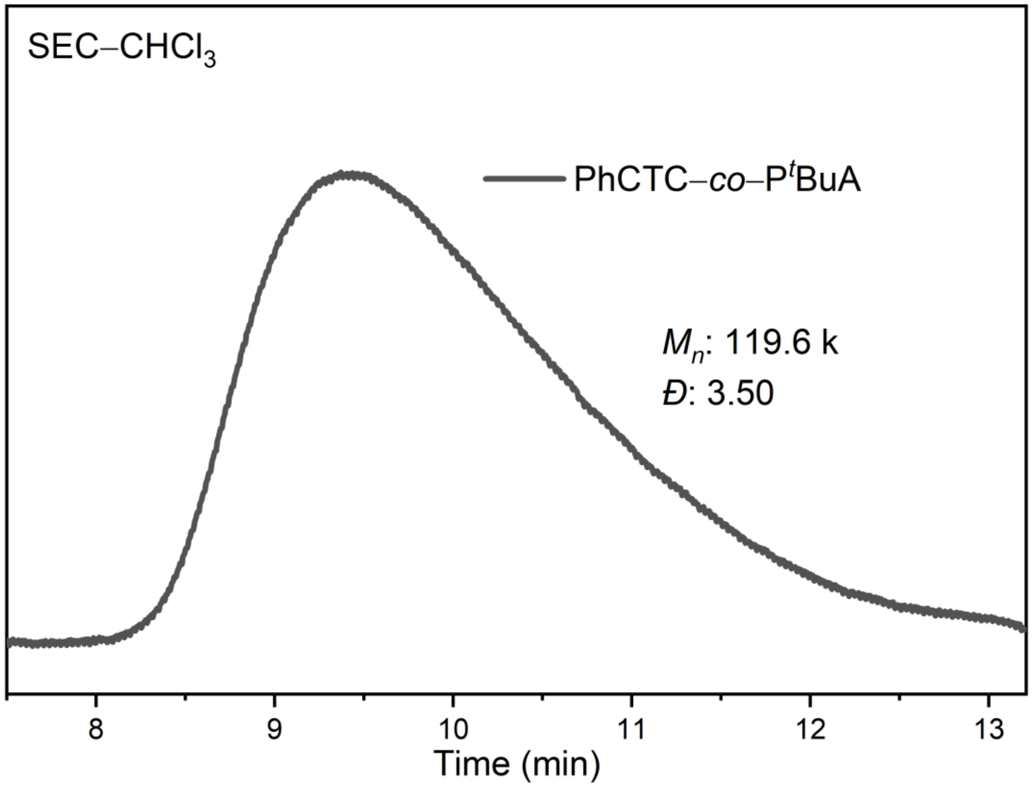

Figure S56. SEC trace of **PhCTC–*co*–P^{t}BuA**.

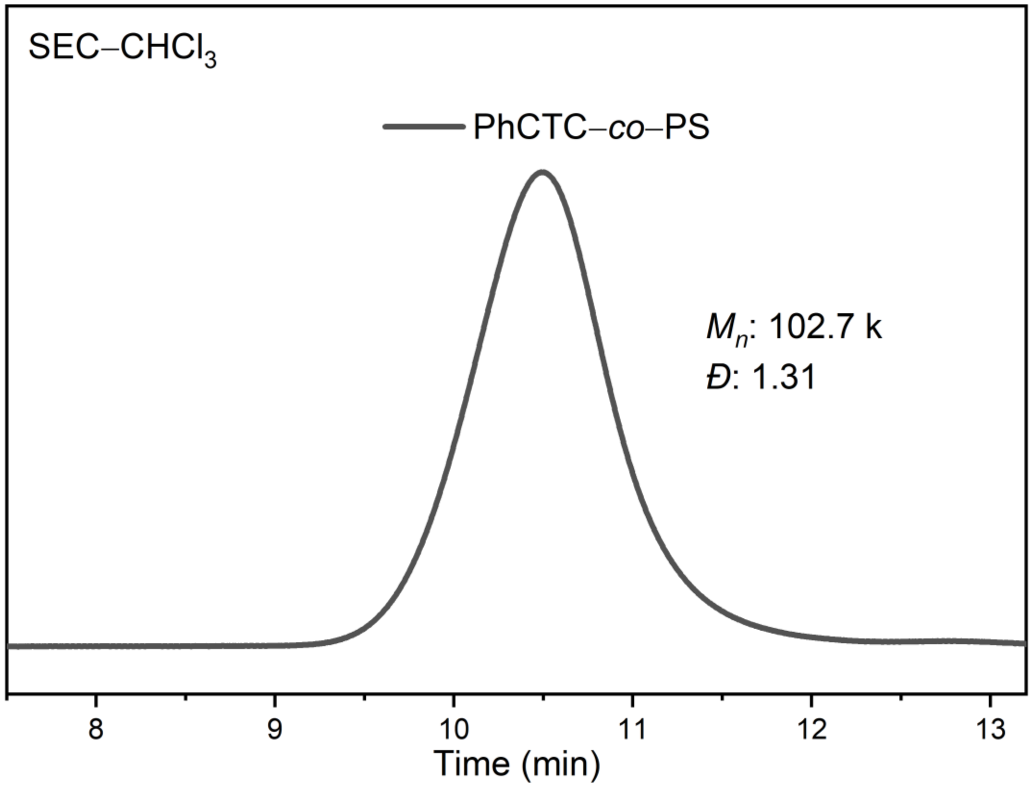

Figure S57. SEC trace of **PhCTC–*co*–PS**.

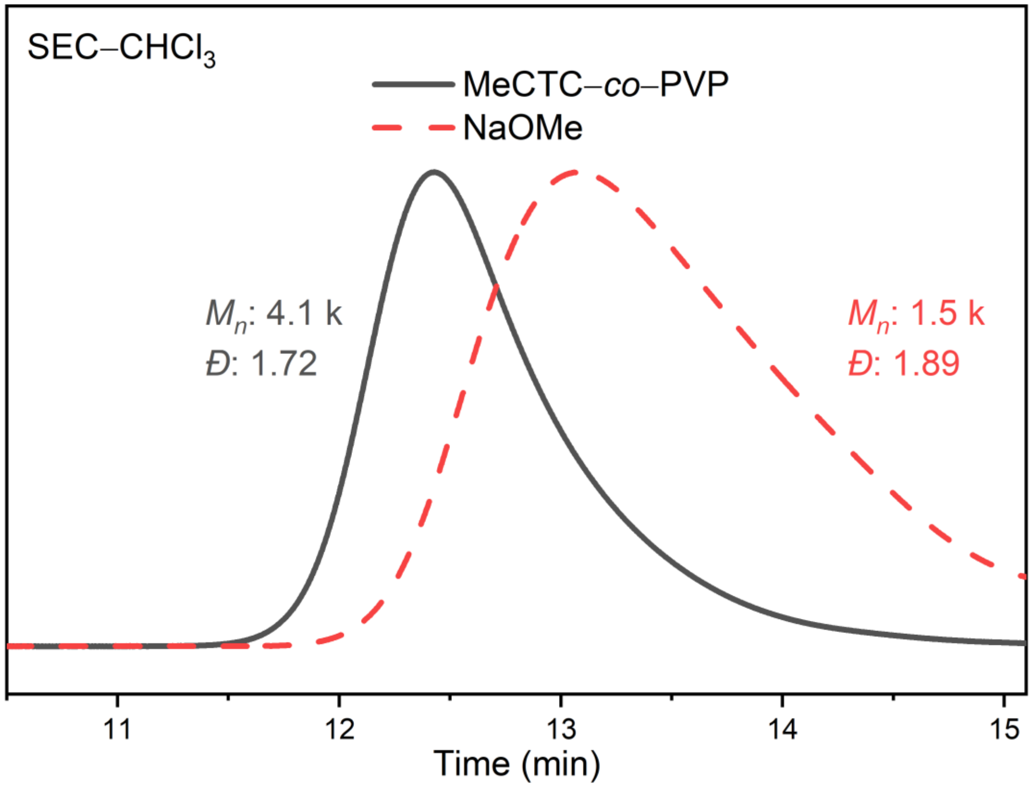

Figure S58. SEC trace of RAFT **MeCTC–*co*–PVP**.

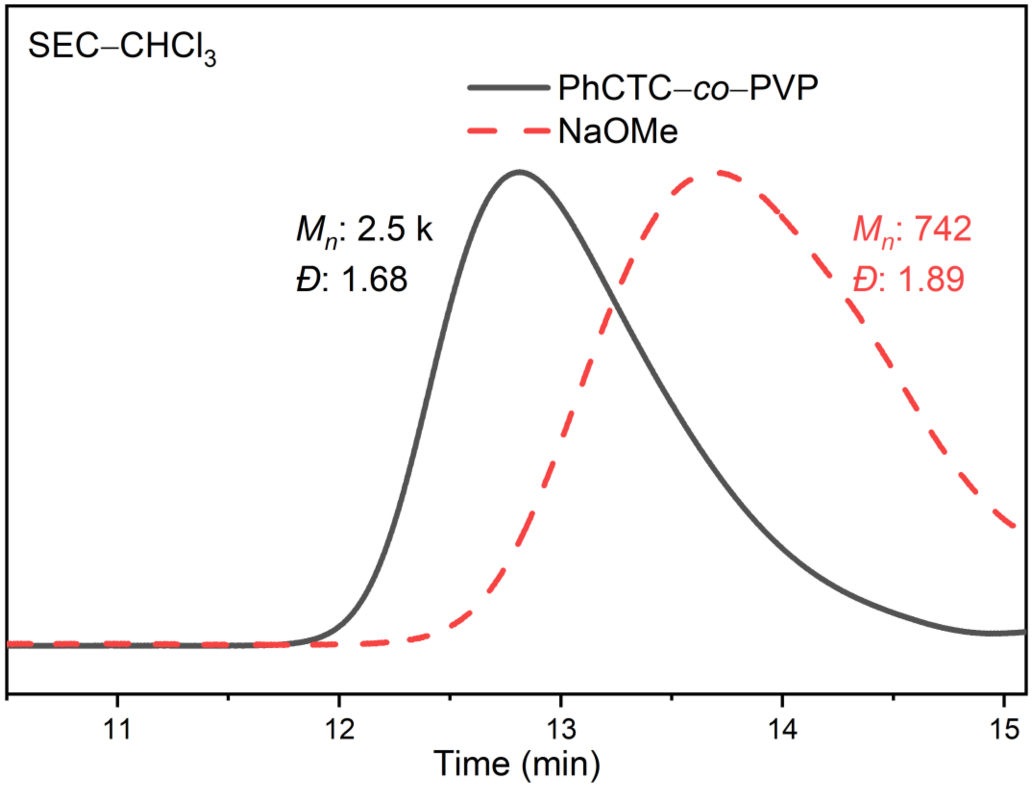

Figure S59. SEC trace of **PhCTC–*co*–PVP**.

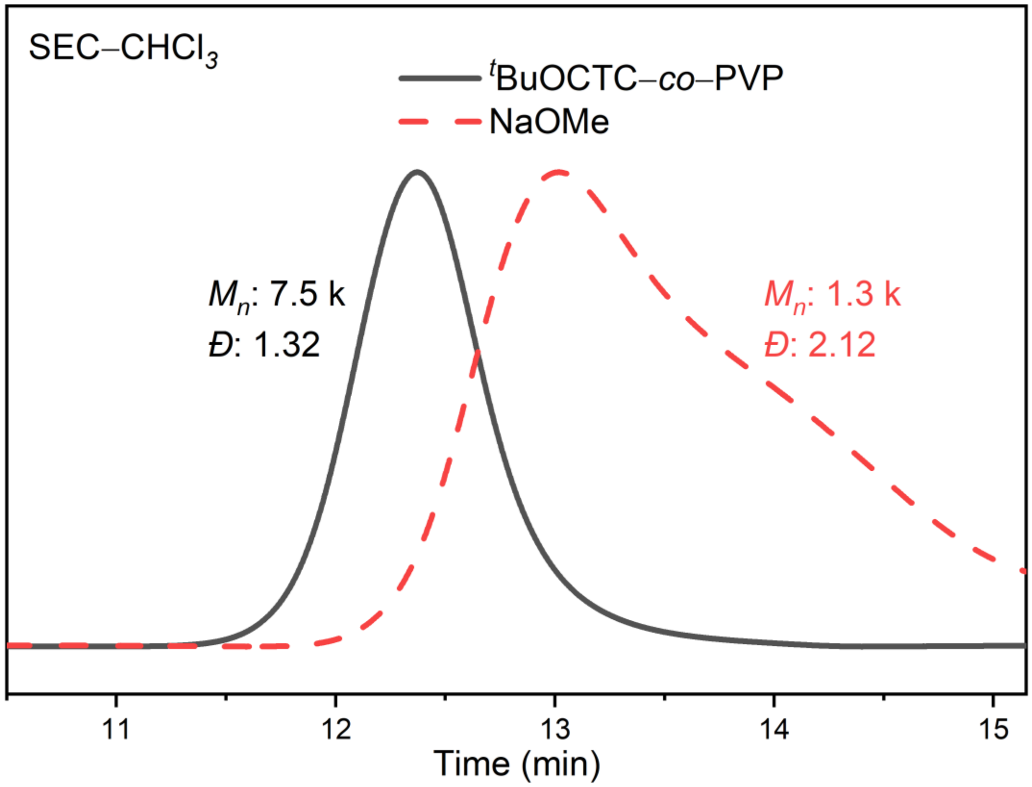

Figure S60. SEC trace of RAFT ***t*BuOCTC–*co*–PVP**.

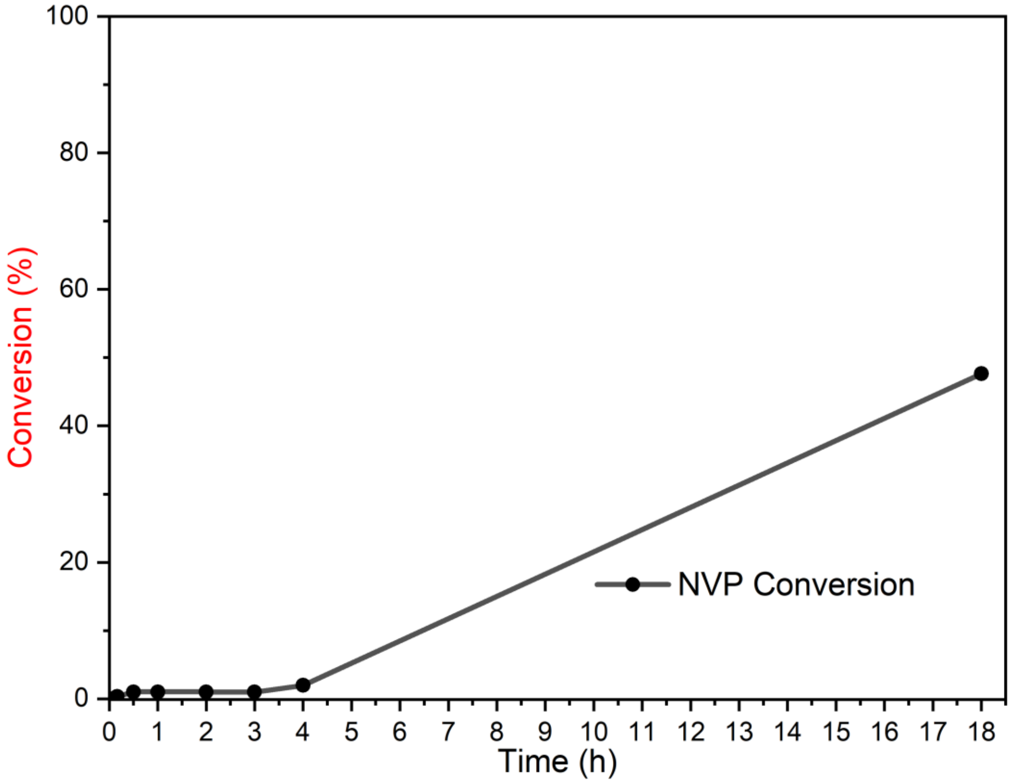

Figure S61. Conversion profile of **PhCTC–*co*–PVC**.

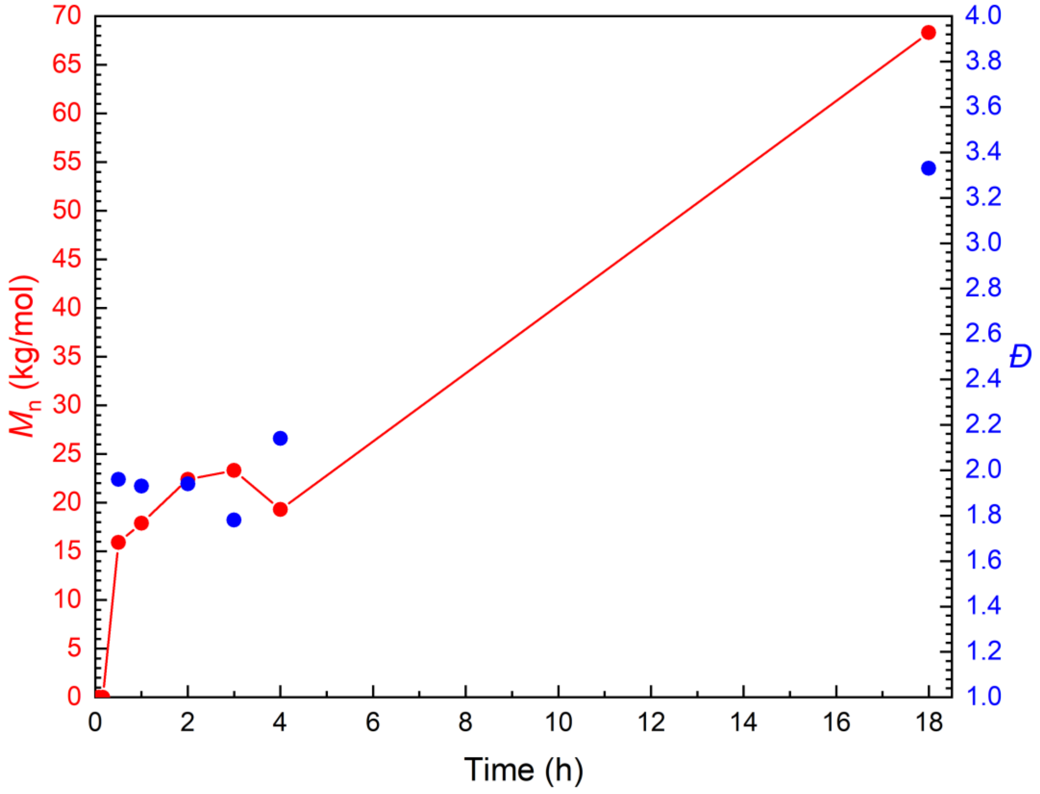

Figure S62. M_n and *Đ* profile of **PhCTC–*co*–PVC**.

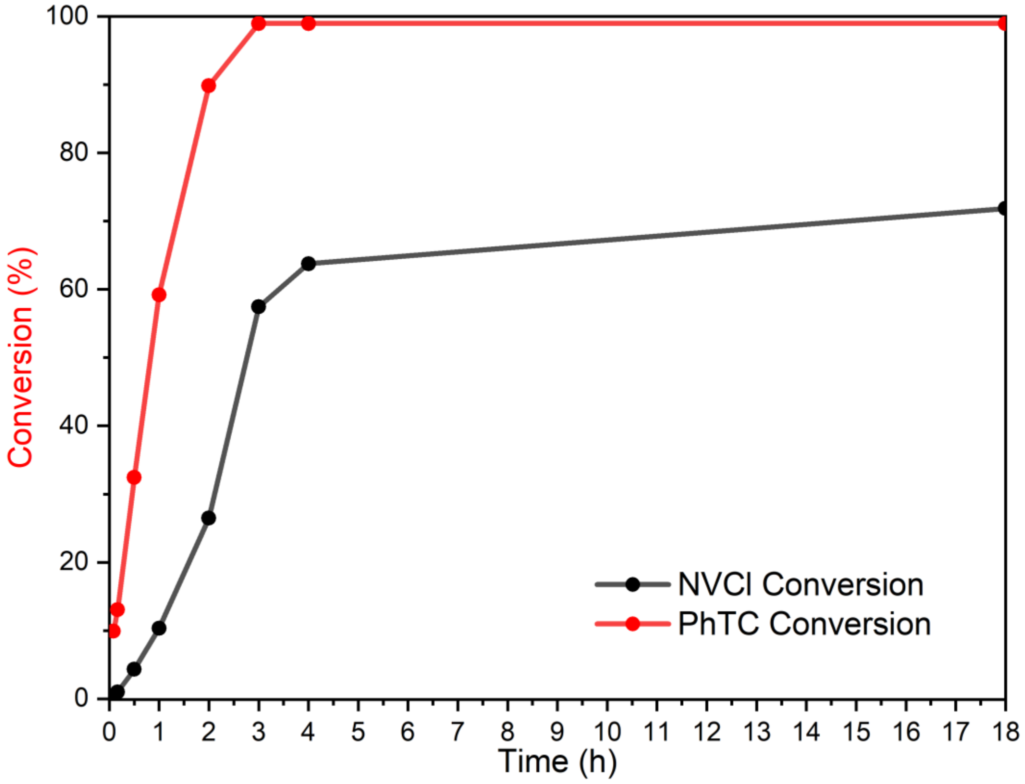

Figure S63. Conversion profile of **PhCTC–*co*–PVCL**.

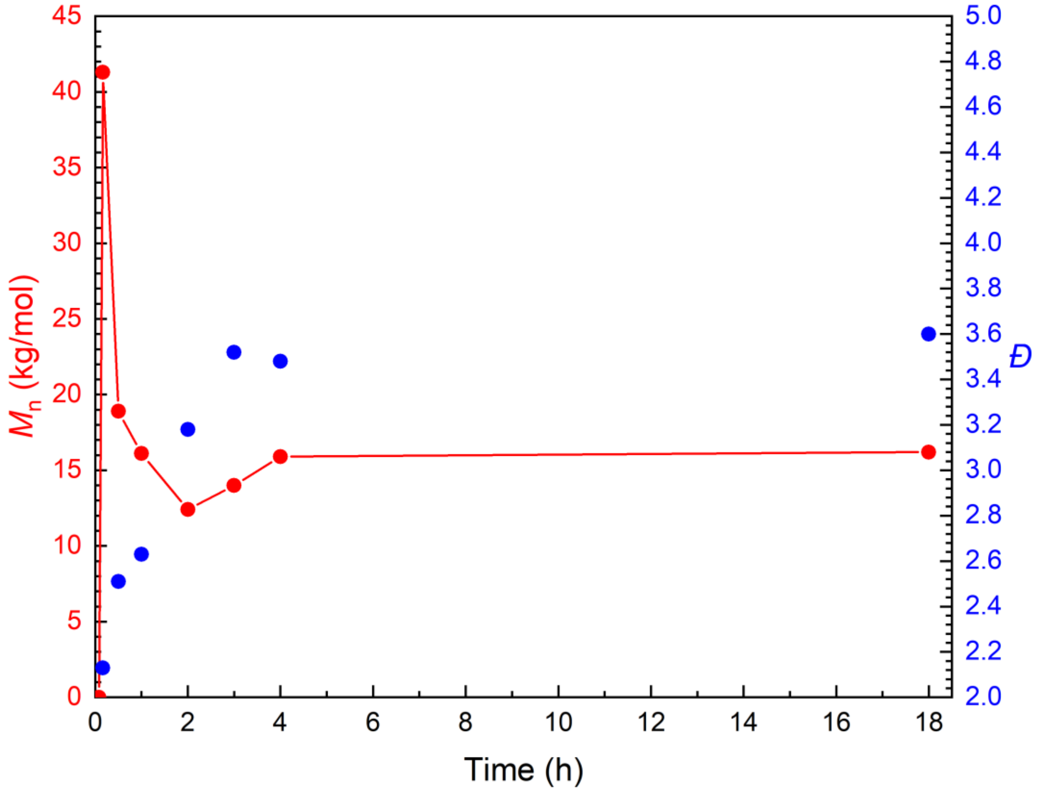

Figure S64. M_n and $Đ$ profile of **PhCTC–*co*–PVCL**.

References

1. Maiti, D.; Fors, B. P.; Henderson, J. L.; Nakamura, Y.; Buchwald, S. L. Palladium-catalyzed coupling of functionalized primary and secondary amines with aryl and heteroaryl halides: two ligands suffice in most cases. *Chem. Sci.* **2011**, *2* (1), 57-68.

2. Chakraborti, G.; Paladhi, S.; Mandal, T.; Dash, J. "On Water" Promoted Ullmann-Type C–N Bond-Forming Reactions: Application to Carbazole Alkaloids by Selective N-Arylation of Aminophenols. *J. Org. Chem.*, **2018**, *83*, 7347-7359.

3. Keddie, D. J.; Guerrero-Sanchez, C.; Moad, G.; Mulder, R. J.; Rizzardo, E.; Thang, S. H. Chain Transfer Kinetics of Acid/Base Switchable N-Aryl-N-Pyridyl Dithiocarbamate RAFT Agents in Methyl Acrylate, N-Vinylcarbazole and Vinyl Acetate Polymerization. *Macromolecules*, **2012**, *45* (10), 4205-4215.

4. Krause, L.; Herbst-Irmer, R.; Sheldrick, G.; Stalke, D. Comparison of silver and molybdenum microfocus X-ray sources for single-crystal structure determination. *J. Appl.Crystallogr*. **2015**, *48*, 3-10.

5. Bruker, V8.40B ed., Bruker AXS Inc., Madison, Wisconsin, USA.

6. Sheldrick, G. SHELXT – Integrated space-group and crystal-structure determination. *Acta Crystallogr. A*, **2015**, *71*, 3-8.

7. Dolomanov, O.; Bourhis, L.; Gildea, R.; Howard, J.; Puschmann, H. OLEX2: a complete structure solution, refinement and analysis program. *J. Appl. Crystallogr*. **2009**, *42*, 339-341.

8. Frisch, M. J.; Trucks, G. W.; Schlegel, H. B.; Scuseria, G. E.; Robb; M. A.; Cheeseman, J. R.; Scalmani, G.; Barone, V.; Mennucci, B.; Petersson, G. A.; Nakatsuji, H.; Caricato, M.; Li, X.; Hratchian, H. P.; Izmaylov, A. F.; Bloino, J.; Zheng, G.; Sonnenberg, J. L.; Hada, M.; Ehara, M.; Toyota, K.; Fukuda, R.; Hasegawa, J.; Ishida, M.; Nakajima, T.; Honda, Y.; Kitao, O.; Nakai, H.; Vreven, T.; Montgomery, J. A.; Jr., Peralta, J. E.; Ogliaro, F.; Bearpark, M.; Heyd, J. J.; Brothers, E.; Kudin, K. N.; Staroverov, V. N.; Kobayashi, R.; Normand, J.; Raghavachari, K.; Rendell, A.; Burant, J. C.; Iyengar, S. S.; Tomasi, J.; Cossi, M.; Rega, N.; Millam, J. M.; Klene, M.; Knox, J. E.; Cross, J. B.; Bakken, V.; Adamo, C.; Jaramillo, J.; Gomperts, R.; Stratmann, R. E.; Yazyev, O.; Austin, A. J.; Cammi, R.; Pomelli, C.; Ochterski, J. W.; Martin, R. L.; Morokuma, K.; Zakrzewski, V. G.; Voth, G. A.; Salvador, P.; Dannenberg, J. J.; Dapprich, S.; Daniels, A. D.; Farkas, O.; Foresman, J. B.; Ortiz, J. V.; Cioslowski, J.; and Fox, D. J. Gaussian 09, revision D.01; Gaussian Inc.: Wallingford, CT, 2013.

9. Becke, A. D. Density-functional thermochemistry. III. The role of exact exchange. *J. Chem. Phys.* **1993**, *98*, 5648-5652.

10. Lee, C.; Yang, W.; Parr, R. G. Development of the Colle-Salvetti correlation-energy formula into a functional of the electron density. *Phys. Rev. B* **1988**, *37,* 785-789.

11. McLean, A. D.; Chandler, G. S. Contracted Gaussian basis sets for molecular calculations. I. Second row atoms, Z=11–18. *J. Chem. Phys.* **1980**, *72*, 5639-5648.

12. Zhao, Y.; Truhlar, D. G. The M06 suite of density functionals for main group thermochemistry, thermochemical kinetics, noncovalent interactions, excited states, and transition elements: two new functionals and systematic testing of four M06-class functionals and 12 other functionals. *Theor. Chem. Acc.* **2008**, *120*, 215-241.

13. Weigend, F.; Ahlrichs, R. Balanced basis sets of split valence, triple zeta valence and quadruple zeta valence quality for H to Rn: Design and assessment of accuracy. *Phys. Chem. Chem. Phys.* **2005**, *7*, 3297-3305.

14. Winkler, F. K.; Dunitz, J. D. *J. Mol. Biol.* **1971**, *59* (1), 169-182. DOI: https://doi.org/10.1016/0022-2836(71)90419-0.

15. Meng, G.; Zhang, J.; Szostak, M. *Chem. Rev.* **2021**, *121* (20), 12746-12783. DOI: 10.1021/acs.chemrev.1c00225.

16. Yamada, S. *Angew. Chemie. Int. Ed.* **1993**, *32* (7), 1083-1085. DOI: https://doi.org/10.1002/anie.199310831 (acccessed 2023/11/21).

17. Yamada, S.; Matsuda, K. *Chem. Lett.* **2001**, *30* (8), 750-751. DOI: 10.1246/cl.2001.750 (acccessed 2023/11/21).

18. Kazak, C.; Yilmaz, V. T.; Servi, S.; Koca, M.; Heinemann, F. W. *Acta Cryst. C* **2005**, *61* (6), o348-o350. DOI: doi:10.1107/S0108270105009844.
